# Revolutionizing Free-Space Optics: A Survey of Enabling Technologies, Challenges, Trends, and Prospects of Beyond 5G Free-Space Optical (FSO) Communication Systems

**DOI:** 10.3390/s24248036

**Published:** 2024-12-16

**Authors:** Isiaka A. Alimi, Paulo P. Monteiro

**Affiliations:** 1 Instituto de Telecomunicações, University of Aveiro, 3810-193 Aveiro, Portugal; paulo.monteiro@ua.pt; 2 Department of Electronics, Telecommunications and Informatics, University of Aveiro, 3810-193 Aveiro, Portugal

**Keywords:** 5G, 6G, adaptive optics, advanced modulation schemes, aperture averaging, artificial intelligence, atmospheric turbulence, cooperative relaying, dual-hop transmission schemes, error correction codes, free-space optical communications, hybrid FSO system, machine learning, millimeter wave, pointing errors, spatial diversity, terahertz communications

## Abstract

As the demand for high-speed, low-latency communication continues to grow, free-space optical (FSO) communication has gained prominence as a promising solution for supporting the next generation of wireless networks, especially in the context of the 5G and beyond era. It offers high-speed, low-latency data transmission over long distances without the need for a physical infrastructure. However, the deployment of FSO systems faces significant challenges, such as atmospheric turbulence, weather-induced signal degradation, and alignment issues, all of which can impair performance. This paper offers a comprehensive survey of the enabling technologies, challenges, trends, and future prospects for FSO communication in next-generation networks, while also providing insights into the current mitigation strategies. The survey explores the critical enabling technologies such as adaptive optics, modulation schemes, and error correction codes that are revolutionizing FSO communication and addressing the unique challenges of FSO links. Also, the integration of FSO with radio frequency, millimeter-wave, and Terahertz technologies is explored, emphasizing hybrid solutions that enhance reliability and coverage. Additionally, the paper highlights emerging trends, such as the integration of FSO with artificial intelligence-driven optimization techniques and the growing role of machine learning in enhancing FSO system performance for dynamic environments. By analyzing the current trends and identifying key challenges, this paper emphasizes the prospects of FSO communication in the evolving landscape of 5G and future networks. In this regard, it assesses the potential of FSO to meet the demands for high-speed, low-latency communication and offers insights into its scalability, reliability, and deployment strategies for 5G and beyond. The paper concludes by identifying the open challenges and future research directions critical to realizing the full potential of FSO in next-generation communication systems.

## 1. Introduction

Free-space optical (FSO) communication systems are gaining significant attention due to their potential to offer high data rates, secure communication, and immunity to electromagnetic interference. Unlike traditional radio frequency (RF) systems, FSO utilizes light to transmit data through the atmosphere, making it an attractive solution for a variety of applications. The growing demand for higher bandwidth and the limitations of existing wireless communication technologies drive the exploration and development of FSO systems [1,2]. This section provides an overview of the fundamental principles, key advantages, and challenges associated with FSO communication, setting the stage for a deeper understanding of its potential and the ongoing research in this field.

### 1.1. Background and Motivation

With the growing demand for high-speed, low-latency communication in the era of 5G and beyond, and the huge capital expenditures associated with public works (such as trench digging and site restoration) limiting the widespread installation of optical fiber infrastructure, FSO communication has emerged as a potent technology poised to meet these demands [3]. FSO communication, which involves the transmission of data through the atmosphere using optical beam, offers an attractive alternative to traditional RF communication due to its potential for low latency, noise immunity, electromagnetic interference immunity, spatial reusability, ease of deployment, being license free, high security, reduced spectrum congestion, and the capability to deliver transmissions at extremely high data rates between two terminals [4,5], whether they are just a few meters apart or separated by thousands of kilometers (long operational range) [6]. FSO systems are especially advantageous for line-of-sight (LoS) communication, bridging gaps where traditional fiber optics or RF systems may not be feasible. For instance, FSO systems utilize narrow laser beams with directional properties. This spatial confinement allows for a high degree of spatial reuse and makes eavesdropping difficult, inherently enhancing security. Utilizing light as the carrier in FSO communications ensures immunity to electromagnetic interference. Additionally, FSO systems are easy to deploy and can be reinstalled without incurring the costs associated with dedicated fiber optic connections. Moreover, the frequency used by FSO technology exceeds 300 GHz, which is unlicensed globally, thus eliminating the need for license fees [1,2,7].

**Figure 1:**
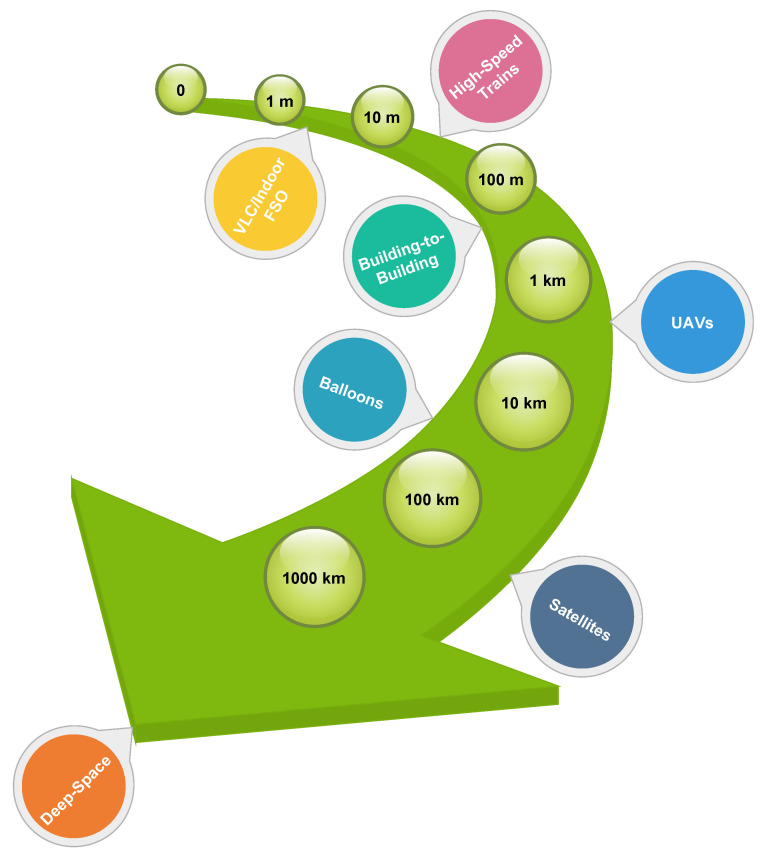
Potential applications of FSO systems based on the operational distance between transceivers.

**Figure 2:**
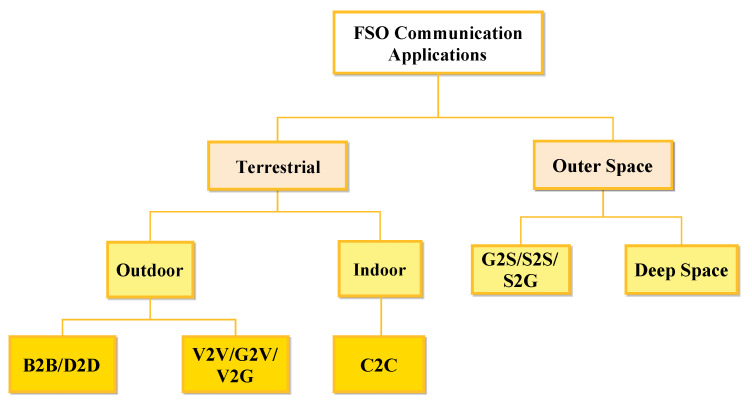
Categorization of FSO communication systems based on their applications. B2B: Building-to-Building, D2D: Device-to-Device, C2C: Chip-to-Chip, V2V: Vehicle-to-Vehicle, V2G: Vehicle-to-Ground, G2V: Ground-to-Vehicle, S2S: Satellite-to-Satellite, S2G: Satellite-to-Ground, and G2S: Ground-to-Satellite.

Frequencies in FSO communications are significantly higher than those in RF communications. Consequently, FSO communications can achieve much higher data rates compared to RF communications, and they require antennas that take up less space. Additionally, the coherence of laser light in FSO links can decrease geometrical loss, allowing the transmission of high data rates over long distances [7]. Figure 1 illustrates various FSO applications along with their respective communication ranges. The capability to transmit data over long distances without the need for physical infrastructure makes FSO a suitable candidate for supporting next-generation wireless networks, including 5G and beyond [8]. In this context, the absence of a need for optical fibers between communication nodes allows for flexible application across various use cases and high-speed applications including unmanned aerial vehicles (UAVs), intra- and inter-datacenter connections, inter-building links, high-speed trains, 5G fronthaul and backhaul for wireless cellular networks, indoor and outdoor local- and wide-area networks, metropolitan area network extensions, urban connectivity, last-mile connectivity, inter-chip connectivity, fiber backup, satellite communications, deep-space communications, underwater communication, disaster recovery, video surveillance, and redundant link [2,9,10]. Additionally, it is regarded as a promising solution for addressing the significant traffic demands of sixth-generation (6G) systems [11,12]. These use cases can be classified into two main categories, terrestrial and outer space FSO communications, as illustrated in Figure 2. Given its benefits, FSO is being explored as a solution for last-mile connectivity in situations where fiber deployment is challenging and RF systems fall short of meeting bandwidth needs. Additionally, these advantages position FSO as a highly promising solution for space communications. In this context, by leveraging its strengths, FSO can address many of the challenges associated with traditional communication methods, providing a more efficient and reliable means of data transmission between spacecraft, satellites, and ground stations [13].

Although FSO technology offers undeniable advantages and has a broad range of potential applications, significant technical challenges impede its widespread adoption [14]. For terrestrial applications, atmospheric conditions such as rain, fog, and turbulence can significantly degrade the signal, leading to reduced performance. Furthermore, for general applications, alignment issues and the need for precise beam pointing add to the complexity of FSO system deployment. Addressing these challenges requires the development of enabling technologies and innovative strategies to ensure the reliable performance of FSO systems in diverse environments [2,15].

### 1.2. Objectives and Scope of the Paper

This paper aims to provide a comprehensive survey of the enabling technologies, challenges, trends, and future prospects of FSO communication in the context of 5G and beyond. The key objectives include the following:

(i) Analysis of Current Challenges: examination of the major challenges faced by FSO systems, including atmospheric turbulence (AT), weather-induced signal degradation, and alignment issues.
(ii) Review of Enabling Technologies: exploration of critical technologies such as adaptive optics (AO), advanced modulation schemes, aperture averaging, spatial diversity, cooperative relaying, hybrid FSO system, and error correction codes (ECCs) that are instrumental in overcoming the challenges associated with FSO communication.
(iii) Exploration of Emerging Trends: discussion of emerging trends, such as the integration of FSO with RF, millimeter-wave (mmWave), and Terahertz (THz) technologies, as well as the use of artificial intelligence (AI) and machine learning (ML) to optimize FSO performance.
(iv) Prospects for 5G and Beyond: assessment of the potential of FSO communication to meet the demands of next-generation networks, including its scalability, reliability, and deployment strategies.
(v) Future Research Directions: identification of open challenges and future research directions necessary to realize the full potential of FSO in the evolving landscape of communication technologies.

The aforementioned objectives highlight the involvement of numerous phenomena in FSO communication, which must be meticulously analyzed to ensure a reliable communication system.

### 1.3. Contributions

There have been considerable contributions in the literature to FSO communications. Nevertheless, the usual space constraints for publications prevented comprehensive research work in which assiduous efforts towards FSO communications development can be presented. Therefore, an all-inclusive survey in which the trends in FSO communication systems can be studied is highly imperative. Based on this, apart from being a good complement to contributions of the existing work, this paper also offers in-depth clarifications regarding different related concepts and architectures. In this regard, the main contributions of this tutorial paper are as follows:

(i) Comprehensive Survey: The paper provides an extensive review of the enabling technologies critical to advancing FSO communication systems, including AO, modulation schemes, and error correction codes. This thorough examination aids in understanding how these technologies address the unique challenges of FSO links.
(ii) Identification of Challenges: It identifies and discusses significant challenges that hinder the deployment of FSO systems, such as atmospheric turbulence, weather-induced signal degradation, and alignment issues. This highlights the need for targeted solutions and informs future research directions.
(iii) Integration of Hybrid Solutions: The paper explores the integration of FSO with radio frequency, millimeter-wave, and Terahertz technologies, emphasizing how hybrid approaches can enhance reliability and coverage in next-generation networks.
(iv) Emerging Trends: The research highlights emerging trends in FSO communication, particularly the integration of artificial intelligence and machine learning techniques. This focus on AI-driven optimization provides insights into how these technologies can enhance the performance of FSO systems in dynamic environments.
(v) Assessment of Future Prospects: The paper evaluates the potential of FSO to meet the increasing demands for high-speed, low-latency communication in the context of 5G and beyond. It provides a forward-looking perspective on scalability, reliability, and deployment strategies that can support the evolution of FSO communication systems.
(vi) Research Directions: Finally, the paper identifies open challenges and outlines future research directions critical to maximizing the effectiveness of FSO technology. This contributes to the broader understanding of the role FSO can play in the future of wireless communication systems.

### 1.4. Article Structure

**Figure 3:**
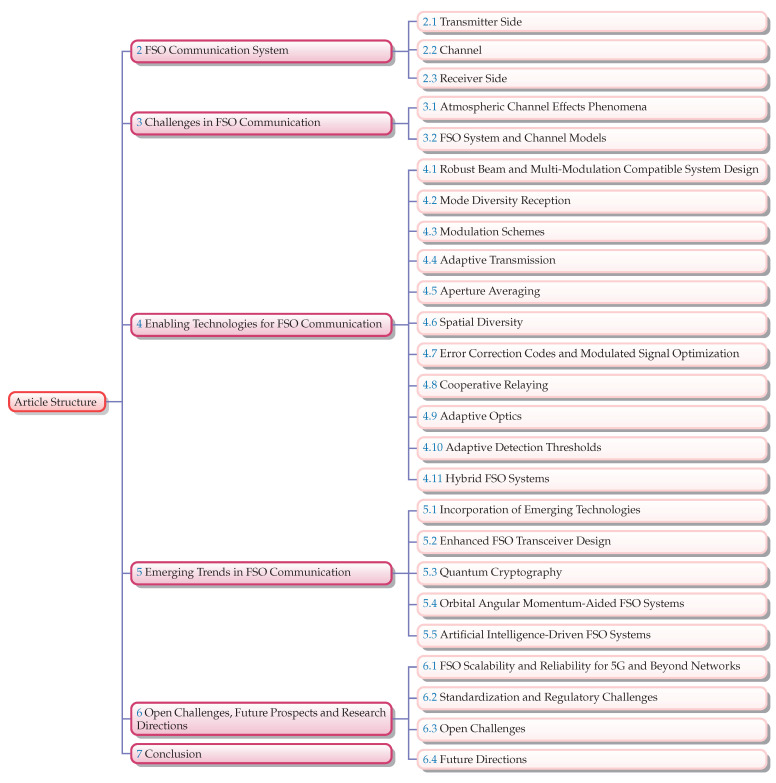
Outline of the article structure.

The paper is organized into sections to provide a comprehensive overview of FSO communication systems in the context of 5G and beyond. Having introduced the background and motivation along with the objectives and scope of the paper, Section 2 delves into the fundamentals of FSO communication systems, detailing their components and performance metrics. Section 3 addresses the various challenges that FSO systems face, including atmospheric turbulence, weather-induced signal degradation, and alignment issues. Section 4 explores the enabling technologies crucial for advancing FSO communication, such as AO, modulation schemes, and error correction codes, as well as the integration of hybrid systems. Section 5 highlights emerging trends, including the use of artificial intelligence and machine learning to optimize system performance. Section 6 discusses the open challenges and future prospects, providing insights into potential research directions and long-term applications. Finally, Section 7 concludes the paper by summarizing the key findings and emphasizing the importance of continued innovation in the field. The structure and organization of the manuscript adhere to the framework illustrated in Figure 3. Each section is meticulously arranged to follow the sequence and logic depicted in the figure, ensuring a coherent and comprehensive presentation of the content.

## 2. FSO Communication System

**Figure 4:**
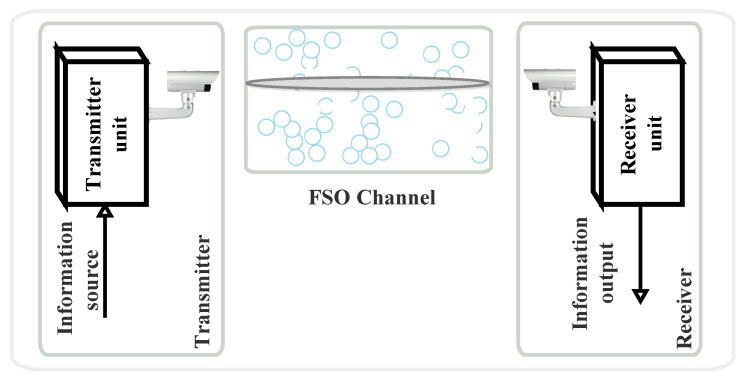
A typical block diagram of an FSO system.

FSO communication systems are a vital part of modern telecommunication technologies, offering a LoS data transmission method through the atmosphere [16] and space telecommunications [17]. As illustrated in Figure 4, an FSO system primarily consists of three fundamental components: the transmitter (Tx-source), the channel, and the receiver (Rx-destination). Each of these components plays a critical role in ensuring the successful transmission of optical signals across free space. For instance, information waveforms are generated by a source and then modulated onto an optical carrier. This optical signal is transmitted through the atmosphere, or free-space, towards a remote destination. Along the way, the optical power emitted by the Tx is influenced by various factors before it reaches the Rx. As a result, the information received might not be an exact copy of the originally transmitted information. At the Rx, the optical signal is collected and converted into an electrical current by a photodetector. The Rx then processes this electrical current to retrieve the originally transmitted information [1,3]. So, the FSO communication system’s efficiency and performance hinge on the seamless integration and alignment of these three components. The Tx initiates the communication by sending out the optical signal, the channel serves as the medium through which the signal travels, and the Rx completes the process by decoding the transmitted information at the destination. This section outlines the various processes involved in transmitting signals from the source to the destination in an FSO communication system.

### 2.1. Transmitter Side

The Tx is responsible for generating and encoding the optical signal that carries the data. It typically comprises a light source, such as a laser diode (LD) or Light-Emitting Diode (LED), which converts electrical data signals into optical signals. The Tx also includes optics to focus and direct the light beam towards the Rx. The FSO Tx operates by encoding and modulating data, amplifying the modulated signal if necessary, and then precisely directing the laser beam through beam-forming optics for transmission across the optical channel.

**Figure 5:**
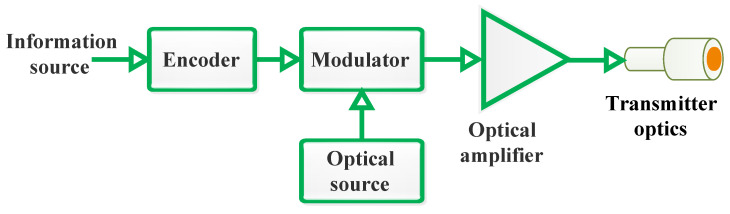
A typical block diagram of an FSO Tx.

Figure 5 provides a depiction of a typical FSO Tx. This system comprises several essential components: an optical source, a modulator (in case of external modulation), an optical amplifier (used as needed), and beam-forming optics. Initially, the data bits generated from the information source undergo channel coding, an optional step performed before the modulation process. This coding step enhances the reliability and performance of the communication by adding redundancy to the data. Following this, the encoded data are modulated onto an optical carrier generated by the optical source. This modulated signal is a laser beam that carries the information through the optical channel. To ensure the signal’s strength and maintain the quality of transmission over long distances or through adverse conditions, the modulated laser beam may be amplified using an optical amplifier. This amplification process boosts the optical intensity of the beam, making it more robust against attenuation and interference. Once the laser beam is sufficiently amplified, it is then passed through the beam-forming optics. These optics play a critical role in collecting the amplified light and refocusing it into a precise and well-collimated beam. This process ensures that the light beam maintains its integrity and alignment as it travels through free space to reach the Rx [1,3,18].

#### 2.1.1. Transmission Wavelength

FSO systems are predominantly designed to function in the near-infrared (near-IR) spectrum, specifically within the 750 to 1600 nm wavelength range. While the clear atmosphere is generally highly transparent to near-IR wavelengths, certain atmospheric molecules can cause significant absorption at specific wavelengths within this range. Also, there are particular wavelength windows around 850 nm, 1060 nm, 1250 nm, and 1550 nm, where the atmospheric attenuation is notably low, measuring less than 0.2 dB/km. Notably, the 850 nm and 1550 nm wavelengths align with the standard transmission windows used in fiber optic communication systems [16]. This compatibility allows FSO systems to leverage existing fiber optic components, which are readily available on the market, thereby reducing costs and improving system integration [1,3,18,19,20].

In addition to near-IR wavelengths, recent research has explored other spectral regions for FSO applications. Wavelengths around 10 μm have been identified for their superior performance in foggy conditions, offering better transmission characteristics. Ultraviolet (UV) wavelengths have also been considered due to their robustness against pointing errors (PEs) and beam blockages. Furthermore, UV wavelengths exhibit lower sensitivity to solar and other background interferences, making them a promising alternative for certain FSO applications [1,21,22]. A detailed discussion of these promising wavelengths is provided in Section 5.2.

#### 2.1.2. Transmission Safety

In FSO communication systems, the primary optical source is typically a semiconductor LD. However, some manufacturers opt for high-power LEDs equipped with beam collimators as an alternative. The chosen optical source must be capable of delivering relatively high optical power across a wide range of temperatures. Additionally, it should feature a long mean time between failures (MTBF), ensuring reliable performance over extended periods. The components associated with the optical source should also have a small physical footprint and consume minimal power [1,3,18]. To meet these stringent requirements, Vertical-Cavity Surface-Emitting Lasers (VCSELs) are predominantly used for operations around the 850 nm wavelength. For operations at the 1550 nm wavelength, Fabry–Pérot (FP) and Distributed Feedback (DFB) lasers are commonly employed. These types of lasers are selected for their efficiency, reliability, and suitability for the specified wavelengths, ensuring optimal performance of the FSO systems [1,18].

Safety considerations are critical when dealing with laser Txs, with the primary concern being the potential risk to the eyes from laser exposure. To mitigate these risks, various safety standards have been established that regulate the transmitted optical power of lasers. These standards take into account factors such as the intensity of the laser beam, its wavelength, proximity to the laser source, peak power in a pulse, and the average power over extended periods [1,3,18,23].

Lasers emit light at different wavelengths, and only certain wavelengths, particularly those in the near-IR (between approximately 400 nm and 1550 nm), have the potential to penetrate the eye with enough energy to harm the retina. Light outside this range is generally absorbed by the cornea and lens before reaching the retina, making it less likely to cause damage. The eye’s tolerance to laser power varies with wavelength, primarily due to the differential absorption of light by water, which is a major component of the eye. Specifically, the absorption coefficient at the front of the eye is significantly higher for longer wavelengths (greater than 1400 nm) compared to shorter wavelengths. Consequently, lasers operating at 1550 nm can have a higher allowable transmission power, which makes them suitable for long-distance applications [1,3,18,19,23].

Additionally, the risk associated with laser exposure differs between high-power pulses and low-power continuous beams. A single high-power laser pulse, even if it lasts less than a microsecond, can cause irreversible damage if it directly enters the eye. In contrast, lower-power continuous beams are hazardous primarily with prolonged exposure. Furthermore, the risk of eye damage diminishes with increased distance from the laser source, as the power density of the laser beam decreases with distance [23]. So, when selecting the operating wavelength for FSO, it is crucial to consider eye-safety regulations and standards. This means that the chosen wavelength must ensure that the optical signals used in the FSO system do not pose a risk to human eyes, either through direct exposure or accidental reflections. Compliance with eye-safety requirements helps prevent potential health hazards and ensures the safe deployment and operation of the FSO system in various environments [21].

### 2.2. Channel

In the FSO communication system, the channel is defined as the free space or atmospheric pathway through which the optical signal is transmitted. Unlike conventional fiber optic systems that use physical cables to guide the light signals, FSO systems depend on a direct LoS between the transmitting and receiving devices. This LoS requirement makes the FSO channel highly susceptible to various environmental influences.

The quality and reliability of the FSO channel can be significantly impacted by several external factors. For terrestrial applications, weather conditions such as fog, rain, and snow can cause attenuation of the signal, reducing its strength as it travels through the atmosphere. Additionally, AT, which includes variations in air density and temperature, can cause the light beam to scatter, further degrading signal quality. Physical obstacles that obstruct the LoS can also interrupt the signal transmission. These environmental factors pose considerable challenges to the stability and performance of FSO communication systems. A comprehensive discussion on these challenges, including detailed analyses of how different conditions affect the FSO channel, is provided in Section 3. The section elaborates on the mechanisms of signal degradation and the strategies employed to mitigate these adverse effects, ensuring more reliable FSO communication.

### 2.3. Receiver Side

The role of the Rx is to capture the transmitted optical signal and convert it back into an electrical signal. It typically includes a photodetector, which senses the incoming light, and various electronic components to amplify and process the received signal. The Rx must be aligned precisely with the Tx to ensure accurate signal detection and minimize data loss. FSO systems are generally classified into two main types based on their detection mechanisms: non-coherent and coherent systems. Although coherent systems typically offer enhanced performance by effectively rejecting background noise, reducing the effects of AT, and achieving higher sensitivity at the Rx, Intensity Modulation with Direct Detection (IM/DD) systems are more prevalent in terrestrial FSO applications. This is due to their simpler design and lower cost. This section will delve into the details of both IM/DD and coherent FSO systems. Also, alternative solutions to conventional coherent Rxs are presented, involving simpler transmission schemes based on direct detection (DD) systems.

#### 2.3.1. Non-Coherent FSO Systems

**Figure 6:**
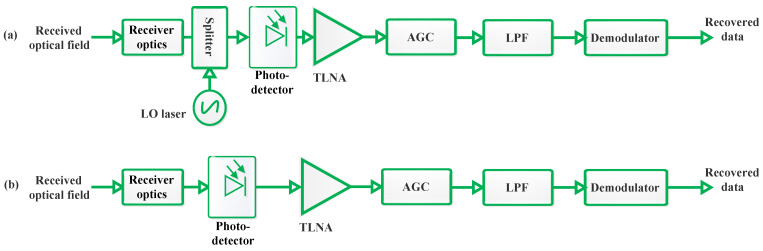
Block diagram of (**a**) coherent and (**b**) IM/DD FSO Rxs. TLNA: Trans-Impedance Low-Noise Amplifier, AGC: Automatic Gain Control, LPF: Low-Pass Filter.

Non-coherent FSO systems, also known as IM/DD systems, rely on the intensity of light to transmit information. At the Rx, changes in light intensity are directly detected by a photodetector without the need for a local oscillator (LO) to mix the optical fields. The Rx front-end in IM/DD FSO systems, depicted in Figure 6b, comprises optical filters and a lens. The primary function of the lens is to gather and focus the incoming light beam onto the photodiode (PD). Commercial FSO systems predominantly use solid-state PDs made from materials such as Indium Gallium Arsenide (InGaAs), Silicon (Si), or Germanium (Ge). These materials are chosen due to their high quantum efficiency and their sensitivity to the commonly utilized wavelengths in FSO systems. Furthermore, these PDs possess very short transit times, resulting in high bandwidth and fast response times, making them ideal for FSO applications [1,18].

Solid-state PDs can either be Positive–Intrinsic–Negative (PIN) diodes or avalanche PDs (APDs), each offering distinct advantages and disadvantages. PIN diodes are known for their simple structure and reliability, while APDs provide internal gain, enhancing their sensitivity but also introducing higher noise and requiring higher operating voltages. The current output from the PD is converted into a voltage using a Trans-Impedance Amplifier (TIA), typically a low-noise Operational Amplifier (Op-Amp) with a load resistor. The selection of this load resistor is crucial and depends on factors such as the transmission rate, the dynamic range of the converted electrical signal, the thermal noise generated by the Rx, and the need to match impedance with other components in the Rx. Finally, the voltage signal from the TIA is passed through a Low-Pass Filter to reduce thermal and background noise, ensuring a cleaner signal for further processing [1].

#### 2.3.2. Coherent FSO Systems

Commercial FSO communication systems traditionally use IM/DD techniques due to their lower implementation complexity and cost effectiveness [24]. However, there is a growing shift towards coherent FSO systems. This trend is driven by recent advancements in the fabrication of integrated coherent Rxs and high-speed digital signal processing (DSP) integrated circuits, which have significantly enhanced the practicality of coherent detection for wired and wireless communications. Fortunately, the suitable bands for FSO transmission coincide with the wavelengths used in fiber optics, specifically the C band, which lowers the cost of optical and optoelectronic components due to mass production [25,26,27].

In coherent FSO systems, information is encoded using modulation techniques such as amplitude and phase modulation. When the signal reaches the Rx, it is optically combined with a locally generated reference signal (LO, optical oscillator, usually a laser with the same wavelength of the received optical signal). This mixing process is crucial for detecting and decoding the information carried by the signal [25,26,27].

Coherent detection in FSO systems retrieves the complete electric field of a signal by measuring both of its field quadratures (i.e., its magnitude and phase) in two polarizations. This approach maximizes the degrees of freedom (DoF) available in the optical channel for carrying information. At the Rx, the incoming signal beam is combined with a LO beam, which provides a phase reference for downconversion as illustrated in Figure 6a. This mixing process with the LO amplifies the received signal, making the detection process primarily limited by shot noise. As a result, coherent detection is often viewed as a method for enhancing Rx sensitivity in FSO systems. Furthermore, coherent detection is effective in rejecting background noise, thermal noise of the TIA and intentional interferences [28].

An intriguing aspect of coherent systems is the ability to send information via the phase, amplitude, or polarization of the optical field, significantly increasing the system’s spectral efficiency (SE). Typical modulation schemes in coherent systems include multilevel Phase-Shift Keying (PSK), Quadrature Amplitude Modulation (QAM), and multilevel Polarization Shift Keying (POLSK). These advanced modulation techniques contribute to the robust and efficient performance of coherent FSO communication systems [1,27].

In the realm of coherent Rxs, there exist two primary methodologies for signal detection: homodyne detection and heterodyne detection. Homodyne detection is known for its superior detection sensitivity, making it highly effective in accurately capturing weak signals. However, this approach necessitates the use of an exceptionally precise optical phase-locked loop (PLL). The implementation of such a highly accurate PLL is technologically demanding and incurs significant costs, rendering it economically challenging to realize on a large scale [27]. As a result of these constraints, heterodyne detection has garnered more widespread attention and consideration in the academic and research literature. While heterodyne detection may not match the sensitivity levels of homodyne detection, it offers a more practical and cost-effective solution. This is because heterodyne systems do not require the same degree of precision in their PLLs, thereby reducing both complexity and expense. Consequently, researchers and engineers have favored heterodyne detection in many applications where the balance of performance, feasibility, and cost is a critical consideration [1,27].

Moreover, the application of DSP techniques to coherent receivers has facilitated the transmission of spectrally efficient advanced modulation formats and effective compensation for impairments. A significant challenge in coherent detection is the synchronization of the optical carrier. To circumvent the need for phase-locked-loop systems, homodyne phase-diversity architectures can operate with free-running (unsynchronized) LO lasers. This approach addresses synchronization issues and additional impairments through the further processing of in-phase (I) and quadrature (Q) baseband signals. This receiver concept, known as the “Intradyne Receiver”, presents a viable alternative. In this phase-diversity scheme, it is crucial for the optical frequencies of the Tx and LO lasers to remain as close as possible to avoid detection penalties [29,30,31]. Frequency offset and phase noise compensation are managed by DSP after the optical front-end in this scheme [31]. Recently, this concept has garnered renewed interest, as advancements in digital signal processors and analog-to-digital converters (ADCs) are achieving speeds that make the hardware implementation of such receivers feasible for higher data rates, such as 10 GBaud [32].

#### 2.3.3. Self-Coherent FSO Systems

Coherent optical transmission systems facilitate efficient information encoding, resulting in improved SE and extended network reach for both digital and analog channels. These systems are especially well suited for medium-to-long-reach links. However, the practical application of full-coherent FSO systems may be limited due to their high power consumption, large physical footprint, and elevated system costs, primarily driven by optical components such as $90^{\circ }$ optical hybrids, LO laser, and high-speed DSP [24,26,28,33]. To tackle these challenges, several quasi-coherent approaches with reduced complexity have been proposed to strike an optimal balance between achievable bit-rate and hardware complexity. In this context, various high-capacity DD transmission techniques, which offer notable advantages such as a a smaller footprint, lower power consumption, and significantly reduced costs, have been extensively explored. Despite these benefits, DD systems are susceptible to linear propagation impairments like chromatic dispersion. These include single-polarization intensity modulation (IM) using high-order Pulse-Amplitude Modulation (PAM), such as PAM-4, and discrete multi-tone (DMT) [34,35,36,37,38,39]. Other techniques under investigation include Stokes vector transceivers [40,41,42], and Single-Sideband (SSB) DD. Notably, the SSB DD scheme employs self-coherent detection, which can be realized through Signal–Signal Beat Interference (SSBI) cancellation or Kramers–Kronig (KK) field reconstruction. Among the various DD-based transmission systems, the SSB DD scheme stands out for its superior performance in terms of network reach [25,26,28,43,44,45].

Moreover, achieving optimal performance in DD systems necessitates intricate manipulation of the optical field at both the Tx and Rx ends. For example, SSB techniques have been investigated to capture amplitude and phase information using DD. However, SSB transmission schemes often suffer from inherent signal-to-signal beating noise (SSBN) in DD systems. Although a Stokes vector Rx could potentially address this issue, its practical use is hindered by sensitivity degradation and increased hardware complexity. A more promising and practical solution for SSB DD transmission is the self-coherent, minimum phase signal-based KK field reconstruction algorithm, which significantly reduces SSBN and enhances system performance [26].

The KK algorithm allows the employment of a single PD and ADC per polarization, while maintaining compatibility with phase-preserving Rx-side post-processing and supporting advanced modulation formats. Also, it enables the reconstruction of the complex-valued electric field at the Rx using only intensity measurements, allowing for digital compensation of propagation effects similar to those achieved with more complex coherent Rxs [26,43]. Despite the significant complexity reduction offered by the self-coherent-based KK FSO (KK-FSO) system, several challenges remain. For example, the nonlinear operations (logarithmic and exponential) in the KK method require the DSP to operate at much higher speeds than the Nyquist sampling rate to handle spectral broadening. A potential solution to reduce the sampling rate is to bypass these nonlinear operations. For instance, an upsampling-free KK method employs a Taylor expansion approximation for these operations. However, these approximations generally require a higher carrier-to-signal power ratio (CSPR), leading to a sensitivity penalty. To meet the demands for an increased tone power and high sampling rate of both the traditional KK and the upsampling-free KK methods, a DC-Value (DC-V) method can be used [26,27,33]. This method leverages the SSB and DC-V properties of the minimum phase signal, offering an upsampling-free phase reconstruction process with low tone power operation.

Additionally, a significant challenge in the KK implementation is the necessity for a mandatory carrier tone at the edge of the information spectrum to mitigate the effects of SSBN [27]. This carrier tone can be introduced in the optical domain, RF domain, or digital domain. Both optical and RF methods, however, increase the hardware requirements and complexity. Thus, inserting the virtual carrier in the digital domain is a simpler alternative that avoids these additional resource demands. However, this approach increases the signal’s dynamic range, which leads to a quantization noise penalty owing to the finite resolution of the Digital-to-Analog Converter (DAC) [26,27,43].

A digital resolution enhancer (DRE) method has been introduced to mitigate the impacts of quantization noise in low-resolution DACs. Unlike traditional rounding-off quantizers, the DRE employs a dynamic quantization method that efficiently minimizes in-band quantization noise and enhances transmission performance. Building on this DRE technique, a novel self-coherent FSO scheme has been proposed, further simplifying the KK-FSO system. In this new system, the optical carrier is substituted with a virtual carrier, which is directly incorporated into the signal within the digital domain. This approach eliminates the need for additional hardware to generate and synchronize the optical carrier. Additionally, the DRE method allows the use of extremely low-resolution DACs with just three physical numbers of bits, leading to significant reductions in both power consumption and cost. On the Rx side, the KK transform is replaced with the DC-V method, enhancing power efficiency by lowering the requirements for oversampling and CSPR [33]. Key distinctions among non-coherent (IM/DD), coherent, and self-coherent FSO systems are presented in Table 1. Also, a thorough analysis of optical transceivers is available in [27].

## 3. Challenges in FSO Communication

Following the introduction of fundamental concepts in FSO communication systems, this section explores the challenges primarily arising from atmospheric channel effects. Subsequently, we discuss various FSO systems and channel models that can be utilized to assess performance.

### 3.1. Atmospheric Channel Effects Phenomena

FSO communication is a promising technology with notable benefits, yet it encounters several hurdles that impede its broad implementation. This section examines the primary challenges in FSO communication, such as atmospheric interference, alignment and pointing precision, range restrictions, and security issues. Addressing and overcoming these obstacles is essential for creating robust FSO communication systems that deliver reliable and efficient wireless communication across diverse applications.

#### 3.1.1. Atmospheric Interference

Atmospheric interference poses a major challenge to FSO communication. Various atmospheric conditions, such as weather, aerosols, and particles, can impact the propagation of optical signals. For instance, FSO communication is heavily influenced by weather conditions such as snow, rain, sleet, fog, and haze. These atmospheric phenomena can attenuate, scatter, or absorb optical signals, resulting in significant signal attenuation and weakened signal strength, which degrade performance. Additionally, aerosols and particles in the atmosphere greatly affect FSO signal propagation. These tiny solid or liquid particles can scatter and absorb the optical beams used in FSO communication, causing signal degradation [1,7,20]. The transmit power can be increased to compensate for these losses, but the maximum permissible power is constrained by eye-safety standards [2,20]. To counteract the negative impacts of weather on FSO communication, several strategies such as AO, wavelength selection, hybrid FSO systems, air quality monitoring, redundancy, and diversity techniques can be employed. Further details on atmospheric attenuation models are presented in Section 3.2.

#### 3.1.2. Atmospheric Turbulence

AT arises from irregularities in air temperature, humidity, and pressure, which cause random variations in the atmosphere’s refractive index. This results in turbulent eddies of different sizes and strengths, presenting significant challenges for the propagation of FSO signals. Such turbulence leads to detrimental effects like beam wander, beam spreading, scintillation, and phase distortion, all of which can degrade FSO communication system performance [2,15,20]. To develop reliable and efficient FSO systems, it is essential to understand and mitigate these effects. Promising solutions include advances in AO, diversity techniques, ECC, and beam steering technologies. Section 4 provides further details on these mitigation strategies.

Several mathematical models are developed and analyzed to accurately characterize the probability density function (PDF) of random irradiance fluctuations under various turbulent conditions, such as weak, moderate, strong, and saturation. Examples of these models include the log-normal (LN), Gamma–Gamma (ΓΓ), *K*-distribution, and negative exponential (NE) models [15,46,47]. Section 3.2 discusses several AT models for the FSO system.

#### 3.1.3. Alignment and Pointing Accuracy

In FSO communication, maintaining accurate alignment and pointing between the Tx and Rx is crucial. Misalignments, which can occur along both vertical and horizontal axes, are often modeled using Hoyt and Rayleigh distributions [8]. Such misalignments can cause PE that diminish the performance of the FSO system. These errors are quantified as the Euclidean distance between the centers of the photodetector and the beam footprint at the Rx. In addition to causing signal degradation, PE can lead to intermittent or complete signal loss. These errors may arise from various factors, including the movement of mobile stations, platform vibrations, building sway, transceiver sway, seismic activity, inaccuracies in the tracking system, or stress in the system’s mechanical or electronic components. An alternative form of PE, known as beam wandering, is due to AT, where large-scale atmospheric eddies cause the transmitted beam to deviate from its intended trajectory [2,7].

Dynamic environments pose considerable challenges for the propagation and stability of optical signals. These environments are marked by ongoing changes in position, orientation, or atmospheric conditions, which can affect the alignment, signal integrity, and overall performance of FSO systems. Therefore, in mobile applications, continuous realignment is essential to sustain a stable connection, necessitating advanced tracking and control systems. A thorough review of pointing, acquisition, and tracking (PAT) mechanisms employed in FSO communication systems is provided in [7].

#### 3.1.4. Reliability and Security Concerns

FSO communication inherently benefits from enhanced security due to its highly directional and narrow optical beams, making interception challenging without direct physical access to the LoS path. However, security concerns in FSO communication, such as eavesdropping, jamming, and physical obstruction, can significantly impact the propagation and reliability of optical signals [2]. These challenges must be addressed through a combination of advanced encryption, AO, dynamic beam management, and robust system design. By mitigating these security risks, FSO communication can offer a secure and efficient alternative to traditional RF-based systems, particularly in applications where high data rates and secure transmission are paramount. This section provides a detailed discussion of these FSO security concerns.

##### Eavesdropping

Similar to wired links, wireless optical transmission is inherently more secure than RF transmission due to the high directionality of optical beams as opposed to the almost broadcast nature of RF signals, making them significantly harder to intercept, though not impossible [48]. However, there are scenarios where the threat of eavesdropping from one or more malicious actors may arise. Eavesdropping in FSO communication involves intercepting the optical beam to gain unauthorized access to the transmitted data. This can happen because of AT, optical beam divergence, channel scattering, and PE. Consequently, adversaries might access information through scattering over a non-LoS (NLoS) channel and scattering over a LoS channel caused by aerosol particles. Eavesdropping may be feasible if both the legitimate and the adversary Rxs are positioned close to each other within the FSO links [48,49,50]. Additionally, eavesdropping is possible when part of the beam radiation is reflected by small particles and detected by an external observer not in the LoS of either communication peer. However, the power received by the eavesdropper in such cases will be significantly less than in an equivalent RF scenario. An alternative scenario involves the eavesdropper blocking the laser beam to collect more power. In this case, the legitimate Rx would notice a significant decrease in the average received power, prompting the communication to be stopped for security reasons [48].

Traditionally, upper-layer cryptographic algorithms, which assume a limited computational capability of eavesdroppers, have been sufficient to protect confidential messages. However, due to the complexity of key generation and allocation, along with the current advanced hardware available to eavesdroppers, these cryptographic algorithms may not meet future wireless network security requirements. In addition to these upper-layer algorithms, physical (PHY) layer key generation and encryption methods, which exploit channel reciprocity to generate key packets [51], and PHY layer wiretap coding, which leverages the difference in channel quality between the Tx-to-Rx and Tx-to-eavesdropper channels to protect confidential messages [52,53], can be employed to secure confidential messages in the FSO-RF relay network [50].

The challenge of ensuring secure communications in the presence of external eavesdroppers is a well-known issue in digital communications. There is increasing interest in the PHY layer secrecy performance over fading channels, leading the research community to focus on PHY layer security (PLS) as a means to avoid eavesdropping without relying on complex encryption techniques in the upper layers [54]. Consequently, the PLS of FSO links has been studied over M [54,55,56] and ΓΓ turbulence channels [48]. Additionally, ref. [57] explores the PLS of a LoS FSO link employing Orbital Angular Momentum (OAM) multiplexing. The information security risks of an FSO communication system with an eavesdropper outside the laser beam, who could obtain information through a non-LoS scattering channel, are examined in [58]. Furthermore, key PLS metrics such as secrecy outage probability (SOP), average secrecy capacity (ASC), and the probability of strictly positive secrecy capacity (SPSC) are unified in the context of FSO communication in [59,60,61].

In [61,62,63], the secrecy of a mixed RF-FSO system is analyzed, where an adversary intercepts secure information via the FSO link by capturing a fraction of the optical power due to beam scattering. The findings in [62], supported by simulations, demonstrate that factors such as photo-aperture size, fading parameters, and the average eavesdropper signal-to-noise ratio (SNR) are crucial for securing RF-FSO communications. Specifically, the results indicate that under most weather conditions, and with a photo-aperture size of approximately 200 mm, mixed RF-FSO relaying is advantageous for designing a secure RF-FSO relay network. Furthermore, refs. [59,64] propose a hybrid RF/FSO system utilizing RIS reflection to interfere with eavesdroppers in FSO eavesdropping scenarios. The system’s PLS performance is also analyzed and optimized.

The performance of PLS in mixed FSO-RF systems has been the focus of several studies. In [50], a PLS secrecy analysis for a mixed FSO-RF system under a ΓΓ and Rayleigh fading scenario is conducted. The study in [65] analyzes the secrecy performance of a mixed FSO-RF simultaneous wireless information and power transfer (SWIPT) system using a decode-and-forward (DF) relaying scheme. These studies consider eavesdropping on the FSO side with full channel state information (CSI) of the eavesdropper’s channel. However, the studies reported in [54,63,65,66,67,68,69,70] are limited to PLS secrecy analysis under the assumption of ideal power amplifier (PA) deployment at the RF front-end. In practice, the non-negligible power consumption by PAs significantly impacts the secrecy performance, making the effects of PA efficiency crucial in PLS secrecy analysis. Although a few studies have examined the impact of hardware imperfections on mixed RF-FSO systems’ performance, such as [71,72], only a limited number have done so.

To model these hardware imperfections, ref. [72] considers soft envelope limiter (SEL) PA nonlinearities, while [71] uses a degradation PA model to characterize the hardware impairments. The study in [73] analyzes the secrecy performance of a hybrid RF-FSO system under hardware impairments using the degradation PA model, focusing on scenarios where only the RF eavesdropper intercepts secure information. Additionally, ref. [49] propose a different system structure for a mixed FSO-RF system under hardware imperfections, where all RF Rxs use the SWIPT technique to collect energy from the relay’s wireless signals. This study also assumes that both FSO- and RF-side eavesdroppers can overhear the intended information separately and simultaneously.

##### Jamming

The wireless medium’s inherent openness renders it vulnerable to both intentional and unintentional interference. A significant source of unintentional interference is interference from neighboring cells. Conversely, intentional interference, like jamming attacks, represents adversarial actions targeting a victim Rx [74]. Therefore, as wireless links, the reliability and security of the entire FSO system are susceptible to intentional jamming. This threat is particularly significant in military platforms or other security-sensitive communication protocols controlled by FSO networks, which can be targeted and disrupted by adversaries [75]. The impact of jamming on RF systems, including wireless multihop networks, has been extensively studied from various perspectives [74,76,77,78,79]. FSO systems are also vulnerable to jamming because the spectral width of the laser light in FSO communications is typically confined to specific wavelengths to ensure compatibility with optical fiber communication. Usually, FSO communication operates within very narrow wavelength ranges, such as 1300 nm or 1550 nm. Consequently, the operating frequency of a legitimate user link is readily accessible in FSO systems, making them highly susceptible to jamming attacks [75].

**Figure 7:**
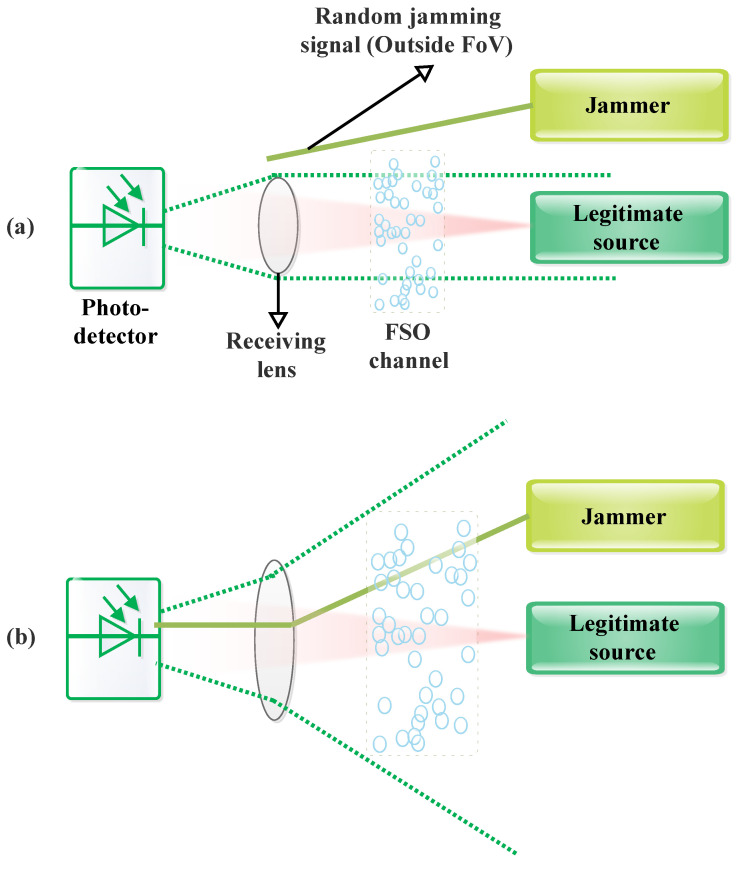
FSO detector with (**a**) a narrow FoV and (**b**) a wide FoV.

Additionally, in an FSO system, it is crucial to optimize the field-of-view (FoV) based on the specific application. The FoV is defined as the solid angle through which a detector can receive an incoming optical signal. As illustrated in Figure 7a, the optical signal emitted by a jammer is captured when it falls within the FoV; otherwise, it passes through unobstructed. Typically, the FoV of the receiving aperture is kept wide to maximize signal reception, mitigate the effects of fading due to AT and PE, address aperture misalignment issues, and prevent communication link failures caused by obstacles in the FSO system [75].

In tracking-based FSO communication systems, a wide FoV of up to $60^{\circ }$ is employed to increase the tracking range and reduce optical aberrations [80]. Additionally, coarse PAT (CPAT) systems consider FoVs up to 70° [7,81]. Due to the inherent fluctuations of UAVs, the optimal FoVs for UAV-based FSO links are significantly larger than those for ground-based FSO links [82].

Moreover, aperture averaging is an essential technique in FSO systems, as the unpredictable nature of the received signal caused by fading imposes severe constraints on system performance. Aperture averaging is a technique used to control and define the size of the detector or receiving aperture needed to capture the traversing optical field as effectively as possible. This process is particularly crucial for FSO systems operating in strong and saturated regimes. Aperture averaging helps to mitigate signal scintillation or irradiance flux variations (reducing fade probability), minimize the impacts of angle of arrival (AoA) fluctuations and boresight angle, and optimize the average signal power at the detector plane. Additionally, it serves as a primary method to ensure spatial diversity [46,83]. A comprehensive discussion on aperture averaging is provided in Section 4.9. Therefore, to maintain effective FSO communication through the continuous establishment of transmission links, it is crucial to have a larger aperture diameter, which consequently results in a wide FoV [46,82].

A narrow FoV restricts the operational coverage area, making it less ideal for FSO systems. Conversely, a wider FoV increases ambient light background noise, which can be mitigated through various methods to capture a larger portion of the incoming optical beam at the FSO Rx [82,84]. Also, a higher FoV offers a broader coverage area and improved signal reception. For instance, a larger aperture diameter (8 inches) significantly reduces error probability in comparison with a smaller aperture diameter (2 inches) [46].

Moreover, implementing a higher FoV does not necessitate additional circuitry for pointing and tracking mechanisms, thereby guaranteeing reliable wireless connectivity between the transmitting and receiving apertures. As a result, FSO systems with a higher FoV are less susceptible to misalignment errors, which helps to mitigate the blocking probability caused by obstacles between the source and destination points. This reduces the need for stringent alignment control mechanisms. However, the extensive FoV essential for effective signal reception, coupled with the multiple receiving nodes required in cooperative networks, renders FSO links susceptible to jamming attacks at any node. The detector can easily capture the jamming signal, as illustrated in Figure 7b, creating a significant challenge in differentiating the transmitted signal from unwanted jamming signals [46,82]. Moreover, in a dual-hop link, an attack that blocks either node will result in the failure of the entire source–relay–destination link.

Jamming in point-to-point (p2p) FSO communication is studied in [75,85], while a similar scenario for cooperative relaying is explored in [86,87]. In [75], the impact of jamming, where the jammer aims to congest the Rx with unwanted noise, is investigated for FSO communication systems to analyze the outage probability (OP) and bit-error rate (BER) expressions of the system. However, none of these studies address the issue of jamming in the context of aperture averaging and phase error impairment in Exponentiated Weibull (EW) statistics. In [75], it is demonstrated that jamming can severely degrade the performance of single-input single-output (SISO) FSO systems. In [46], the threat to security-constrained reliable FSO communications due to jamming over an exponentially weighted fading channel is examined, taking into account various AT conditions, aperture averaging, and PE. Analytical expressions for the average BER (ABER) and OP are derived for the FSO system under the influence of a random jammer. In [88], jamming for cooperative relaying is further investigated. To address the impact of jamming in FSO communication networks, a buffer-aided relaying approach is proposed. This method employs a relay node with a finite-size buffer and assumes that both relay and destination nodes are susceptible to jamming. Comparisons with a non-buffer-aided (NBA) cooperative FSO system impacted by jamming show that the proposed buffer-aided FSO system considerably outperforms its NBA counterparts.

### 3.2. FSO System and Channel Models

FSO channel models are essential for understanding and predicting system performance. These models account for various factors that affect the propagation of light, such as AT, absorption, scattering, and weather conditions like fog or rain. Accurate channel modeling helps in designing robust FSO systems, optimizing link availability, and ensuring reliable data transmission. By analyzing different channel models, researchers and engineers can develop strategies to mitigate adverse effects, enhancing the efficiency and reliability of FSO communication systems.

#### 3.2.1. FSO System Model

In this section, we analyze a practical FSO link that employs IM/DD with On–Off Keying (OOK) modulation. The data are encoded onto the instantaneous intensity of an optical beam at the Tx. It is assumed that the optical power emitted from the transmit aperture into free space is affected by factors such as misalignment fading (PE), fading induced by AT, and background or ambient noise before it reaches the receive aperture. These factors lead to variations in signal intensity. As a result, the received electrical signal *r* at the receive aperture can be modeled as follows [3,89,90,91]:

(1) $$r=\eta_{e}hx+n,$$

where $\eta_{e}$ denotes the effective photoelectric conversion ratio of the Rx, x∈{0,1} represents the transmitted information bit, *n* represents the additive white Gaussian noise (AWGN) with zero mean and variance $\sigma_{n}^{2}=N_{0}/2$, $N_{0}$ denotes the one-sided noise power spectral density in watts/Hz, and the term $h=h_{\ell }h_{a}h_{p}$ signifies the irradiance affecting the channel state, which is a product of the deterministic path loss $h_{\ell }$, the random attenuation due to AT-induced fading $h_{a}$, and the random attenuation caused by geometric spread and PE $h_{p}$. Both $h_{a}$ and $h_{p}$ are random variables characterized by their respective PDFs, $f_{h_{a}}\left(h_{a}\right)$ and $f_{h_{p}}\left(h_{p}\right)$.

##### Noise Model

The noise performance of a Rx is influenced by the type of photodetector employed, specifically whether a PIN photodetector or an APD is used. It is important to recognize that each of these photodetectors contributes to the overall noise in distinct ways. In this subsection, we delve into the different noise models associated with both PIN and APD photodetectors, providing a comprehensive review of their respective noise characteristics and how they impact the Rx performance.

If an APD Rx is employed, the overall noise contribution is understood to consist of three primary components: thermal noise, shot noise, and dark current. However, assuming the dark current is insignificant, the noise generated by the APD Rx, denoted as *n*, can be represented as the sum of the thermal noise, $i_{Th}$, and the shot noise, $i_{Sh}$. Therefore, the equation representing the APD Rx noise is [92,93]

(2) $$n=i_{Th}+i_{Sh},$$

where $i_{Sh}$ and $i_{Th}$ represent the shot noise and thermal noise, respectively. The thermal noise $i_{Th}$ is characterized as a zero-mean stationary Gaussian random process, with its variance defined by [92,93]

(3) $$\sigma_{Th}^{2}=4k_{B}\frac{T}{R_{L}}F_{n}\Delta f,$$

where $k_{B}$ represents the Boltzmann constant, *T* denotes the absolute temperature in degrees Kelvin, $R_{L}$ represents the APD load resistance, $F_{n}$ represents the amplifier noise figure, and $\Delta f=R_{b}/2$ denotes the symbol effective noise bandwidth that is associated with the bit rate $R_{b}$.

Shot noise is influenced by the APD Rx and can be modeled similarly to thermal noise using a zero-mean stationary Gaussian random process. The variance in shot noise can be described as

(4) $$\sigma_{\mathrm{Sh}}^{2}=2q_{e}{\bar{g}}^{2}F_{A}ℜhP_{t}m\Delta f,$$

where $q_{e}$ is the electron charge, *m* is the modulation index, $P_{t}$ represents the transmitted optical power, *ℜ* and $\bar{g}$ are the APD responsivity and average gain, respectively, and $F_{A}$ represents the excess noise factor of the APD, which is defined by

(5) $$F_{A}=k_{A}\bar{g}+\left(1-k_{A}\right)\left(2-\frac{1}{\bar{g}}\right),$$

where $k_{A}$ represents the ionization factor. Utilizing the characteristics of Gaussian random variables, the total APD noise can be modeled as a stationary random process with a mean of zero. The aggregate variance can be defined as [92,93]

(6) $$\sigma_{n}^{2}=\sigma_{th}^{2}+\sigma_{Sh}^{2}=2\Delta f\{2k_{B}\frac{T}{R_{L}}F_{n}+q_{e}{\bar{g}}^{2}F_{A}ℜhP_{t}m\}.$$

The instantaneous electrical SNR per symbol, represented by $\gamma_{i}$ at the output of the APD, is thus given by

(7) $$\gamma_{i}=\frac{{\left(\bar{g}ℜhP_{t}\right)}^{2}}{2\Delta f\{2k_{B}\frac{T}{R_{L}}F_{n}+q_{e}{\bar{g}}^{2}F_{A}ℜhP_{t}m\}}.$$

#### 3.2.2. FSO Channel Models

This section provides an overview of various channel models that can be utilized to predict the performance of FSO systems. These models are essential for understanding how different environmental factors and conditions affect the effectiveness and reliability of FSO communications. The models incorporate PDFs derived from turbulence intensity to characterize the impact of turbulence on FSO signals. Analyzing these models allows us to gain insights into how factors like AT, weather conditions, and signal attenuation impact system performance, enabling us to optimize and better anticipate the behavior of FSO systems in real-world scenarios.

##### Atmospheric Attenuation

As an optical beam travels through the atmosphere, it experiences atmospheric loss. The signal power attenuation follows the exponential Beer–Lambert law and is expressed as [3]

(8) $$h_{\ell }\left(\lambda ,z\right)=\frac{P(\lambda ,z)}{P(\lambda ,0)}=\exp\left(-\sigma \left(\lambda \right)z\right)$$

where $h_{\ell }\left(\lambda ,z\right)$ represents the loss as a function of the propagation path length *z* at wavelength λ. The signal power at a distance *z* and the emitted power at z=0 are expressed as P(λ,z) and P(λ,0), respectively. The total extinction coefficient or the attenuation coefficient σ(λ) per unit length is defined by [3]

(9) $$\sigma \left(\lambda \right)=\alpha_{m}\left(\lambda \right)+\alpha_{a}\left(\lambda \right)+\beta_{m}\left(\lambda \right)+\beta_{a}\left(\lambda \right),$$

where $\alpha_{m,a}$ are the absorption coefficients for molecules and aerosols, respectively, and $\beta_{m,a}$ represent the scattering coefficients for molecules and aerosols, respectively.

The attenuation $h_{\ell }$ is considered a constant scaling factor over an extended period, ensuring consistent behavior. Additionally, it depends on the size and distribution of scattering particles, along with the wavelength used. This attenuation can be described regarding visibility, which is directly measurable from the atmosphere. Empirically, attenuation is characterized in relation to visibility as [3]

(10) $$\sigma (\lambda )=\frac{3.912}{V}{\left(\frac{\lambda }{550}\right)}^{-q},$$

where *V* represents visibility (in kilometers), and *q* is a parameter influenced by the particle size distribution in the atmosphere as described by the Kruse model [90]:

(11) $$q=\left\{\begin{array}{cc} 1.6 & V>50\;\;\mathrm{km}\\ 1.3 & 6\;\;\mathrm{km}<V<50\;\;\mathrm{km}\\ 0.585V^{1/3} & V<6\;\;\mathrm{km} \end{array}\right.$$

Additionally, Kim introduces an extended model designed to improve accuracy in low-visibility situations. The Kim model is described as [90]

(12) $$q=\left\{\begin{array}{cc} 1.6\; & V>50\;\mathrm{km}\\ 1.3 & 6\;\;\mathrm{km}<V<50\;\;\mathrm{km}\\ 0.16V+0.34 & 1\;\;\mathrm{km}<V<6\;\;\mathrm{km}\\ V-0.5 & 0.5\;\;\mathrm{km}<V<1\;\;\mathrm{km}\\ 0 & V<0.5\;\;\mathrm{km} \end{array}\right.$$

##### Pointing Error or Misalignment Fading

An FSO link is a LoS communication system that uses a narrow optical beam, which requires precise pointing accuracy to ensure the effective performance and reliability of optical systems [16]. PE and signal degradation at the Rx are typically caused by wind loads and thermal expansions, leading to random movements of the building. Assuming a Gaussian spatial intensity profile characterized by a beam waist $w_{z}$ at the Rx plane—located a distance *z* from the Tx—and a circular aperture with a radius $r_{a}$, the fraction of collected power resulting from geometric spreading with a radial displacement $\alpha_{d}$ from the detector’s origin can be approximated using a Gaussian distribution as [3,89]

(13) $$h_{p}\left(\alpha_{d}\right)\approx A_{0}\exp\left(-\frac{2\alpha_{d}^{2}}{w_{z_{eq}}^{2}}\right),$$

where $w_{z_{eq}}^{2}=w_{z}^{2}\sqrt{\pi }\mathrm{erf}\left(\upsilon \right)/2\upsilon \exp\left(-\upsilon^{2}\right)$, $\upsilon =\sqrt{\pi }r_{a}/\sqrt{2}w_{z}$, $w_{z_{\mathrm{eq}}}$ is the equivalent beamwidth, $A_{0}={\left[\mathrm{erf}\left(\upsilon \right)\right]}^{2}$, and erf(·) is the error function defined as [94]

(14) $$\mathrm{erf}\left(x\right)=\left(2/\sqrt{\pi }\right)\int_{0}^{x}e^{-u^{2}}du.$$

Assuming that the horizontal and elevation sway are independent and follow identical Gaussian distributions, the radial displacement $\alpha_{d}$ will follow a Rayleigh distribution. Consequently, $f_{h_{p}}\left(h_{p}\right)$ can be expressed as

(15) $$f_{h_{p}}\left(h_{p}\right)=\frac{\gamma_{p}^{2}}{A_{0}^{\gamma_{p}^{2}}}h_{p}^{\gamma_{p}^{2}-1},\;0\leq h_{p}\leq A_{0},$$

where $\gamma_{p}=w_{z_{eq}}/2\sigma_{s}$ denotes the ratio of the equivalent beam radius to twice the standard deviation (jitter) of the PE displacement, and $\sigma_{s}^{2}$ represents the jitter variance at the Rx.

##### Atmospheric Turbulence

The intensity fluctuations over the FSO channel have been described using various statistical models in the literature, tailored for different turbulence regimes. The commonly used models encompass the LN distribution, ideal for weak turbulence and frequently applied due to its strong correlation with experimental data, the ΓΓ distribution for moderate to strong turbulence, and the K-distribution for strong turbulence. These models incorporate PDFs based on turbulence intensity to characterize the impact of turbulence on FSO signals. Other widely used models include the I-K distribution, NE, and M distribution models. However, this work focuses on the LN, ΓΓ, and generic M distribution models.

Log-normal Distribution: Generally, the LN model is only appropriate for weak turbulence conditions and for link ranges shorter than 100 m [95]. Therefore, the intensity fluctuation PDF for weak turbulence, modeled using the LN distribution, is presented by [96]

(16) $$f_{h_{a}}\left(h_{a}\right)=\frac{1}{2h_{a}\sqrt{2\pi \sigma_{x}^{2}}}\exp\left(-\frac{{\left(\ln\left(h_{a}\right)+2\sigma_{x}\right)}^{2}}{8\sigma_{x}^{2}}\right),$$

where $\sigma_{x}^{2}$ denotes the log-amplitude variance specified for plane waves and spherical waves, respectively, and is expressed as [96]

(17a) $$\begin{array}{cc} \sigma_{x}^{2}{|}_{\mathrm{plane}}= & 0.307C_{n}^{2}k^{7/6}L^{11/6}, \end{array}$$

(17b) $$\begin{array}{cc} \sigma_{x}^{2}{|}_{\mathrm{spherical}}= & 0.124C_{n}^{2}k^{7/6}L^{11/6}, \end{array}$$

where *L* denotes the distance, $k=\frac{2\pi }{\lambda }$ denotes the optical wave number, and $C_{n}^{2}$ represents the altitude-dependent index of the refraction structure parameter. Also, with respect to the log-irradiance variance, $\sigma_{x}^{2}=\sigma_{l}^{2}/4$, where $\sigma_{l}^{2}$ is the log-irradiance variance expressed as [96]

(18a) $$\begin{array}{cc} \sigma_{l}^{2}{|}_{\mathrm{plane}}= & 1.23C_{n}^{2}k^{7/6}L^{11/6}, \end{array}$$

(18b) $$\begin{array}{cc} \sigma_{l}^{2}{|}_{\mathrm{spherical}}= & 0.50C_{n}^{2}k^{7/6}L^{11/6}. \end{array}$$

The $C_{n}^{2}$ parameter is crucial for quantifying the refractive index fluctuations in AT. It varies with the wavelength, atmospheric altitude, and temperature. Various $C_{n}^{2}$ profile models have been presented in the literature, with the Hufnagel valley model being the most widely used for altitude and is defined as [6,91,94,96,97]

(19) $$\begin{array}{cc} C_{n}^{2}\left(h_{n}\right) & =0.00594{\left(v_{w}/27\right)}^{2}{\left(10^{-5}h_{n}\right)}^{10}\exp\left(-h_{n}/1000\right)\\ \; & +2.7\times 10^{-16}\exp\left(-h_{n}/1500\right)+\hat{A}\exp\left(-h_{n}/100\right), \end{array}$$

where $h_{n}$ represents the altitude in meters (m) and $\hat{A}$ denotes the nominal value of $C_{n}^{2}\left(0\right)$ at ground level in $m^{-2/3}$. The value of $C_{n}^{2}$ for FSO links at ground level is approximately $1.7\times 10^{-14}\;$ $m^{-2/3}$ during the daytime and $8.4\times 10^{-15}\;$ $m^{-2/3}$ at night. Generally, $C_{n}^{2}$ ranges from $10^{-13}\;$ $m^{-2/3}$ in strong turbulence to $10^{-17}\;$ $m^{-2/3}$ in weak turbulence, with an average typical value of $10^{-15}\;$ $m^{-2/3}$ [94,97]. The variable $v_{w}$ is the root mean square wind speed (pseudo wind) in meters per second (m/s), typically valued at 21 m/s, is given as [94]

(20) $$w={\left[\frac{1}{15\times 10^{3}}\int_{5\times 10^{3}}^{20\times 10^{3}}V^{2}\left(h_{n}\right)dh_{n}\right]}^{1/2},$$

where $V(h_{n})$ is generally characterized by the Bufton wind model as described by [94]

(21) $$V\left(h_{n}\right)=\omega_{s}h_{n}+V_{g}+30\exp\left[-{\left(\frac{h_{n}-9400}{4800}\right)}^{2}\right],$$

where $V_{g}$ denotes the ground wind speed, and $\omega_{s}$ represents the *slew rate*, which is related to the satellite’s movement in relation to the observer on the ground.

Gamma–Gamma Distribution: In many cases, when the LN distribution characterization is invalid due to strong turbulence regimes, the ΓΓ distribution is typically used to model scintillation effects. Furthermore, the ΓΓ model can characterize fading gains across both weak and strong turbulence scenarios. The PDF of $h_{a}$ based on the ΓΓ distribution is defined as [6,91,94,96,97]

(22) $$f_{h_{a}}\left(h_{a}\right)=\frac{2{\left(\alpha \beta \right)}^{(\alpha +\beta )/2}}{\Gamma (\alpha )\Gamma (\beta )}{\left(h_{a}\right)}^{\frac{\left(\alpha +\beta \right)}{2}-1}K_{\alpha -\beta }\left(2\sqrt{\alpha \beta h_{a}}\right),$$

where Γ(·) denotes the gamma function, while $K_{\nu }\left(\cdot \right)$ represents the modified Bessel function of the second kind of order ν. The parameters α and β refer to the effective number of large-scale and small-scale eddies involved in the scattering process, respectively. These parameters are defined for the plane wave as [91,94,96,97]

(23a) $$\begin{array}{cc} \alpha = & {\left[\exp\left(\frac{0.49\sigma_{R}^{2}}{{\left(1+1.11\sigma_{R}^{12/5}\right)}^{7/6}}\right)-1\right]}^{-1}, \end{array}$$

(23b) $$\begin{array}{cc} \beta = & {\left[\exp\left(\frac{0.51\sigma_{R}^{2}}{{\left(1+0.69\sigma_{R}^{12/5}\right)}^{5/6}}\right)-1\right]}^{-1}, \end{array}$$

and for the spherical wave, they can be expressed as [6,91,98]

(24a) $$\begin{array}{cc} \alpha = & {\left[\exp\left(\frac{0.49\sigma_{R}^{2}}{{\left(1+0.18d^{2}+0.56\sigma_{R}^{12/5}\right)}^{7/6}}\right)-1\right]}^{-1}, \end{array}$$

(24b) $$\begin{array}{cc} \beta = & {\left[\exp\left(\frac{0.51\sigma_{R}^{2}{\left(1+0.69\sigma_{R}^{12/5}\right)}^{-5/6}}{{\left(1+0.9d^{2}+0.62d^{2}\sigma_{R}^{12/5}\right)}^{5/6}}\right)-1\right]}^{-1}, \end{array}$$

where $d≜{\left(\frac{kD^{2}}{4L}\right)}^{1/2}$, with *D* denoting the diameter of the Rx aperture, and $\sigma_{R}^{2}$ denoting the Rytov variance, a metric for the strength of turbulence fluctuations. The Rytov variance $\sigma_{R}^{2}$ is expressed for both plane and spherical waves, respectively, as [91,94,97]

(25a) $$\begin{array}{c} \sigma_{R}^{2}{|}_{\mathrm{plane}}=1.23\;C_{n}^{2}\;k^{7/6}\;L^{11/6}, \end{array}$$

(25b) $$\begin{array}{c} \sigma_{R}^{2}{|}_{\mathrm{spherical}}=0.492\;C_{n}^{2}\;k^{7/6}\;L^{11/6}, \end{array}$$

Additionally, the normalized variance in the irradiance, commonly known as the scintillation index ($\sigma_{N}^{2}$), can be expressed in relation to $\sigma_{x}^{2}$ and the eddies involved in the scattering process (α and β), respectively, as [6,91,94,98]

(26a) $$\begin{array}{cc} \sigma_{N}^{2}≜ & \frac{\langle h_{a}^{2}\rangle -{\langle h_{a}\rangle }^{2}}{{\langle h_{a}\rangle }^{2}} \end{array}$$

(26b) $$\begin{array}{cc} = & \frac{\langle h_{a}^{2}\rangle }{{\langle h_{a}\rangle }^{2}}-1 \end{array}$$

(26c) $$\begin{array}{cc} = & exp\left(4\sigma_{x}^{2}\right)-1 \end{array}$$

(26d) $$\begin{array}{cc} = & 1/\alpha +1/\beta +1/(\alpha \beta ). \end{array}$$

Málaga (M)-Distribution: The M-distribution serves as a versatile model suitable for characterizing the entire range of turbulent regimes. Table 2 outlines the methods for deriving current AT models from the M-distribution model [99]. The M-distributed fading model incorporates components like $U_{L}$, $U_{S}^{C}$, and $U_{S}^{G}$. These components denote the LoS contribution; the scattered component by eddies along the propagation axis coupled to the LoS; and the scattered component by off-axis eddies received by the Rx, respectively. The average power of the LoS component and the total scatter components are given by $\Omega =E[|U_{L}{|}^{2}]$ and $2b_{0}=E[|U_{S}^{C}{|}^{2}+|U_{S}^{G}{|}^{2}]$, respectively.

Furthermore, the average power of the scattering component coupled to the LoS and the scattering component received from off-axis eddies are $E[|U_{S}^{C}{|}^{2}]=2\rho b_{0}$ and $E[|U_{S}^{G}{|}^{2}]=\left(1-\rho \right)2b_{0}$, respectively. The parameter 0≤ρ≤1 denotes the proportion of scattering power coupled to the LoS component. The PDF of the optical channel gain $h_{a}$ for the M-distribution is expressed as [99,100]

(27) $$f_{h_{a}}\left(h_{a}\right)=A\sum_{k=1}^{\beta }a_{k}h_{a}^{\frac{\alpha +k}{2}-1}K_{\alpha -k}\left(2\sqrt{\frac{\alpha \beta h_{a}}{\mu \beta +\Omega^{\prime }}}\right)$$

where $\mu =E[|U_{S}^{C}{\left|\right]}^{2}=\left(1-\rho \right)2b_{0}$, $\Omega^{\prime }=\Omega +2\rho b_{0}+2\sqrt{2\rho b_{0}\Omega }\cos\left(\varphi_{A}-\varphi_{B}\right)$, and $\varphi_{A}$ and $\varphi_{B}$ denote the deterministic phases of the LoS component and the coupled-to-LoS component, respectively. The parameter α is a positive value dependent on the effective number of large-scale cells in the scattering process, while β is a natural number representing the fading parameter. The terms *A* and $a_{k}$ can be defined respectively as [99,100]

(28a) $$\begin{array}{c} A≜\frac{2\alpha^{\frac{\alpha }{2}}}{\mu^{1+\frac{\alpha }{2}}\Gamma \left(\alpha \right)}{\left(\frac{\mu \beta }{\mu \beta +\Omega^{\prime }}\right)}^{\beta +\frac{\alpha }{2}} \end{array}$$

(28b) $$\begin{array}{c} a_{k}≜\left(\begin{array}{cc} \beta & -1\\ k & -1 \end{array}\right)\frac{{\left(\mu \beta +\Omega^{\prime }\right)}^{1-\frac{k}{2}}}{(k-1)!}{\left(\frac{\Omega^{\prime }}{\mu }\right)}^{k-1}{\left(\frac{\alpha }{\beta }\right)}^{\frac{k}{2}} \end{array}$$

Aggregate Attenuation Data: The PDF of $h=h_{\ell }h_{a}h_{p}$, representing the characteristics of the propagation channel, can be represented as [89,101]

(29) $$f_{h}\left(h;w_{z}\right)=\int f_{h|h_{a}}\left(h|h_{a}\right)f_{h_{a}}\left(h_{a}\right)dh_{a}$$

where $f_{h|h_{a}}\left(h|h_{a}\right)$ represents the conditional probability given a turbulence state $h_{a}$, with its distribution expressed as [89,101]

(30) $$f_{h|h_{a}}\left(h|h_{a}\right)=\;\frac{1}{h_{a}h_{\ell }}f_{h_{p}}\left(\frac{h}{h_{a}h_{\ell }}\right)=\;\frac{\gamma_{p}^{2}}{A_{0}^{\gamma_{p}^{2}}h_{a}h_{\ell }}\;{\left(\frac{h}{h_{a}h_{\ell }}\;\right)}^{\gamma_{p}^{2}-1},\;0\leq \;h\leq \;A_{0}h_{a}h_{\ell }.$$

Hence, $f_{h}\left(h;w_{z}\right)$ can be expressed as [89]

(31) $$f_{h}\left(h;w_{z}\right)=\frac{\gamma_{p}^{2}}{{\left(A_{0}h_{\ell }\right)}^{\gamma_{p}^{2}}}h^{\gamma_{p}^{2}-1}\int_{h/A_{0}h_{\ell }}^{\infty }h_{a}^{-\gamma_{p}^{2}}f_{h_{a}}\left(h_{a}\right)dh_{a}.$$

#### 3.2.3. FSO System Model Considering Jammer Interference

The FSO system model described in Equation (1) can be expanded to include a jamming source. In this scenario, a jammer, which is uncorrelated and synchronized with the user’s transmission time interval, emits random pulses with a certain level of jamming. Both the jamming and user signals travel through the FSO channel and reach the receiving photodetector. At the Rx, after signal conversion, the relationship between the input and output can be mathematically expressed as [46,86]

(32) $$\begin{array}{cc} r= & \eta_{e}hx+\sqrt{\Upsilon_{j}}h_{j}\Phi \end{array}$$

where $h_{j}$ represents the irradiance of the jamming source at the destination, $\Upsilon_{j}$ denotes the electrical power of the jammer, and Φ indicates the jamming state defined as

(33) $$\begin{array}{c} \Phi =\left\{\begin{array}{cc} 1 & \;\mathrm{In}\;\mathrm{the}\;\mathrm{presence}\;\mathrm{of}\;a\;\mathrm{jammer}\\ 0 & \;\mathrm{otherwise}. \end{array}\right. \end{array}$$

## 4. Enabling Technologies for FSO Communication

**Figure 8:**
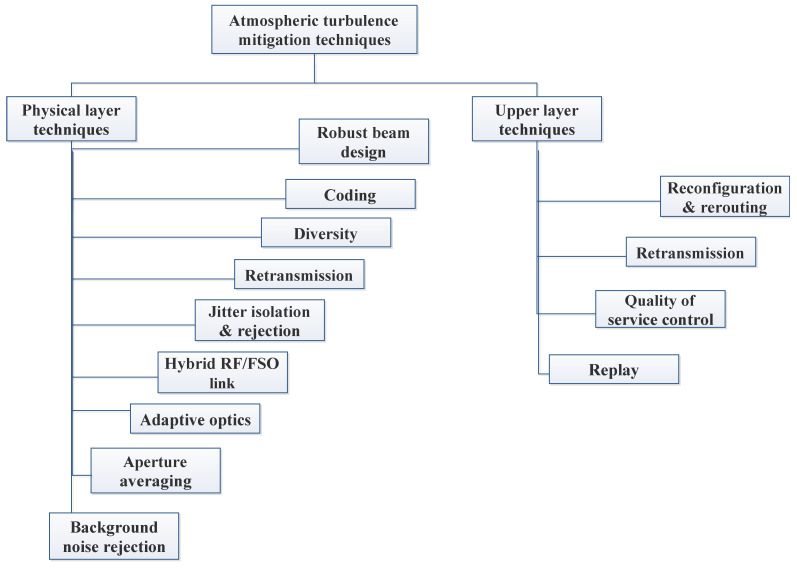
Techniques for mitigating atmospheric turbulence.

In the rapidly evolving field of FSO communication, various enabling technologies are pivotal in enhancing system performance and reliability. This section delves into key advancement such as AO, which corrects wavefront distortions; advanced modulation schemes that improve data throughput; and aperture averaging to reduce signal fading. Additionally, spatial diversity and cooperative relaying techniques are explored for their roles in mitigating adverse atmospheric effects. Adaptive transmission methods and robust ECC are also discussed, highlighting their contributions to maintaining signal integrity and optimizing communication efficiency in FSO systems. Methods for mitigating atmospheric turbulence are shown in Figure 8.

### 4.1. Robust Beam and Multi-Modulation Compatible System Design

The robust beam and multi-modulation compatible laser design focuses on enhancing optical communication systems by integrating a laser capable of supporting multiple modulation formats. This design improves signal integrity and system flexibility while reducing the complexity and size of the communication hardware. By enabling seamless switching between various modulation techniques, it optimizes performance for diverse communication scenarios, ensuring robust and efficient data transmission.

#### 4.1.1. Robust Beam Design

The propagation of laser beams through the atmosphere has been extensively studied both theoretically and experimentally. Recently, significant attention has been focused on laser beams with phase singularities moving through ideal atmospheric turbulence and non-Kolmogorov turbulence. Research indicates that partially coherent vortex beams are less impacted by atmospheric turbulence compared to partially coherent non-vortex beams, and that the topological charge of these beams can serve as an information carrier in optical communications [102].

To mitigate the disturbances caused by turbulence, extensive research has focused on the effects of non-diffracting and pseudo-non-diffracting vortex beams in resisting turbulent disturbances. The findings indicate that these beams’ favorable characteristics can significantly reduce the impact of turbulence on the transmission of OAM modes [103,104,105,106]. Specifically, experimentalists are seeking laser beams that are either non-diffracting or possess a definite value of photon OAM along the propagation direction. These specialized laser beams have proven useful in various applications, including optical tweezers, optical trapping, laser radar, metrology, remote sensing, microlithography, image processing, astronomical imaging, medical imaging and surgery, as well as wireless, optical, and FSO communications [107].

In OAM-based underwater optical wireless communication (OWC), the OAM mode is influenced by the wavefront spatial structure of the carrier. As a result, absorption, ocean turbulence, and scattering inevitably distort the OAM mode and cause crosstalk between its energy states. To mitigate the perturbations caused by seawater turbulence, employing various beam structures has become a new trend in this field. These structures include the Gaussian Schell-model (GSM) vortex beam [102], random frozen photons (RFP) beam [108], Hermite–Gauss (HG) vortex beam [109], perfect Laguerre–Gauss (PLG) beam [110], and several quasi-non-diffractive beams such as the Bessel–Gauss (BG) vortex beam [111,112], Lommel–Gauss (LMG) beam [113], Airy circle (AiC) vortex beams, Airy ellipse (AiE) vortex beams [114,115,116], and Hypergeometric-Gaussian (HyGG) vortex beam [103].

Meanwhile, most previous studies on OAM-based systems have concentrated on Laguerre–Gaussian (LG) beams in turbulent atmospheres. In contrast, non-diffraction BG beams exhibit clear advantages over traditional LG beams in reducing turbulence effects and enhancing the performance of OAM-based FSO communication links in such conditions. These benefits arise from two notable characteristics of BG beams: their ability to propagate without altering their intensity profile (non-diffraction nature), and their exceptional capacity to reconstruct after encountering an obstacle (self-healing mechanism) [111,117]. The BG beam is essentially a Bessel beam (BB) truncated by a Gaussian profile, exhibiting nearly diffraction-free properties up to a specific distance known as the non-diffractive range (NDR). The primary advantage of BG beams over BBs is that the radial modulation tapers the aperture fields, thereby reducing the on-axis ripples caused by edge diffraction [118]. These characteristics are crucial for optical communications based on LoS operations. Consequently, for OAM-based FSO communication systems, the non-diffraction and self-recovery properties of BG beams make them highly valuable. The transmission characteristics of BG beams in free-space have been explored, and their non-obstruction properties in FSO communication systems are examined in [117,119].

Moreover, structured light beams, particularly pin beams (PBs), exhibit reduced scintillations in free space compared to Gaussian beams. A novel class of optical beams has been introduced, characterized by a main lobe with a Bessel-like transverse profile and a width that gradually increases during propagation. These are termed inverted pin beams (IPBs). Numerical simulations demonstrate that IPBs have a significantly lower scintillation index than other beam classes such as PBs, BBs, collimated Gaussian beams (CGBs), and focused Gaussian beams (FGBs) in atmospheric turbulence regimes [120,121]. Also, IPBs carrying OAM can further mitigate intensity scintillations under moderate to strong irradiance fluctuation conditions [122].

As a member of the pseudo-non-diffracting OAM-carrying beam family, HyGG vortex beams exhibit self-reconstruction and abrupt autofocusing properties. Notably, HyGG vortex beams can be converted into Gaussian mode, modified BG (MBG) mode, modified exponential Gauss (MEG) mode, and modified LG (MLG) mode by setting specific parameters of the beam source. Due to these characteristics, HyGG beams hold significant potential for use in OWC [103,104,105,106,123].

#### 4.1.2. Multi-Modulation Compatible Miniaturization System

Free-space optical (FSO) communication has been extensively used to establish satellite-to-earth, inter-satellite, and deep-space links for purposes such as environmental monitoring, information transmission, and celestial exploration [124]. The electro-optical conversion in FSO links typically relies on various modulation techniques, including intensity modulation (IM) and phase modulation (PM). IM is widely utilized due to its simple architecture and ease of operation [125,126]. In contrast, PM is considered a more suitable solution for long-haul links, offering higher data rates and improved receiving sensitivity [127,128]. Therefore, achieving compatibility between IM and PM within the same communication system is of practical significance.

Traditional IM and PM both rely on the Mach–Zehnder modulator (MZM) and its associated supporting devices [129,130]. As a result, an IM/PM-compatible system requires two independent sets of modulation modules along with numerous auxiliary devices, significantly increasing system complexity. However, the limited resources on satellites impose strict conditions on the size, weight, and power (SWaP) of laser communication terminals in practical applications. Therefore, solutions that enhance compatibility and allow for seamless IM/PM switching while maintaining optimal SWaP are highly desirable.

Multi-modulation-format compatible laser communication refers to a laser communication system designed to support and efficiently manage multiple modulation formats. This capability allows for seamless communication between different devices. Such a system can dynamically switch between or simultaneously support various modulation formats to optimize performance based on changing conditions, including channel conditions, data rate requirements, power efficiency, and compatibility with various systems and standards. By being adaptable to multiple modulation formats, a laser communication system enhances its flexibility, efficiency, and reliability across diverse applications and environments.

Since the intensity or phase change of an optical signal can be controlled by the input electrical level of a chirp-managed laser (CML) [131,132], the CML has emerged as a convenient solution for achieving IM/PM compatibility. This CML-based modulation technique eliminates the need for traditional Mach–Zehnder modulators (MZMs) and their supporting devices, thus reducing the size, weight, and power (SWaP) requirements and aiding in the miniaturization of optical communication terminals. In [133], a CML-based IM/PM compatible transmitter is designed, demonstrating the ability to switch seamlessly between On–Off Keying (OOK) and return-to-zero differential phase-shift keying (RZ-DPSK) using a single set of devices.

### 4.2. Mode Diversity Reception

Using lasers as information carriers, FSO communication with fiber-based optical receivers presents an excellent solution for achieving high data rates, ultra-wide bandwidth, and low power consumption [134]. However, atmospheric turbulence during free-space propagation induces refractive index disturbances. This results in laser signals experiencing beam spread, beam drift, intensity scintillation, and angle-of-arrival fluctuations. These effects reduce the coupling efficiency of the spatial optical beam to the fiber, leading to significant power fading and scintillation, which consequently degrades the performance of practical laser transmission systems [135,136].

Several methods have been investigated to mitigate the effects of atmospheric turbulence and enhance the performance of high-speed FSO communication systems. One of the most commonly used and effective technologies is the AO system, which improves the coupling efficiency of free-space light into a single-mode fiber (SMF) by correcting wavefront aberrations within the aperture [137,138]. However, AO FSO systems still face challenges in terms of reliability under strong turbulence. Due to the limited spatial resolution and response time of the optics, AO does not achieve perfect wavefront correction in these conditions [139]. Additionally, high-speed FSO communication with bandwidths on the order of GHz demands higher accuracy and faster response times from AO systems, which can result in slight deficiencies in communication system reliability under strong turbulence [140].

Modes diversity offers a method to counteract wavefront distortion within the aperture, thereby enhancing the performance of FSO communication systems under atmospheric turbulence [139]. To this end, mode diversity reception (MDR) has been proposed and examined as an effective strategy for improving the reception performance of high-speed FSO communication systems. This approach involves receiving optical signals using few-mode fibers (FMFs) or multimode fibers (MMFs) that support multiple orthogonal modes [140,141,142]. Furthermore, the distorted wavefront caused by atmospheric turbulence can be considered a combination of a fundamental mode and multiple high-order modes. MDR captures as many high-order mode optical signals as possible. Using mode demultiplexers, the turbulent light is effectively split into an array of SMFs. Different methods are then used to combine the outputs from the single-mode ends of the demultiplexers to enhance signal quality. The probability of all copies, which carry the same information in the SMF array, simultaneous experiencing deep fading is low. Thus, mode diversity technology effectively improves the quality of the received signal and can be applied to satellite-to-ground optical communication systems [143].

The atmospheric turbulence compensation scheme using MDR involves two key technologies: mode reception and signal combination. In fiber-based MDR systems, mode demultiplexers are essential for decomposing spatial light affected by turbulence into SMFs. The two common types of mode demultiplexers used in these systems are multi-plane light converters (MPLCs) and photonic lanterns (PLs). Additionally, there are three primary methods for combining multichannel signals: equal gain combining (EGC), selection combining (SC), and maximum ratio combining (MRC) [140,141,142,144]. For different FSO communication scenarios, numerous studies have explored MDR using various mode demultiplexers and combining technologies to enhance system performance. For instance, ref. [145] proposes and verifies two simplified MDM schemes for FSO communication systems under moderate-to-strong turbulence conditions. These MDM schemes are designed and implemented for coherent detection in FSO communication systems using FMF coupling. The first scheme replaces the coherent receiver with a KK receiver (field reconstruction), significantly reducing the number of photodetectors and high-speed analog-to-digital converters by 75%. The second scheme achieves few-mode KK detection by employing a few-mode local oscillator and a single PD, thereby simplifying the MDM system to an extreme level. Experimental results indicate that an FSO communication system utilizing six-mode fiber demonstrates an average received power gain of approximately 6 dB over a system based on SMF under moderate-to-strong turbulence conditions. The two proposed MDRs exhibit similar performance, with an improvement of 5 dB to 6 dB compared to SMF-based heterodyne detection in the same turbulence conditions. Moreover, the simplified receiver schemes reduce both the volume and cost of the optical receiver, offering practical benefits for FSO communication systems.

### 4.3. Modulation Schemes

Modulation schemes are fundamental to the robustness and efficiency of FSO communication systems. The choice of modulation technique directly impacts the data rate, power efficiency, and resilience of the communication link, particularly under varying atmospheric conditions. For instance, conventional modulation techniques such as OOK and Pulse-Position Modulation (PPM) are commonly used, but they often suffer from performance degradation under adverse atmospheric conditions. Also, advanced modulation schemes like QAM and Orthogonal Frequency-Division Multiplexing (OFDM) can be employed to improve data rates and resilience in FSO systems. In addition, the emerging optical spatial modulation (OSM) technique can address the previously mentioned problems associated with conventional modulation schemes and Multiple-Input Multiple-Output (MIMO) setups. This section delves into both conventional and advanced modulation schemes, examining their characteristics, advantages, and limitations in the context of FSO communication. A summary of the literature on various modulation formats and their associated metrics for FSO is presented in Table 3.

**Table 3:** Literature summary on modulation formats and associated metrics for FSO.

Modulation Scheme	Metrics	Comments	Ref.
Numerical simulation			
PPM-MSK-SIM	BER	The BER performance of PPM-MSK-SIM surpasses that of both PPM and BPSK-SIM.	[146]
DOSM-PAM	ASER, APEP	A DOSM scheme that eliminates the need for CSIR results in a slight performance degradation compared to the conventional scheme with perfect CSIR.	[147]
*Q*-ary PPM	BER	The suggested *Q*-ary PPM FEC-based approach offers outstanding coding gain.	[148]
OOK, DPSK, DQPSK	BER	DPSK offers modulation gains of 3.2 dB over the OOK format in strong turbulence and 4.5 dB in weak turbulence. In moderate and strong turbulence, DPSK and DQPSK formats exhibit nearly identical BER performance.	[149]
MPAM	BER	The adaptive LDPC-coded modulation scheme can tolerate deep fades of 30 dB and higher in strong turbulence conditions.	[150]
MPPM	BER	Simulative evidence indicates that RL-MLC MPPM, when used with standard LDPC codes, can surpass the performance of any PPM scheme under identical transmission constraints.	[151]
OSM	ABEP	OSM can provide performance on par with conventional coherent FSO systems using spatial diversity, while also surpassing them in spectral efficiency and hardware simplicity.	[152]
SIM/SM	BER	The SIM/SM scheme can surpass the performance of conventional SIM in both AWGN and outdoor channels across different spectral efficiencies.	[153]
SCPPM/SCMPPM	BER	In comparison to the SCPPM, the SCMPPM offers improved BER performance under a peak power constraint and is capable of achieving higher data rates.	[154]
CPolSK	BER	The CPolSK system requires ≈ 3 dB less SNR compared to the OOK system.	[155]
OOK, M-QAM-OFDM	BER	System utilizing hybrid WDM-PON-FSO technology has the potential to achieve high data rates.	[156]
OSSK	BER, EC	AT and PE have an insignificant impact on the performance of the OSSK-based system.	[157]
COIQSM	BER	COIQSM provides an efficient way to improve the reliability and resilience of FSO systems against atmospheric turbulence.	[158]
Experimental demonstration			
CAP	BER	The proposed CAP and STBC scheme is minimally affected by air turbulence. It can be scaled up for higher speeds and extended for a greater interconnection range.	[159]
PAM-4-PolM-DD	BER	The PolM-DD system offers greater reliability compared to the conventional IM-DD FSO system.	[160]
OOK, BPSK, PPM	SER, *Q*-factor	PPM delivers superior performance in weak turbulence environments compared to OOK and BPSK.	[161]
SCOM, PM	BER	SCOM reduces RF power fading and interference. PM scheme with remotely injection-locked DFB LD has high resilience to noise and distortion.	[162]
M-QAM-OFDM, WDM	BE, BER	HH, PTS, and AC algorithms provide more than a 21% improvement in capacity. WDM can further enhance this capacity.	[163]
M-QAM-DMT	$Q^{2}$ factor, BER	In under weak turbulence conditions, the $Q^{2}$ factor of the M-QAM DMT signals with FEC encoding schemes maintains its maximum value and is improved compared to the regular scheme.	[164]

OOK: On–Off Keying, PM: Phase Modulation, PSK: Phase-Shift Keying, DPSK: Differential PSK, DQPSK: Differential Quadrature PSK, PAM: Pulse Amplitude Modulation, PolM: Polarization Modulation, PPM: Pulse-Position Modulation, MPPM: Multipulse PPM, SCPPM: Serially Concatenated PPM, SCMPPM: Serially Concatenated MPPM, CPolSK: Circle Polarization Shift-Keying, M-QAM: *M*-ary Quadrature Amplitude Modulation, OSSK: Optical Space Shift Keying, COIQSM: Coded-Optical Improved Quadrature Spatial Modulation, CAP: Carrierless-Amplitude-Phase Modulated, DMT: Discrete Multi-Tone, SCOM: Single-Carrier Optical Modulation, SIM: Subcarrier Intensity-Modulation, SM: Spatial Modulation, DD: Direct detection, IM-DD: Intensity Modulation and Direct Detection, OSM: Optical Spatial Modulation, DOSM: Differential OSM, FEC: Forward Error Correction, LDPC: Low-Density-Parity-Check, MLC: Multilevel Coding, RL-MLC: Reduced-Layer MLC, CSIR: Channel State Information at Receiver, BE: Bit Efficiency, BER: Bit-Error Rate, ASER: Average Symbol Error Rate, APEP: Average Pairwise Error Probability, ABEP: Average Bit Error Probability, EC: Ergodic Capacity, DFB LD: Distributed Feedback Laser Diode, AWGN: Additive White Gaussian Noise, AT: Atmospheric Turbulence PE: Pointing Errors, HH: Hughes-Hartogs, PTS: Partial Transmit Sequence, AC: Arithmetic Coding.

#### 4.3.1. Traditional Modulation Techniques

The performance impairments caused by scintillation can be alleviated through various methods, including enhanced error control coding [10,165], advanced modulation schemes [166], and diversity techniques [10,167,168]. In modulation schemes, the three main optical signal parameters that can be adjusted to achieve high data transmission rates are phase, polarization, and intensity [169]. Choosing the appropriate modulation scheme is crucial for mitigating scintillation in FSO communications. Various FSO modulation techniques, such as PSK, OOK, PAM, and Frequency Shift Keying (FSK), have been extensively studied and analyzed over the years [93,147].

Traditionally, OOK using IM/DD is the most commonly used modulation scheme due to its simplicity in design and implementation, as well as its cost effectiveness [170]. However, OOK-based FSO systems are suboptimal in AT channels and provide a lower SE compared to PAM [171]. The high SE of the PAM scheme comes with a trade-off of considerable performance degradation, as higher-order PAM is highly sensitive to Rx noise and AT [147]. Consequently, PPM has been extensively employed in FSO systems, despite its poor bandwidth efficiency and requirement for symbol synchronization between transceivers, adding significant complexity to the system [146]. Phase modulation schemes, including standard and differential PSK (DPSK), are prone to phase noise [172]. For instance, DPSK is frequently employed but experiences fluctuations in the phase of the encoded signal as a result of scintillation. Differential Quadrature PSK (DQPSK) offers double the SE of binary modulations like DPSK and OOK, as it transmits two bits per symbol with four potential optical phase fluctuations in successive symbol periods [169]. Further advancements have utilized coherent digital modulation schemes such as QAM. While coherent modulations have shown improved data rates, their power efficiency limitations and complexity become more significant at higher data rates [158].

#### 4.3.2. Advanced Modulation Techniques

Spectrally efficient QAM is widely used and preferred for various applications because it modulates both amplitude and phase. In fact, QAM encompasses a family of modulation techniques with different constellation types, each corresponding to distinct arrangements of the signal points [173]. So, for spectrally efficient high-data rate transmission, higher-order modulation techniques like the QAM family—which includes rectangular QAM (RQAM), squared QAM (SQAM), hexagonal QAM (HQAM), and cross QAM (XQAM)—are gaining increased attention in wireless communication systems due to their high power and bandwidth efficiency. In SQAM, constellation points are positioned at the vertices of a square lattice, suitable only for constellations with even powers of 2. To better adapt to varying channel conditions and improve spectral efficiency, incorporating constellations with odd powers of 2 offers greater flexibility [174,175,176]. RQAM is typically preferred for these odd-power constellations, as it is a versatile modulation scheme, encompassing orthogonal binary FSK (OBFSK), QPSK, multilevel Amplitude Shift Keying (ASK), and SQAM as special cases. However, RQAM is not ideal for odd-power constellations; instead, a modified XQAM constellation is favored for its reduced peak-to-average-power ratio (PAPR) and enhanced power efficiency. XQAM constellations are formed by removing the outer corner points of RQAM constellations and arranging them in a cross shape, minimizing the average power of the constellation [174,177].

Furthermore, the growing demand for high-data rates has led to the development of an optimal two-dimensional (2D) hexagonal-shaped constellation known as HQAM. HQAM features the densest 2D packing for a given optimal Euclidean distance between constellation points, resulting in improved power efficiency with lower peak and average powers as the constellation size (*M*) increases. Consequently, HQAM offers better BER/SER performance compared to other QAM schemes [174,175,177]. Depending on the trade-off between detection complexity and power efficiency, HQAM constellations are classified as regular and irregular HQAM structures. While regular HQAM has simpler detection, its power efficiency and BER performance can be further enhanced for larger *M* values. Irregular HQAM provides superior power efficiency and optimal performance, though it comes with increased detection complexity [174].

Additionally, carrier recovery and automatic gain control (AGC) are necessary when using SQAM with differential coding, but these are challenging to implement in practice. Carrier recovery faces significant issues, including false locks. To eliminate the need for AGC and address the false lock problem, the star QAM constellation is introduced. Star QAM, a special case of circular amplitude PSK (APSK), outperforms SQAM in peak power-limited systems. It features multiple concentric PSK circles with equal constellation points and identical phase angles. The amplitude and phase of the constellation points are mutually independent, allowing for successful differential detection rather than coherent detection, thus removing the need for precise phase tracking and channel estimation at the Rx. Due to these advantageous characteristics, star QAM has been adopted in various satellite communication standards.

Conversely, the state of polarization is significantly less affected by turbulence-induced fading compared to phase and amplitude. Consequently, POLSK presents a viable alternative to optical intensity and phase modulation methods, owing to its resistance to phase noise and efficiency for long-distance operation [172]. Based on this advantage, a non-coherent binary POLSK (BPOLSK) technique has been proposed, utilizing the state of polarization of a fully polarized beam with two orthogonal channels as the information-bearing parameter [167,178]. In this context, POLSK-based schemes have been demonstrated to offer 3 dB better sensitivity than OOK. However, the effectiveness of POLSK in standard fibers is limited due to fiber birefringence. The polarization state changes unpredictably, making it difficult to track at the Rx. Conversely, POLSK does not encounter these issues in FSO communication systems. Theoretically, AT causes minimal depolarization, and there is negligible crosstalk between orthogonal polarization states during transmission. The polarization state of an optical wave is well preserved over distances of several kilometers, allowing reliable detection at the Rx [179]. Therefore, since turbulence only causes intensity distortion, while the polarization state remains stable in FSO channels [160,179], the use of polarization modulation (PolM) is both feasible and attractive for enhancing system performance and reducing turbulence-induced fading in FSO systems [167]. Additionally, subtracting the two signals reduces common mode noise. PolM-DD FSO communication systems for OOK signals have been extensively studied and shown to achieve an approximate 3 dB sensitivity gain compared to IM [155,160,180].

Turbulence-induced scintillation can be significantly mitigated by utilizing redundancy offered by multiple Txs and/or Rxs. Consequently, POLSK is also employed in MIMO FSO systems, as demonstrated in [181,182,183]. These systems typically investigate coherent BPOLSK or coherent multilevel POLSK (MPOLSK). However, the non-coherent BPOLSK system described in [178] has not been taken into account in a MIMO architecture. To address this, an innovative non-coherent MIMO optical modulation technique, termed PPM-BPOLSK, has been introduced. This approach integrates the implementation simplicity and performance benefits of non-coherent BPOLSK with the power efficiency of PPM. It has been shown that the proposed method offers enhanced error performance in AT channels compared to systems that rely solely on BPOLSK or PPM, establishing it as an effective alternative to other IM/DD techniques regarding power efficiency [167]. Also, assuming bandwidth is virtually unlimited, the main design emphasis in FSO systems has been on power efficiency instead of SE [167,184]. Consequently, MIMO FSO systems are typically designed using Space–Time Coding (STC) or repetition coding (RC) on the transmission side (see Section 4.6), along with diversity combining or aperture averaging techniques on the reception side to address scintillation effects. Additionally, utilizing multiple Txs and Rxs helps prevent beam blockage and enables coverage over greater distances [184].

OFDM is an extensively utilized method for broadband wireless communications, renowned for its inherent advantages such as enhanced resilience to frequency-selective fading, low cost, efficient implementation, narrow-band interference, and high spectral and power efficiency. As a result, OFDM has been incorporated into various high-speed digital communication standards and is being regarded as a potential solution for future mobile wireless systems [98,185,186,187]. Despite its disadvantages, such as sensitivity to phase noise and a high PAPR, OFDM can be optimized to maximize its benefits and mitigate its drawbacks for FSO channels through careful design [186,188]. Coherent detection is another promising technique for FSO channels, significantly enhancing Rx sensitivity [189]. Therefore, combining OFDM with coherent detection presents considerable potential for future FSO communication systems [186,188].

OFDM is a type of multicarrier transmission where high data rate streams are divided into lower rate streams and transmitted simultaneously over multiple narrow-band subcarriers. These subcarriers are modulated using PSK or QAM and are carried on a high-frequency carrier [98,186,187]. For example, in OFDM-based coherent FSO systems using multilevel PSK (MPSK) or *M*-ary (multilevel) QAM (M-QAM), it is necessary to employ additional pilot tones or training sequences for channel estimation and equalization. This is to counteract the amplitude and phase variations resulting from turbulence in the channel and laser phase noise. Consequently, this increases system complexity and reduces SE. Conversely, multilevel DPSK (MDPSK) shows great potential for OFDM-based coherent FSO systems due to its strong resilience to amplitude fading and phase distortion. MDPSK can mitigate the disadvantages of OFDM’s sensitivity to phase noise and ensure good system performance without the need for equalization [188,190].

**Figure 9:**
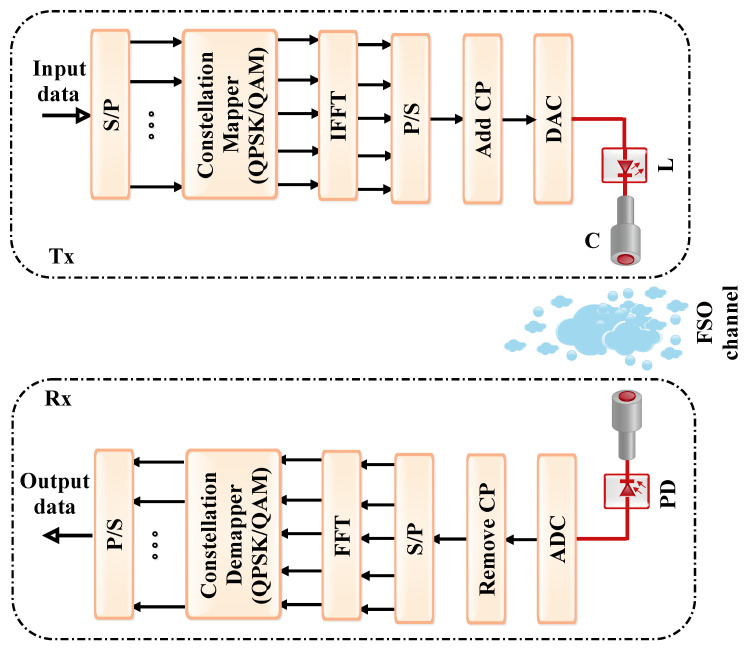
Basic setup for OFDM signal transmission. C: Collimator, PD: Photodetector, ADC: analog-to-digital converter, DAC: Digital-to-Analog Converter, FFT: Fast Fourier Transform, IFFT: Inverse Fast Fourier Transform, CP: Cyclic Prefix, S/P: Serial-to-Parallel, P/S: Parallel-to-Serial, L: Laser, Tx: Transmitter, Rx: Receiver.

In OFDM, the spacing between adjacent subcarriers is meticulously chosen so that each subcarrier aligns with the spectral nulls of all the others, allowing them to be packed as closely as possible. This careful arrangement ensures spectral orthogonality, meaning each subcarrier is orthogonal to the others. This orthogonality enables the subcarriers to be separated at the Rx using correlation techniques, thereby eliminating intersymbol interference between channels. The orthogonal carrier set is generated by applying the Inverse Fast Fourier Transform (IFFT) at the Tx and the Fast Fourier Transform (FFT) at the Rx, thus negating the need for equalization. Figure 9 illustrates the fundamental setup of OFDM signal transmission over an FSO link [98,186,187].

The transmission of OFDM through an optical link is a specialized form of multiple-subcarrier modulation (MSM). In this method, multiple independent bit streams are modulated onto subcarriers at different frequencies, combined in the RF domain, and transmitted via high-capacity optical links employing IM/DD. The MSM signal undergoes minimal distortion and does not require equalization at the Rx. Each subcarrier functions as an individual narrow-band signal with a reduced symbol rate [98].

The baseband OFDM signal is inherently complex and bipolar, but an IM/DD optical link requires a real and positive RF signal to drive the LD. To create a real OFDM signal, Hermitian symmetry is applied to the input vector of the Tx’s IFFT block. To convert the OFDM signal to unipolar, a DC bias is added (DC-OFDM), making the signal positive. This DC bias must be sufficiently large to avert clipping and distortion in the optical domain. However, the primary drawbacks of IM/DD MSM systems are the inefficiency in average optical power owing to the large DC bias and the distortions caused by the nonlinearities of the LD and optical channel. This issue holds particular significance for IM/DD DC-OFDM systems, where a significant number of subcarriers result in high PAPR [188]. Additionally, the LD’s nonlinearity induces interference between subcarriers and broadens the overall signal spectrum. These factors create a complex scenario and impose strict demands on the linearity of optical devices to minimize distortion and ensure satisfactory performance [98].

Furthermore, one way to enhance SE is by using amplitude modulation techniques, such as PAM and QAM, with higher-order constellations [167,184]. However, these multilevel modulation methods come with increased hardware costs, as higher modulation levels lead to greater PAPR and increased sensitivity to noise and device nonlinearities [83]. High PAPR values can push Txs beyond their linear operating range, making large PAM constellations less desirable [184].

Moreover, systems utilizing subcarrier intensity modulation (SIM) can enhance SE [83,167,184,191]. Consequently, the performance of subcarrier binary PSK (BPSK) IM in FSO systems has been thoroughly examined [92,191,192,193,194,195]. When compared to conventional SIM in FSO communication, BPSK-SIM demonstrates superior BER performance relative to other modulation schemes such as QPSK, MPSK, DPSK, and QAM [83,146,192,196]. To further improve the BER performance of FSO systems utilizing BPSK-SIM, a hybrid modulation scheme called PPM-MSK-SIM, which integrates PPM and MSK SIM, has been proposed [146]. However, the complexities of both the Tx and Rx in SIM systems are considerably higher than those found in pulse-based optical communications. Additionally, SIM systems face the challenge of requiring a time-varying DC bias to restrain the transmitted signal from going negative [83,167,184,191].

MIMO spatial multiplexing (SM) techniques are also taken into account when designing spectrally efficient FSO systems. However, these systems face several challenges, including inter-channel interference (ICI) from simultaneous transmissions, increased complexity in transceiver design to mitigate ICI, high computational complexity, the need for inter-antenna synchronization, and the need for numerous components to implement MIMO transceivers [152,167,184]. SM, initially developed for antenna-based systems, marked a shift towards free inter-antenna synchronization (IAS) [197,198]. In this approach, only one antenna is activated during each time slot, keeping the other transmission antennas inactive. This means SM requires just a single RF chain, making it a more cost-effective and less complex MIMO transmission structure that is resilient to IAS and ICI [158].

#### 4.3.3. Advanced Optical Spatial Modulation Techniques

In light-based communication systems, LEDs have a relatively low modulation bandwidth, which greatly restricts their transmission rates. Additionally, conventional SISO techniques do not provide spatial diversity gains. To tackle these challenges, MIMO technology, similar to its use in RF communication, has been integrated into various OWC systems. A MIMO setup using SM can also enhance SE. However, as previously mentioned, the development of multiple antenna transmission schemes encounters several challenges.

The challenges associated with conventional modulation schemes and MIMO setups can be addressed by a novel technique known as OSM, where only a single transmit aperture (LED/laser) is active at any moment [158,199,200]. In these configurations, light sources replace antennas, and optical chains substitute for RF chains. During transmission, information is transmitted through both the signal and antenna spaces simultaneously. Specifically, a portion of the incoming bits is utilized to select a LED/laser, while the remaining bits choose the symbols to be transmitted from the selected LED/laser. By incorporating the LED/laser index as part of the symbol, additional bits can be transmitted, enhancing the system’s SE without resorting to higher-order PAM or PPM. A particular case of OSM is optical space shift keying (OSSK), where transmit aperture indices alone are used to transfer information, simplifying the system compared to OSM. Since only one laser is active during each signaling period, issues related to ICI and antenna synchronization are completely avoided in OSM/OSSK, thereby reducing the design complexity of MIMO transceiver chains [152,167,184]. These inherent advantages make OSM/OSSK an ideal modulation technique for light-based communication systems such as underwater OWC (UOWC), visible-light communication (VLC), and FSO communication [201].

Furthermore, in OSM, the constellation size dictates the number of bits transmitted per symbol; larger constellation sizes can enhance SE but may also increase Rx complexity. Additionally, research indicates that the error performance of OSM surpasses that of alternative MIMO transmission techniques with equivalent SE, making it a viable option for FSO communication [152,167,184]. OSM can reduce the number of required constellation levels for a given SE without compromising power efficiency and while simplifying Rx complexity in comparison with MIMO SM techniques. This requirement can be minimized even further by incorporating the polarization dimension. Moreover, utilizing polarization alongside the Tx index and symbol dimensions could enable a corresponding MIMO system with half the number of Txs and Rxs, enhancing space efficiency [167]. Consequently, researchers have begun exploring FSO-OSM systems in combination with coherent detection, capitalizing on the seamless integration of their advantages. This exploration has led to significant improvements in both SE and transmission capacity [158]. An innovative coherent MIMO OSM architecture, which integrates the spatial modulation (SM) principle with MPOLSK, termed SM-MPOLSK, has been proposed. By allocating one bit to the additional polarization dimension, this new OSM approach necessitates smaller constellations or fewer transmit apertures than previous methods. Additionally, due to POLSK’s inherent resilience to channel scintillation, this OSM method, which employs the polarization dimension along with multiple transmit/receive apertures, demonstrates enhanced error performance in AT conditions [167].

Researchers have recently created new OSM structures to enhance SE. This innovation enables the use of multiple Txs, leading to increased data rates and a flexible optical chain. This category of OSM is known as an *advanced OSM* system, which employs multiple light sources simultaneously for data transmission, resulting in higher data rates and more effective utilization of available spectrum bandwidths [158]. Advanced techniques for OSM include Optically Enhanced SM (OESM), Optical Generalized SM (OGSM), and Optically Improved Quadrature SM (OIQSM) [202,203,204,205,206,207,208,209,210].

In OESM, the number of active light sources varies based on the signal constellation. If the size of the primary constellation is *m*, where $m=\log_{2}\left(M\right)$, then OGSM employs $N_{opt}$ optical chains, where $N_{opt}$ light sources are activated by $N_{L}$ light sources. The optimal $N_{opt}$ for achieving maximum SE should lie between $\left\lfloor \frac{N_{L}}{2}\right\rfloor$ and $N_{L}$. For enhanced SE, OIQSM separates two symbols from a constellation into their real and imaginary components, which are transmitted independently using two sets of light sources. Table 4 summarizes the SEs and optical chains required for these advanced SM techniques. Overall, OIQSM provides the highest SE with the smallest constellation size, requiring one optical chain for OSM and four for OIQSM.

Although, OSM system improves SE, it also leads to higher hardware costs and increased power consumption. These associated costs vary across different schemes. For instance, the power consumption, $P_{tot}^{\left(\cdot \right)}$, number of switches, $N_{sw}^{\left(\cdot \right)}$, and total hardware expenses, $C_{\left(\cdot \right)}$, for OSM, OESM, OGSM, and OIQSM are expressed as [199]

(34a) $$\begin{array}{cc} P_{tot}^{OSM}= & P_{oc}+\alpha_{tr}P_{t}+P_{sw}N_{sw}^{OSM}, \end{array}$$

(34b) $$\begin{array}{cc} P_{tot}^{OESM}= & 2P_{oc}+\alpha_{tr}P_{t}+P_{sw}N_{sw}^{OESM}, \end{array}$$

(34c) $$\begin{array}{cc} P_{tot}^{OGSM}= & P_{oc}N_{opt}+\alpha_{tr}P_{t}+P_{sw}N_{sw}^{OGSM}, \end{array}$$

(34d) $$\begin{array}{cc} P_{tot}^{OIQSM}= & 4P_{oc}+\alpha_{tr}P_{t}+P_{sw}N_{sw}^{OIQSM}. \end{array}$$

(35a) $$\begin{array}{cc} N_{sw}^{OSM}= & 2^{\left(\eta_{SE}-log_{2}\left(M\right)-1\right)}, \end{array}$$

(35b) $$\begin{array}{cc} N_{sw}^{OESM}= & 2^{(\eta_{SE}-log_{2}\left(M\right))/2-1}, \end{array}$$

(35c) $$\begin{array}{cc} N_{sw}^{OGSM}= & 1/4\left(1+\sqrt{1+4\times 2^{\left(\left(\eta_{SE}-2log_{2}\left(M\right)\right)+1\right)}}\right), \end{array}$$

(35d) $$\begin{array}{cc} N_{sw}^{OIQSM}= & 1/4\left(1\;+\;\sqrt{1\;+\;4\;\times \;2^{\left(\left(\eta_{SE}-2log_{2}\left(M\right)\right)/2+1\right)}}\right)\;. \end{array}$$

(36a) $$\begin{array}{cc} C_{OSM}= & C_{o}+C_{S/P}+C_{sw}N_{sw}^{OSM}, \end{array}$$

(36b) $$\begin{array}{cc} C_{OESM}= & 2C_{o}+C_{S/P}+C_{sw}N_{sw}^{OESM}, \end{array}$$

(36c) $$\begin{array}{cc} C_{OGSM}= & C_{o}N_{opt}+C_{S/P}+C_{sw}N_{sw}^{OGSM}, \end{array}$$

(36d) $$\begin{array}{cc} C_{OIQSM}= & 4C_{o}+C_{S/P}+C_{sw}N_{sw}^{OIQSM}. \end{array}$$

where $P_{t}$ represents the total transmitted optical power, $P_{oc}$ and $P_{sw}$ denote the power consumption of the optical chain and the optical switch, respectively. $\alpha_{tr}$ denotes the slope-dependent load factor, $\eta_{SE}$ represents the SE, and $C_{S/P}$, $C_{o}$, and $C_{sw}$ are the costs associated with the serial-to-parallel converter, the optical chain, and the optical switch, respectively.

**Table 4:** Spectral efficiency and optical chain for various modulation techniques.

Modulation	Spectral Efficiency (SE) [bpcu]	Optical Chain	References
SM	$\log_{2}M+\log_{2}N_{L}$	1	[158,199]
OESM	$\log_{2}M+2\log_{2}N_{L}$	2	[158,199,203,205,206]
QSM	$2\left\lfloor \log_{2}\left(\frac{N_{L}}{2}\right)\right\rfloor +2\log_{2}M$	4	[158,199,210]
OIQSM			
OGSM	log2NLNopt+Noptlog2M	$\left\lfloor \frac{N_{L}}{2}\right\rfloor \leq N_{\mathrm{opt}}\leq N_{L}$	[158,199,210]

bpcu: bits per channel use.

The quantity of switches needed for OGSM, OESM, and OIQSM is significantly lower than that for OSM at a given SE and M-ary modulation scheme. Consequently, the power consumption and switch costs are much lower for higher SEs in OESM, OGSM, and OIQSM. As SE increases, the number of switches in OSM grows exponentially, while in the other schemes, the growth is linear. This results in the total power consumption of OSM exceeding that of the advanced OSM schemes because of the high switch demand. It is important to note that the modest rise in power consumption due to multiple optical chains in advanced OSM schemes is balanced by the significantly higher power consumption of switches in OSM at extremely high SE levels. Additionally, the overall cost of OSM exceeds that of other advanced OSM schemes when achieving such high SE values. Therefore, as SE increases, advanced OSM schemes outperform OSM in both cost and power consumption [199].

In general, modulation schemes have a vital impact on the performance of FSO communication systems. However, the choice of modulation scheme in FSO communication systems involves trade-offs between simplicity, data rate, power efficiency, and resilience to atmospheric conditions. Conventional schemes such as PPM and OOK are easier to implement and more cost-effective but offer lower data rates and are more susceptible to adverse weather conditions. Advanced schemes like QAM and OFDM provide higher data rates and better resilience to AT but come with increased complexity and implementation costs. Also, the issues associated with conventional modulation schemes and MIMO setups can be addressed by OSM and its variants. Ongoing research and development in these advanced techniques are essential to overcoming the inherent challenges of FSO communication and unlocking its full potential in various applications.

### 4.4. Adaptive Transmission

This section presents adaptive transmission methods that dynamically adjust transmission parameters including, code rate, transmit power, and modulation size, to optimize communication performance. By adapting these parameters in real-time, these methods aim to maintain reliable and efficient data transmission even under varying channel conditions and interference levels. Also, by employing these adaptive transmission techniques, wireless communication systems can significantly enhance their performance, achieving a balance between reliability, efficiency, and speed. This dynamic adjustment capability is crucial for modern communication systems, including cellular networks, satellite communications, and emerging technologies like UAV-to-ground FSO systems, where varying conditions are the norm.

#### 4.4.1. Overview

Two important parameters that characterize turbulence-induced fading are $d_{0}$, the correlation length of intensity fluctuations, and $\tau_{0}$, the correlation time of intensity fluctuations. These parameters are defined respectively as [47,211,212].

(37a) $$\begin{array}{c} d_{0}\approx \sqrt{\lambda L}. \end{array}$$

(37b) $$\begin{array}{c} \tau_{0}=\frac{d_{0}}{u⊥}. \end{array}$$

where u⊥ denotes the component of the wind velocity vector perpendicular to the direction of propagation.

When the Rx aperture $D_{0}$ exceeds the correlation length $d_{0}$, turbulence-induced fading can be significantly mitigated through aperture averaging [211]. However, due to limitations on Rx size, it is not always feasible to ensure that the Rx aperture is much larger than the turbulent correlation length (i.e., $D_{0}>d_{0}$). In scenarios where $D_{0}<d_{0}$, aperture averaging loses its effectiveness, necessitating alternative methods to counteract intensity fluctuations [150,211,212]. These methods are categorized into two main types: spatial-domain techniques, which involve diversity detection using multiple Rxs, and time-domain techniques, which adaptively optimize the decision threshold based on the maximum likelihood criterion.

In temporal-domain techniques, a single Rx is employed. If the Rx knows the marginal fading distribution but lacks information about both the temporal fading correlation and the instantaneous fading state, a maximum-likelihood symbol-by-symbol detection technique can be utilized. Conversely, when the Rx is aware of the joint temporal fading distribution but not the instantaneous fading state, it can apply a maximum-likelihood sequence detection (MLSD) technique to counteract turbulence-induced fading.

Spatial-domain techniques necessitate at least two Rxs to capture signal light from various positions or angles. To optimize diversity reception gain, the Rxs should be positioned as far apart as possible to ensure that turbulence-induced fading remains uncorrelated between them. However, in practice, it may not always be feasible to place the Rxs at such a distance.

Although MLSD offers an optimal solution, it involves complex multidimensional integration and has a computational complexity that increases exponentially with the length of the transmitted bit sequence. To simplify MLSD, suboptimal implementations such as per-survivor processing (PSP) are often preferred in practice [186,212]. However, for BER below $10^{-6}$, both MLSD and PSP require an electrical SNR exceeding 20 dB, even in weak turbulence conditions. Such high signal powers are impractical for many applications, necessitating the development of novel modulation techniques for IM/DD FSO systems. In response, various coding approaches have been proposed, including coded OFDM [186], coded MIMO [213], and rateless coding [214,215].

The core idea behind the coded-OFDM method is to reduce the symbol rate through the use of OFDM. When combined with interleaving and low-density parity-check (LDPC) codes, this technique achieves high resilience to the deep fades characteristic of turbulent channels. However, OFDM is more susceptible to phase noise and has a comparatively high PAPR, necessitating meticulous design to maximize its benefits and mitigate its drawbacks. Given that OFDM in IM/DD systems may lack power efficiency, SSB clipped and unclipped OFDM schemes can be employed to enhance power efficiency [186].

In a coded-MIMO approach, multiple optical sources and detectors are utilized, based on either repetition MIMO concepts or Space–Time Coding, along with LDPC codes, to enhance resistance to AT [213]. Temporally correlated stochastic channels can benefit from rateless codes, which are ECC with adjustable rates to accommodate varying channel quality. Examples of such codes include punctured codes and fountain codes, with Raptor codes being a specific instance. Punctured codes vary the rate by dropping parity bits, thereby increasing the effective code rate. This method requires a base code with a minimum rate suitable for the worst-case channel condition. Puncturing schemes are typically defined and tested at each desired rate to ensure optimal performance. Conversely, Raptor codes adjust their rate by extending the codeword length, with the Tx generating coded bits continuously until the Rx decodes the transmitted word without error. Raptor codes have been previously proposed for the network layer of both wired and RF communication systems [215]. Demonstrations of Raptor and punctured codes in the PHY layer of an FSO link, under various turbulence conditions, have shown high tolerance to deep fades caused by AT [214]. However, the achievable information rates (lower bounds on channel capacity) indicate that these approaches are still several decibels below channel capacity, suggesting potential for further improvement [216].

#### 4.4.2. Adaptive Transmission Techniques

Adaptive modulation and coding (AMC), which is already utilized in wireless channels, facilitates reliable and spectrally efficient transmission over channels that vary over time [217]. The core concept involves estimating the channel conditions at the Rx and relaying this estimate back to the Tx via an RF feedback channel, allowing the Tx to adjust according to these conditions. Recently, adaptive transmission techniques have been integrated into FSO systems, showing promise as solutions to counteract AT and enhance FSO link performance [150,214].

A prevalent assumption in the existing literature is that open-loop implementations are used, where the Tx lacks knowledge of the channel conditions. Such open-loop designs are beneficial in time-varying channels, where obtaining feedback on channel estimates can be challenging. However, in the case of quasi-static channels, reliable feedback is attainable, and having available CSI at the Tx can enable the design of adaptive transmission schemes that significantly enhance performance. AT causes slow fading in FSO systems, with the coherence time of the optical channel ranging from 1 to 100 ms. This means that fading can remain constant over hundreds of thousands to millions of sequential bits at standard transmission rates, allowing the channel to be modeled as a slow fading process [20]. Consequently, adaptive transmission presents an attractive option for FSO systems.

**Figure 10:**
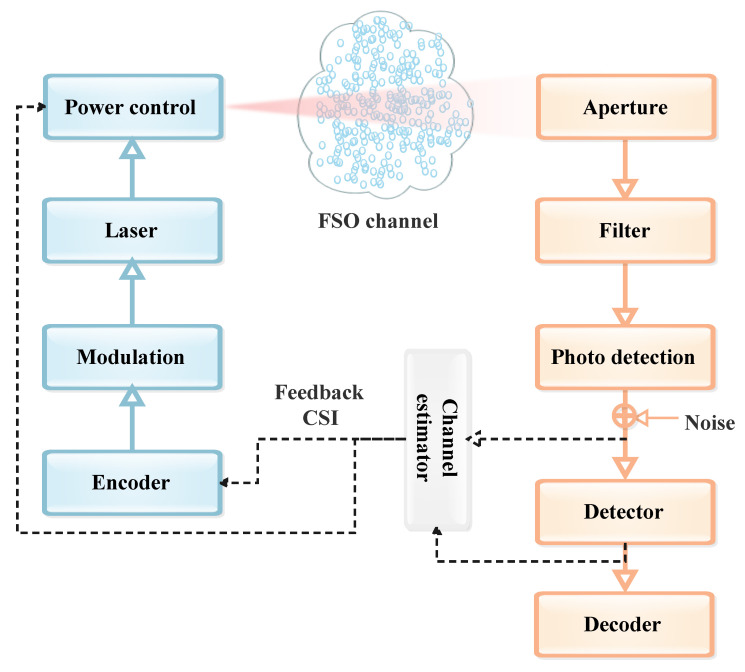
Block diagram of integrated power control and channel coding in an FSO communication system.

Figure 10 illustrates a typical block diagram of an integrated power control and channel coding system in FSO communication. The feedback information needed for adaptive transmission can be relatively easily implemented in FSO systems, as commercially available FSO units typically support bidirectional (full-duplex) communication. A small fraction of the extensive available bandwidth can be assigned for feedback without significantly impacting data rates. For instance, in some hybrid RF/FSO systems, the RF channel can be utilized for CSI feedback. This makes reliable CSI accessible in FSO systems, which is crucial for developing adaptive transmission schemes. As a result, to improve communication reliability and throughput, the Tx can adjust its transmission parameters—such as code rate, transmit power, and modulation size—based on the estimated current CSI [218,219,220,221,222]. Thus, various varieties of adaptive transmission systems have been suggested in the existing research on FSO communication, demonstrating their feasibility and advantages. For example, in [223], a straightforward rate-adaptive transmission technique is introduced that utilizes variable silence intervals and OOK formats with memory.

The study in [224] presents a statistical-delay quality of service (QoS)-aware adaptive modulation and power allocation method for a dual-channel coherent OWC system facing AT fading. The findings indicate that this adaptive scheme significantly outperforms conventional adaptive transmission methods under strict statistical-delay constraints and highlights the benefits of joint channel optimization in conditions of strong turbulence fading and tight statistical-delay requirements. Additionally, in [225], a joint relaying link selection and power allocation strategy that is statistical delay QoS-aware is proposed for the uplink of a multichannel coherent FSO communication-based fronthaul network. The analysis shows that to maximize the overall end-to-end (e2e) effective capacity, it is essential to account for both the statistical delay QoS needs of the transmitted traffic and the channel gains of the transmission links. The results further demonstrate that this approach enhances the statistical delay QoS-aware throughput despite the challenges posed by AT fading and PE.

An adaptive symbol rate, where the symbol duration adjusts based on channel conditions, along with the simultaneous adaptation of both the symbol rate and power, has been proposed to mitigate the effects of scintillation in FSO links [20]. In [14], the challenges of adaptive FSO transmission over AT channels are revisited, considering practical power constraints and modulation sizes. Two scenarios, (1) power adaptation with a fixed modulation size and (2) joint adaptation of power and modulation size, are explored. Also, an adaptive uncoded transmission algorithm that adjusts both transmit power and modulation size based on channel conditions is introduced. The optimization focuses on these adaptive parameters while adhering to peak power constraints. Simulation results indicate that these adaptive algorithms significantly enhance performance, particularly in conditions of intense turbulence.

In [222], a coded FSO system utilizing a fixed code rate and M-ary PPM under an average power constraint is investigated. The simulation results show that the proposed adaptive scheme offers considerable performance enhancements in relation to non-adaptive alternatives. In [226], the study examines rate-adaptive FSO communication using experimental data collected from a 1.87 km terrestrial FSO link under various weather conditions. To address the fluctuations in channel gain caused by AT and weather changes, a rate-adaptive communication system is implemented that punctures LDPC codes in a rate-compatible manner. In addition to the standard random puncturing method, an optimized intentional puncturing technique is also utilized. The evaluation results indicate that, unlike uncoded OOK, the rate-adaptive method ensures reliable and efficient FSO communication across different weather conditions and scintillation indices. Furthermore, applying intentional puncturing enhances the system’s efficiency in terms of throughput.

Moreover, a novel adaptive transmission technique for FSO systems, designed to operate under AT and utilizing subcarrier PSK (S-PSK) intensity modulation, is introduced in [227]. By leveraging the fixed envelope properties of S-PSK, this approach enhances the effective use of the FSO channel capacity by adjusting the modulation order of S-PSK in response to real-time turbulence-induced fading and a specified BER target. Numerical results demonstrate considerable gains in SE without increasing the average transmitted optical power or compromising BER, particularly in conditions of moderate to strong turbulence. Additionally, this variable-rate transmission technique is utilized in MIMO FSO systems, leading to further improvements in SE as the number of transmit and/or receive apertures increases. The performance of an FSO system for ground-to-satellite communication is examined in [228], focusing on the combined impacts of AT and beam wandering using MPSK. To enhance SE, an adaptive transmission strategy is implemented, optimizing channel capacity by adjusting the modulation order of the MPSK scheme based on the current channel conditions and the target BER. Additionally, a single-input multiple-output (SIMO) system employing MRC diversity is utilized to alleviate the impacts of AT and beam wandering.

Optical power allocation strategies for an adaptive Wavelength Division Multiplexing (WDM) radio over FSO (RoFSO) systems, utilizing variable wavelengths to reduce the impact of weather turbulence, are introduced in [220,229]. These strategies optimally distribute transmit power to maximize capacity and enhance the overall effectiveness of the WDM RoFSO system. Thus, within a total power constraint, varying optical powers can be assigned to selected wavelengths, leading to further enhancements in system performance [16]. A practical FSO system featuring an adaptive channel coding and power control scheme is examined in [221]. This approach allows for the simultaneous adjustment of both the coding rate and transmission power to minimize power consumption while adhering to practical constraints, such as the target OP, target BER, and transmit power limits imposed by eye-safety standards.

In [150], an AMC scheme is introduced as an effective method for addressing severe AT. The study explores three distinct adaptive modulation scenarios: (i) channel inversion with a fixed rate, (ii) variable-rate variable-power adaptation, and (iii) truncated channel inversion with a fixed rate. Additionally, the potential enhancements from using adaptive LDPC coded modulation (CM) are examined. The findings indicate that, under strong turbulence conditions, it is possible to withstand significant fades of 30 dB and higher [150].

Additionally, the FSO channel can exhibit a significant level of reciprocity in axially aligned bidirectional systems, meaning that the fading experienced at each end can be closely correlated [230,231]. This characteristic allows for immediate access to CSI at the Tx, eliminating the need for a separate feedback channel. Consequently, it is justifiable to assume the availability of CSI at both the Tx and Rx, which is essential for adaptive transmission [20].

Many proposed adaptive transmission methods based on CSI in FSO communication aim to enhance the reliability and throughput of these systems. However, these methods often assume that turbulence conditions remain constant. In reality, varying turbulence intensities at the same SNR can significantly impact communication quality. This suggests that while adaptive schemes may perform well under stable turbulence, their effectiveness can diminish or even degrade when faced with changing turbulence conditions. For example, an AMC scheme designed for weak turbulence may struggle to maintain the required BER in moderate or strong turbulence, leading to reduced communication quality or interruptions. Conversely, applying an AMC scheme intended for strong turbulence in milder conditions might result in a less efficient coding and modulation choice, ultimately lowering channel utilization and bit rate. To address these challenges, it is essential to develop an enhanced AMC scheme capable of adapting to FSO channels with varying turbulence intensity. Accordingly, an enhanced AMC scheme is proposed, incorporating a hybrid switching standard for UAV-to-ground FSO communication to mitigate the adverse effects of turbulence variation. This approach incorporates a channel estimator based on Gated Recurrent Units (GRU) and utilizes a hybrid switching standard linked to the frame error rate (FER) of LDPC decoding, aiming to optimize bit efficiency across different turbulence conditions.

### 4.5. Aperture Averaging

Aperture averaging is a crucial technique in FSO communication systems, employed to mitigate the adverse effects of AT. By averaging the optical signal over a larger aperture, this method effectively reduces signal fluctuations and enhances communication reliability. This section will explore the principles of aperture averaging, its advantages in mitigating the impacts of turbulence, and its implications for the future of FSO communication systems.

#### 4.5.1. Overview

A straightforward method to mitigate the fading effect is to employ a relatively large lens at the Rx to smooth out intensity fluctuations. This technique, commonly referred to as *aperture averaging*, serves as an inherent form of receive diversity. It involves adjusting the size of the detector or receive aperture to capture as much of the traversing optical field as possible. In FSO systems operating under strong and saturated conditions, aperture averaging is essential to address (1) signal scintillation or irradiance flux variations [232,233]; (2) address AoA fluctuations and boresight angle effects; and (3) to optimize the average signal power at the detector plane. Additionally, aperture averaging is a fundamental scheme that ensures spatial diversity [46]. In this context, aperture averaging can be considered a basic form of spatial diversity when the Rx lens aperture exceeds the fading correlation length [47,233,234].

Aperture averaging calculations are crucial in Rx design. The Rx needs to be large enough to gather adequate power and minimize scintillation effects at a specific distance, yet small enough to remain practical. Typically, the Rx does not require a significantly large diameter due to the nonlinear decrease in scintillation with increasing diameter. It is important to note that while aperture averaging becomes increasingly effective at reducing intensity variance as turbulence intensifies, this improvement is limited to a certain extent [233,235].

#### 4.5.2. Advantages of Aperture Averaging

To achieve effective FSO communication through continuous transmission link establishment, it is crucial to maintain a larger aperture area, resulting in a wider FoV. A narrow FoV limits the coverage area, which is not ideal for FSO communication systems. Although a larger collection aperture increases ambient light background noise, this can be mitigated by various methods that allow the FSO Rx to capture a larger portion of the incoming optical beam. Conversely, a higher FoV provides a larger coverage area and eliminates the need for additional circuitry for pointing and tracking mechanisms, ensuring reliable wireless connectivity between transmission and reception apertures. Moreover, FSO systems with a larger FoV encounter reduced misalignment errors, reducing the impact of obstacles between the source and destination points and easing the alignment control mechanism [46].

#### 4.5.3. Impact of Aperture Averaging

In FSO systems, the impact of aperture averaging is examined by taking into account various facets of these systems. For aperture-averaged FSO Rxs, it is widely recognized that the ΓΓ distribution is valid for a point Rx under a bounded Gaussian wave model; however, it does not support aperture averaging phenomena [236,237]. Also, experimental studies indicate that the LN model is valid for a point Rx in the weak turbulence regime and performs well in all turbulence regimes for aperture-averaged data [234,238]. Conversely, while the ΓΓ model is considered valid for a point Rx across all turbulence regimes, this validity does not extend to scenarios involving aperture averaging [234,238,239]. Additionally, it has been demonstrated that the LN and ΓΓ models become virtually identical when $D>6\rho_{0}$, where $\rho_{0}$ is the spatial coherence radius [240]. To analyze the impact, consider the PDF of the irradiance, $h_{a}$, which follows the ΓΓ distribution as defined in Equation (22) [91,94,96,97]:

(38) $$f_{h_{a}}\left(h_{a}\right)=\frac{2{\left(\alpha \beta \right)}^{(\alpha +\beta )/2}}{\Gamma (\alpha )\Gamma (\beta )}{\left(h_{a}\right)}^{\frac{\left(\alpha +\beta \right)}{2}-1}K_{\alpha -\beta }\left(2\sqrt{\alpha \beta h_{a}}\right),$$

Utilizing the power series expansion of the modified Bessel function around the origin, Equation (38) can be estimated as [241]

(39) $$f_{h_{a}}\left(h_{a}\right)\approx a_{0}I^{\beta -1}+a_{1}I^{\beta },$$

In Equation (39), only the initial two terms of the power series expansion are taken into account, with $a_{0}$ and $a_{1}$ defined accordingly as [241]

(40a) $$\begin{array}{cc} a_{0}= & \frac{\Gamma (\alpha -\beta )}{\Gamma (\alpha )\Gamma (\beta )}{\left(\alpha \beta \right)}^{\beta }, \end{array}$$

(40b) $$\begin{array}{cc} a_{1}= & \frac{\Gamma (\alpha -\beta )}{\Gamma (\alpha )\Gamma (\beta )}\frac{{\left(\alpha \beta \right)}^{\beta +1}}{\left(1-\alpha +\beta \right)} \end{array}$$

All the expressions provided for ΓΓ are valid for point Rxs, where the diameter of the Rx lens (*D*) is zero. Specifically, the channel parameters α and β in Equation (24a) and (24b), as well as the power series expansions in (39), (40a), and (40b), apply in the case of D=0. For Rxs that utilize aperture averaging, where D>0, there is a reduction in the variance in intensity fluctuations. In such cases, the ΓΓ statistical model remains applicable but with modified parameters α and β [233,241].

The effect of aperture averaging is that Equations (39), (40a) and (40b) are no longer valid. For D>0, the parameter β may exceed the parameter α, which is different from the situation with point Rxs. Nonetheless, this issue can be readily addressed, as when β>α, Equation (39) can be reformulated as

(41) $$f_{\gamma \gamma }\left(I\right)\approx a_{0}I^{\alpha -1}+a_{1}I^{\alpha }$$

where (40a) and (40b) can be expressed as

(42a) $$\begin{array}{cc} a_{0}= & \frac{\Gamma (\beta -\alpha )}{\Gamma (\alpha )\Gamma (\beta )}{\left(\alpha \beta \right)}^{\alpha }, \end{array}$$

(42b) $$\begin{array}{cc} a_{1}= & \frac{\Gamma (\beta -\alpha )}{\Gamma (\alpha )\Gamma (\beta )}\frac{{\left(\alpha \beta \right)}^{\alpha +1}}{\left(1-\beta +\alpha \right)} \end{array}$$

It is important to mention that these expressions are akin to (39), (40a), and (40b), where the roles of α and β should be reversed when β>α.

Furthermore, examine the scintillation index, $\sigma_{N}^{2}\left(D\right)$, associated with a Rx lens of diameter *D*. The impact of aperture averaging is to reduce this variance when D>0. Thus, the effectiveness of aperture averaging is influenced by the propagation conditions [233]. The degree of intensity fluctuation is dependent on the size of the Rx aperture [235]. The extent to which aperture averaging diminishes the variance in intensity fluctuations is measured by a parameter known as the *aperture averaging factor* [242]. The aperture averaging factor, denoted as $A_{av}$, is described as the ratio of the normalized intensity variance in fluctuations for a Rx with a diameter *D* compared to that of a point Rx, and is given by [233,235]

(43) $$A_{av}=\frac{\sigma_{N}^{2}\left(D\right)}{\sigma_{N}^{2}\left(0\right)},$$

where $\sigma_{N}^{2}\left(0\right)$ represents the scintillation index for a point Rx with D=0. In the regime of weak fluctuations, *A* can be estimated as [233,235]

(44) $$A_{av}\approx {\left[1+1.062\left(\frac{D^{2}}{4}\frac{k}{L}\right)\right]}^{-7/6}.$$

The concept of aperture averaging to mitigate scintillation effects has been extensively studied both in theoretical research and practical applications [232,234,243]. The reduction in fading is commonly measured by the aperture averaging factor (refer to Equation (43)). Research indicates that a significant reduction in scintillation can be achieved, particularly under conditions of strong turbulence. Enhanced scintillation reduction might be possible by employing multiple beams at the Tx and/or multiple lenses at the Rx [233,244,245].

Additionally, the quest for a distribution that can precisely model the PDF of irradiance data across all AT conditions while considering aperture averaging continues to inspire significant research efforts [238]. To tackle this challenge, an alternative family of PDFs is presented to represent the PDF of received optical power in FSO communications, specifically the Weibull and EW distributions [46,246]. Notably, the EW distribution demonstrates a remarkable fit to both simulation and experimental data across all aperture averaging scenarios, including weak and moderate turbulence regimes, and point-like apertures as well. Another appealing aspect of these distributions is the straightforward closed-form expressions for their corresponding PDF and cumulative distribution function (CDF) [238,247,248,249,250,251].

### 4.6. Spatial Diversity

Spatial diversity is a key technique employed in FSO systems to enhance communication reliability and performance. By utilizing multiple transmission and/or reception points, spatial diversity effectively mitigates the effects of AT, scattering, and signal fading, which are prevalent challenges in FSO communications. This section explores the principles of spatial diversity, its implementation strategies, and the associated benefits, including improved signal quality and increased system robustness. Through a detailed examination of various spatial diversity configurations, we aim to highlight their significance in achieving more resilient FSO links, particularly in dynamic and unpredictable environmental conditions.

#### 4.6.1. Overview

It has been shown that the performance of SISO FSO links is significantly impacted by strong turbulence, falling short of the typical BER targets for FSO applications within practical SNR ranges. This highlights the need for the implementation of robust fading-mitigation techniques [252]. Historically, various techniques have been employed in FSO systems to address the detrimental effects of the atmospheric channel. These include error control coding combined with interleaving [165,253] and multiple-symbol detection through MLSD [47,212]. However, both methods present certain practical challenges [252]. For example, relying solely on an ECC is not an effective strategy for mitigating fades, as it necessitates a significantly large interleaver, around 100 Gb for a 10 Gb/s channel [245,254,255]. Moreover, when the link functions as the PHY layer of a data network, the upper layer protocols struggle to perform optimally in the presence of these fades and the prolonged delays associated with interleaving and deinterleaving, resulting in reduced throughput and poor efficiency [233,254]. It has also been noted that channel coding is effective primarily in weak turbulence conditions or when a large aperture size is utilized. In reality, coding is not efficient in countering fading. Its advantages become more pronounced only in low fading channels or when the effects of turbulence are significantly mitigated, for instance, through techniques like aperture averaging, AO, or spatial diversity [233]. Conversely, MLSD is hindered by high computational complexity [47,252].

Another promising approach is the implementation of spatial diversity, a well-established technique in RF systems. By utilizing multiple apertures at either the Tx or Rx, spatial diversity can significantly enhance performance by introducing additional DoF in the spatial dimension. This method is particularly appealing for reducing the frequency and duration of signal fades [256]. It is especially critical in strong turbulence channels, where SISO links demonstrate extremely poor performance (i.e., exceeding $10^{-3}$ in the SNR range of 30–50 dB) [252]. Furthermore, spatial diversity minimizes the likelihood of temporal blockage of laser beams caused by obstructions, enabling communication over longer distances even in challenging weather conditions [252].

Furthermore, unlike spatial diversity in wireless systems, spatial diversity in atmospheric optical systems can be implemented compactly, as the coherence length is typically on the order of centimeters. This means that multiple Txs or Rxs can be positioned just centimeters apart to experience nearly independent channel fades [245,254,255]. Consequently, effective fading reduction can be achieved through spatial diversity techniques, such as employing multiple beams at the Tx (MISO) [257,258], using multiple apertures at the Rx (SIMO) [233,245,259], or utilizing a combination of both (MIMO) [244,260,261]. Among these approaches, using multiple lenses at the Rx is the simplest, offering a balance between gain and system complexity in achieving spatial diversity [233,262].

**Figure 11:**
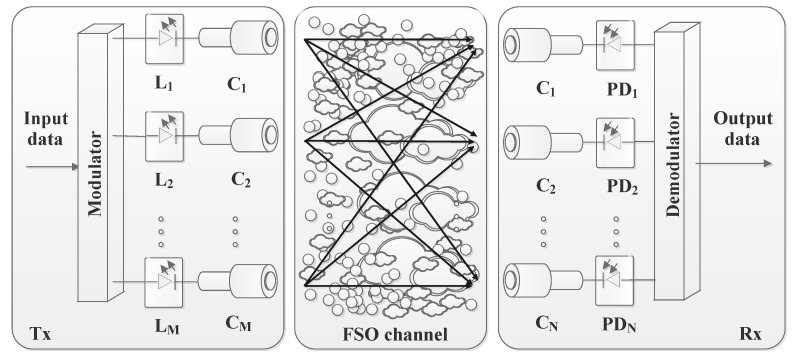
Generic spatial diversity setup.

The overall spatial diversity system configuration, illustrated in Figure 11, consists of *M* Txs and *N* Rxs, where M,N≥1 and at least one of *M* or *N* is greater than one. The outputs from the Rxs can be combined in various ways to generate the final observations, which are used to determine whether a 0 bit or a 1 bit is transmitted [91,255]. In various application scenarios, it is reasonable to assume that the Tx subapertures are spaced apart by more than a coherence length, which is particularly significant for ground-to-space links [254,255].

FSO links utilizing transmit and receive diversity can be effectively modeled as equivalent SISO systems by appropriately scaling the channel variance. In essence, spatial diversity leads to a reduction in channel variance. Additionally, the performance degradation resulting from spatial correlation can be significant, highlighting the importance of efficient separation between apertures and precise co-alignment to realize the expected diversity benefits from multiple Txs and Rxs [263]. A key observation is that as the degree of diversity—whether spatial, temporal, or frequency—increases, performance improves significantly and rapidly [47,254].

#### 4.6.2. MISO, SIMO, and MIMO Free-Space Optical Systems

To improve the reliability and performance of FSO communication systems under varying atmospheric conditions, advanced configurations such as Multiple-Input Single-Output (MISO), SIMO, and MIMO are employed. This section delves into these configurations, exploring their architectures, benefits, and the unique challenges they address in the realm of FSO communications. By leveraging multiple Txs and/or Rxs, these systems aim to alleviate the impacts of AT and enhance link robustness and capacity.

##### Transmit Diversity FSO Systems

Utilizing optical arrays, akin to antenna-array technology in microwave systems, is viewed as a strategy for addressing the fading effects of FSO channels. In particular, MISO (transmit diversity) FSO systems are based on spatial diversity methods with multiple lasers at the Tx end, all of which are pointed towards a distant photodetector. In these systems, the sources and the detector are positioned such that the Rx can simultaneously observe all Txs [264]. Also, this diversity technique has been used to improve PLS in FSO systems [60].

In FSO systems utilizing IM/DD, heuristic STC designs, including RC and orthogonal space–time block codes (OSTBCs), have been suggested as simple alternatives to the MISO FSO diversity scheme [265,266,267]. In the RC system, the same information signal is simultaneously transmitted from all the transmit apertures (laser sources). This is highly effective for reducing fading at the Rx [1,263], and the superiority in RC performance has been shown in [268]. For instance, research indicates that the performance of Alamouti code is inferior to that of RC over FSO links [266,269,270]. Additionally, it has been demonstrated that OSTBC yields worse performance than RC in FSO systems utilizing IM/DD and OOK. Furthermore, studies have shown that RC outperforms OSTBC in a MIMO FSO system using SIM [268,269,271].

Additionally, when CSI is accessible at the Tx, a transmit diversity technique can be employed that selects the optical path with the highest scintillation value. Under IM/DD, this selection transmit diversity (STD) method, also referred to as transmit laser selection (TLS) can achieve full diversity while outperforming other alternatives, such as STCs and RCs. These alternatives implement transmit diversity without relying on CSI, meaning they do not require any feedback from the Rx to the Tx [264,272].

In scenarios where the Tx has imperfect CSI, various transmission strategies are explored by proposing an extension of the STD scheme to situations where a feedback with a limited number of bits is available [272]. In this scheme, the transmit apertures are divided into various partition sizes, and one partition is selected for signal transmission based on feedback bits, utilizing RC. However, optimizing the partition selection is quite complex and necessitates the numerical solution of an infinite integral [273]. Additionally, more intricate signaling schemes can be employed to enhance coding gain alongside the benefits of diversity. For example, a combination of STD and space–time trellis coding (STTC) for MISO FSO communication systems using IM/DD over strong AT channels is analyzed in [274]. Additionally, since a feedback-based beamforming scheme enhances channel-adaptive signaling in wireless communication systems and significantly improves performance, an imperfect quantized feedback-based beamforming approach for a generalized MISO FSO system operating over ΓΓ fading channels with PE is proposed in [275]. Similarly, a simplified version of the STD scheme, which utilizes only one-bit feedback regarding the instantaneous CSI available at the Tx in a MIMO FSO system using SIM BPSK and functioning under ΓΓ fading, is introduced to mitigate the high sensitivity to feedback errors associated with the STD method [273].

The influence of MISO relay-assisted FSO systems on diversity order is being examined using the STD scheme, specifically in the context of ΓΓ fading channels that include PE. The findings indicate that the gain in diversity order is significantly influenced by the position of the relay. Furthermore, it is noted that this gain can significantly diminish when PEs affect the diversity order [276].

##### Receive Diversity FSO Systems

A straightforward approach to mitigate the fading effect is to employ a larger lens at the Rx to average out intensity fluctuations. This method, commonly referred to as aperture averaging, can be regarded as a form of inherent receive diversity. Efficient fading reduction can also be achieved by utilizing multiple apertures at the Rx (SIMO). Specifically, instead of employing a single large aperture, several smaller apertures can be deployed at the Rx. This configuration allows each Rx aperture to experience some degree of aperture averaging, which is less than that of a single large aperture. Moreover, this approach benefits from spatial diversity by combining the signals from the different apertures. Assuming that the signals from the various apertures are subject to uncorrelated fading, the multiple aperture solution outperforms the single large aperture solution, given that both configurations have the same total effective aperture area. It is important to note that using multiple apertures is particularly advantageous in strong turbulence conditions [1,233]. Also, it is important to recognize that, from a practical perspective, utilizing an excessively large lens requires a photodetector with a larger active area to effectively capture the received photons at the lens’s focal plane. This, in turn, significantly constrains the system’s data rate due to the relatively high parasitic capacitance associated with such a component [1].

In SIMO (Receive Diversity) FSO systems, diversity gain can be achieved by averaging multiple independent laser beams. At the receiving end, three techniques can be utilized: MRC, SC, and EGC. Among these, the MRC scheme provides a higher diversity gain, resulting in an optimal signal-to-interference-plus-noise ratio (SINR). In contrast, EGC is often favored over the other two methods due to its simplicity and lower installation costs [245,263,277,278,279,280].

##### MIMO FSO Systems

A highly effective approach to address the dispersive nature of FSO channels involves utilizing multiple lasers and photodetectors, similar to the RF MIMO wireless communication systems. This configuration can enhance channel capacity linearly, scaling with the number of antennas [47,148,281]. MIMO FSO systems are linked to either RC [265,268] or TLS [264,272]. In the case of RC, the same information symbol is sent from all apertures, allowing for implementation without CSI or feedback. Conversely, the TLS approach involves activating the laser that provides the highest irradiance at the Rx, necessitating both a feedback link and the acquisition of CSI [241].

MIMO FSO systems are particularly adept at mitigating AT fading by averaging its effects, thereby increasing the reliability of FSO links [91,282]. Furthermore, the arrangement of multiple sources and detectors can indirectly assist with pointing issues. For instance, a horizontal rooftop transmit/receive array is likely to experience cyclostationary fading rather than complete pointing loss in the event of significant sway [265,283].

In a MIMO FSO system, the laser sources and photodetectors are arranged such that the fading encountered between source–detector pairs is statistically independent, allowing for the exploitation of diversity benefits from the MIMO channel [148,244,265]. Additionally, because of the incoherence of the lasers across the array, their powers combine effectively at the Rx array. In practice, however, MIMO FSO systems may not consistently deliver the theoretical gains they promise. This limitation arises in situations where the assumption of independent channel fading across all links in the MIMO FSO system fails, often due to the proximity of the devices [244,265]. In this context, the limited distance between adjacent optical sources at the Tx or neighboring photodetectors at the Rx can easily lead to spatially correlated fading, which reduces the efficiency of the MIMO FSO system [284,285]. Also, because both the path loss and fading statistics of the channel depend on distance, a significant number of transmit and/or receive apertures is necessary for long-range links to attain the desired performance improvements. This requirement increases the complexity of MIMO FSO systems [244].

The performance of MIMO FSO systems has been explored in various previous studies. For instance, the BER performance of FSO links under LN AT with spatial diversity is examined in [263]. In [286], the study investigates MIMO FSO systems operating in strong turbulence channels, utilizing a single ΓΓ approximation for the distribution of the sum of independent ΓΓ variates and employing EGC at the Rx. Additionally, ref. [287] presents infinite series representations for the error performance of MIMO FSO systems over independent and identically distributed (i.i.d.) ΓΓ fading channels. Furthermore, in [288], the α−μ distribution is employed to approximate the sum of arbitrary i.i.d. ΓΓ variates. This approach leads to simple yet accurate approximations for the OP and average bit error probability (ABEP) of MIMO FSO systems that operate over i.i.d. ΓΓ channels with EGC at the Rx.

For MIMO-FSO systems operating over correlated ΓΓ channels, numerous studies have utilized a simplified channel correlation model, such as the exponential correlation model [263,289]. However, this exponential correlation model, originally developed for RF communications, is inadequate for accurately describing the correlated AT channels. Consequently, recent research has focused on exploring the characteristics of fading correlation induced by AT [258,290,291]. Specifically, studies [258,290] employ wave optics and Monte Carlo simulations to elucidate the effects of turbulence strength, link span, aperture diameter, and separation on channel correlation for both receive and transmit diversity systems. However, since simulation methods can only focus on specific configurations, extending the results to general cases can be challenging. To address this issue, the correlation coefficient as a function of the aforementioned parameters is presented in [291]. In [292], the average achievable rate (AAR) of spatial diversity MIMO-FSO systems over correlated ΓΓ fading channels with arbitrary correlation is examined. The AAR over LN fading channels is considered in [293,294], both with and without bandwidth constraints, respectively. Additionally, ref. [295] investigates the AAR for SM and spatial diversity MIMO FSO links with PE over LN channels.

### 4.7. Error Correction Codes and Modulated Signal Optimization

ECC are crucial in reducing the impact of signal degradation in FSO communication. By detecting and correcting errors in transmitted data, ECC improves the reliability and robustness of FSO links, ensuring consistent data transmission even in challenging atmospheric conditions. However, Forward Error Correction (FEC) alone cannot meet the capacity demands of optical wireless systems. Incorporating high-order modulations, such as PAM and QAM, can enhance channel capacity. Despite this, a gap still exists between the Shannon limit and the capacity of uniform symbols. This section presents advanced schemes designed to enhance communication reliability and efficiency. It also outlines the essential requirements that transceivers must meet to approach channel capacity. We explore ECC, which improves data integrity by detecting and correcting errors. CM techniques are introduced to increase SE by combining coding and modulation processes. Lastly, we delve into capacity-approaching Probabilistic Amplitude Shaping (PAS), a cutting-edge method that optimizes data transmission rates close to the channel capacity limit. Each scheme contributes uniquely to advancing communication technologies, ensuring robust and high-performance data transfer.

#### 4.7.1. Requirements for Channel Capacity-Approaching Transceivers

To develop transceivers that approach the channel capacity in terms of transmission rate, three key requirements must be met. First, the symbol distribution should align with the channel’s capacity-achieving distribution. Second, optimal, adequately long channel codes are necessary. Third, the transmission rate must be finely adjusted according to the channel conditions, meaning the encoder should support a wide range of transmission modes across various SNRs. However, designing efficient CM systems that meet these criteria is challenging. Typically, the input distribution does not match the capacity-achieving distribution, resulting in a shaping gap. Additionally, practical implementations use finite-length Forward Error Correction (FEC) codes, leading to a coding gap. Lastly, the number of modulation orders and coding rates available is restricted by system complexity, resulting in only partially adaptive systems [296,297,298].

Concerning the shaping gap, optimizing the shape of the modulated signal constellation can reduce the discrepancy between the Shannon’s limit and transmission rate. The primary methods of constellation shaping are geometric shaping and probabilistic shaping (PS). In geometric shaping, the constellation symbols are equiprobable but unevenly spaced. Conversely, a probabilistically shaped constellation features uniformly spaced symbols with differing probabilities assigned to each symbol. The latter has garnered significant attention in recent years owing to its rate adaptability, higher shaping gain, and the feasibility of employing Gray code for symbol labeling [296,297,299].

#### 4.7.2. Coded Modulation and Error Correction Codes

As previously mentioned, the performance of an FSO system regarding availability and reliability is greatly influenced by unpredictable variations resulting from AT, which is the primary factor hindering transmission performance [300]. Higher laser powers are essential to extend transmission distances. However, the free-space, optical IM/DD channel presents modem designers with unique challenges. Most practical FSO systems utilize LEDs or LDs as Txs, along with PIN PDs or APDs as Rxs, creating a relatively straightforward communication system. These devices are designed to modulate and detect only the intensity of the carrier wave, rather than its phase, meaning that all transmitted signal intensities must be non-negative. Additionally, considerations for biological safety (specifically eye safety) restrict the average optical power emitted, which in turn restricts the average signal amplitude. The effects of multipath distortion in signal propagation, along with the limited response times of optoelectronic components, impose significant restrictions on the channel bandwidth. Standard signal-space models and CM techniques for electrical channels cannot be directly applied to this scenario because they do not account for the constraints on signal amplitude [301,302]. Consequently, there is a need for power-efficient modulation schemes [148].

As highlighted in Section 4.3, modulation schemes like PAM and QAM, often employed in wireless communications, offer high SE but lack power efficiency, making them less suitable for certain applications. In contrast, *Q*-ary PPM presents a compelling power-efficient alternative for FSO links [244,265,303,304]. The significant bandwidth of FSO links, when contrasted with RF links, mitigates concerns regarding the low SE of PPM [302]. Additionally, as the number of slots in *Q*-ary PPM increases, the impact of background radiation diminishes, largely due to the shorter PD integration interval [244,265]. To enhance the performance of FSO systems utilizing PPM, various FEC strategies have been introduced, such as convolutional codes, Reed–Solomon (RS) codes, turbo codes, and trellis-CM (TCM) [302,305,306]. While RS and convolutional codes provide adequate coding gains in conditions of weak turbulence and without fog, their effectiveness declines under strong turbulence or in deep fog scenarios. In such cases, more sophisticated FEC solutions, including turbo and LDPC codes, become necessary [302,307]. A power-efficient CM scheme employing multilevel coding with LDPC codes as component codes has been proposed for implementation in repetition MIMO FSO systems with *Q*-ary PPM [148].

Moreover, FEC techniques, such as polar codes and LDPC codes, are extensively utilized in FSO to counteract turbulence, achieving substantial coding gains under varying turbulence conditions [164,186,307,308]. For instance, LDPC-coded optical OFDM has been shown to vastly outperform LDPC-coded OOK in AT channels in terms of both SE and coding gain. In strong turbulence conditions at a BER of $10^{-5}$, the enhancement in coding gain of the LDPC-coded single-side band unclipped-OFDM system with 64 subcarriers exceeds that of the LDPC-coded OOK system by 20.2 dB for QPSK and by 23.4 dB for BPSK [186]. Additionally, the combination of FEC and spatial diversity has been demonstrated to mitigate fading [186,213,285]. The LDPC-coded MIMO FSO communication system, which employs LDPC codes and spatial diversity reception to combat turbulence fading when sub-channels are independent, has been examined [186]. In practice, deterioration due to spatially correlated fading is considerable, especially in moderate to strong turbulence channels [290]. Polar codes, a novel FEC technique, are the only ones proven to achieve the Shannon limit with low encoding and decoding complexity and have been selected for the 5G channel coding standard because of their distinctive benefits [309,310]. Polar-coded MIMO-FSO systems with spatially correlated fading using OOK modulations are introduced in [285], where polar codes outperform LDPC codes under the same conditions. However, OOK modulations cannot satisfy the capacity demands of future optical communication. Unipolar M-ary PAM (M-PAM) is a candidate for achieving near capacity in FSO systems [311], and the non-uniform distributions of M-PAM can significantly increase the capacity of FSO systems [297,312].

Furthermore, efficient FSO communications can be realized by integrating higher-order modulation schemes with channel coding, a method referred to in the literature as CM [217,313]. This approach improves system reliability by combining high-order modulations with FEC codes and offers flexible data rates through adjustments in modulation schemes or code rates [164,308].

#### 4.7.3. Probabilistic Shaping

PS with non-uniform probabilities has been shown to reduce capacity gaps and facilitate fine-grained rate adaptation [296]. This approach has become a significant topic in optical communications. By adjusting the signal probability distribution, PS can modify the entropy and improve system performance [314]. Additionally, it can be used in bit-interleaved CM (BICM) systems utilizing PAS, whose advantages have been experimentally confirmed [308,314].

PAS integrates CM with PS [296,315]. This technique has been utilized in coherent communications to enhance SNR in AWGN channels, providing greater flexibility and precise control over bitrate. Recently, time-adaptive PAS has been presented for FSO links, leading to significant capacity improvements under time-varying power fading conditions [316,317]. Nevertheless, the PAS cannot be directly applied to FSO communications because it relies on the symmetry of bipolar signals, whereas FSO systems use unipolar (asymmetric) signals. To address this limitation, the modified Maxwell–Boltzmann distribution based on PAS (PAS-MMB) can be utilized; however, its distribution remains pairwise, which is not ideal for unipolar symbols [309,311]. Additionally, unipolar M-PAM are promising candidates for achieving near-capacity in FSO systems [311]. The non-uniform distributions of M-PAM can further enhance the capacity of these systems. Research in [297,300] has introduced numerical simulations of LDPC and polar-coded PS PAM schemes to optimize the capacity of FSO channels with unipolar signals. In this context, pairwise distributions have proven effective for PAM8, resulting in only minor performance losses [308].

##### Hybrid Constellation Shaping

In addition to PAS, another approach utilizing PS is Sparse–Dense Transmission (SDT). Research indicates that SDT-based OOK modulation can achieve approximately 1 dB of shaping gain in AWGN channels [309]. Furthermore, the capacity can be enhanced through entropy-loading DMT systems based on PS. Entropy-loading is a robust and adaptable method for capacity enhancement, allowing continuous adjustments to the source entropy for each subcarrier. However, implementing entropy-loading DMT presents a trade-off between optimization complexity and achievable capacity [318,319]. In comparison, Hybrid Constellation Shaping (HCS) offers a lower-complexity solution that yields greater shaping gains by combining PS with geometric shaping (GS), particularly for non-Gray mapped QAM [315,320]. Nevertheless, determining appropriate probabilities and geometric shapes to align with existing FEC schemes poses challenges. To address some of the implementation issues associated with HCS designs, a rate-flexible HCS scheme has been presented for DMT-FSO systems [164,321].

##### Bitrate Adaptation Using PCS

PCS is recognized as an effective method to boost system capacity by reducing the energy of the transmitted signal. In AWGN channels, the lowest transmitted signal energy for a specified bitrate is obtained by adjusting the probabilities of QAM symbols to match the Maxwell–Boltzmann distribution [316,322] given as [316,317]

(45) $$P_{x_{n}}=\frac{\exp{\left(-\lambda_{sp}\left|x_{n}\right|\right)}^{2}}{\sum_{n=1}^{M}\exp{\left(-\lambda_{sp}\left|x_{n}\right|\right)}^{2}},$$

where $\lambda_{sp}\geq 0$ denotes the shaping parameter, *M* represents the constellation size of the chosen QAM template for PCS, and $x_{n}$ represents the *n*-th symbol in the square *M*-QAM constellation alphabet, expressed as [317]

(46) $$\begin{array}{cc} x_{n}=\;\; & 2\left(\left(n-1\right)\;\;\mathrm{mod}\;\;\sqrt{M}\right)-\sqrt{M}+1+j\left(2\left\lceil \frac{n}{\sqrt{M}}\right\rceil -\sqrt{M}-1\right). \end{array}$$

In addition to reducing the average signal energy, PCS can also be utilized to adapt the entropy of the transmitted signal, defined as [316]

(47) $$H_{\mathrm{PCS}}=-\sum_{n=1}^{M}P_{x_{n}}\log_{2}\left(P_{x_{n}}\right).$$

Thus, by utilizing Equations (45) and (47), the net bitrate can be adjusted by modifying the shaping parameter $\lambda_{sp}$. Also, in a system utilizing PCS modulation and FEC, the relationship between the system bitrate and the constellation entropy is defined as

(48) $$R_{b}=N_{\mathrm{pol}}R_{S}\left[H_{\mathrm{PCS}}-\left(1-R_{\mathrm{FEC}}\right)\log_{2}M\right],$$

where $R_{b}$ denotes the net bitrate, $N_{\mathrm{pol}}$ represents the number of polarizations, $R_{S}$ denotes the operating symbol rate, and $R_{\mathrm{FEC}}$ denotes the FEC rate.

To accurately set the PCS shaping factor for maximizing channel capacity, it is necessary to first evaluate the achievable information rate for a given SNR. For this purpose, the normalized generalized mutual information (NGMI) is often utilized as a pre-FEC performance metric in systems employing soft-decision FEC and is given by [317]

(49) $$\mathrm{NGMI}=1-\frac{G(M,P_{x},\sigma^{2})}{\log_{2}\left(M\right)},$$

where $\sigma^{2}$ denotes the noise variance in the AWGN channel.

It can be demonstrated that the minimum NGMI required for error-free transmission matches the FEC rate. However, because of the imperfect performance of FEC, it is necessary to consider a slightly higher NGMI threshold, denoted as ${\mathrm{NGMI}}_{\mathrm{th}}$, than the theoretical minimum. To control the transmitted net bitrate, adjust the shaping parameter $\lambda_{sp}$ according to Equation (45).

### 4.8. Cooperative Relaying

In this section, we present relay-assisted transmission as a method to enhance communication reliability and coverage. By utilizing intermediate relay nodes, this technique helps overcome obstacles and extend the range of transmission, ensuring more robust and efficient communication links. We will explore the principles, advantages, and implementation strategies of relay-assisted systems in various communication scenarios.

#### 4.8.1. Relay-Assisted Transmission

In contrast to the localized diversity techniques used in MIMO systems, relay-assisted solutions provide distributed spatial diversity methods, where neighboring nodes (or relays) support communication between a source node and a destination node. Moreover, cooperative communication is a dynamic area of research because of its potential to improve reliability and broaden the reach of wireless networks without the need for a new infrastructure [323]. It has also become a viable method for achieving spatial diversity benefits [324,325,326]. The core concept of cooperative diversity (parallel relaying) is founded on the observation that, in a wireless RF channel, the signal sent by the source node is received by other nodes known as partners or relays. These partners, along with the source, can collaboratively manage and convey their information, forming a virtual antenna array despite each node having only one antenna. Another relay-assisted transmission scheme is multihop transmission (serial relaying), which employs relays arranged in a sequential configuration [327,328,329,330,331]. This approach is typically employed to extend the coverage area for Txs with limited power. It does not enhance performance against fading influences in wireless RF environments, which means it does not improve the diversity order [332]. Also, cooperative techniques are extensively researched in FSO communication systems to counteract the adverse effects of turbulence-induced atmospheric scintillation [323,332,333,334,335,336]. Table 5 presents some of the related work in this area.

Current research in cooperative FSO communications primarily focuses on two approaches: all-active relaying, which is used when CSI is unavailable, and selective relaying, which is applied when CSI is present. All-active relaying is a straightforward and efficient two-slot scheme. In the first slot, the source (S) sends the information packet to the relays (Rs). The relays then decode (or amplify) the received symbols and, in the second slot, forward the processed signals to the destination (D). In this setup, no priority is given to any relay, irrespective of the strengths of the source-relay and relay-destination links [323,332,334,335]. In selective relaying, either direct transmissions occur, or one relay is selected from the pool of available relays to forward the information symbols to the destination based on the channel state [336,337,338]. This approach prioritizes the transmission along the strongest e2e link. Instead of distributing power across multiple relays, the power is concentrated and directed to the *best relay* to improve signal reconstruction quality at this relay. The criteria for selecting the optimal path involve minimizing the conditional error probability for quantum-limited systems without background noise [337], and minimizing the OP for systems with Gaussian noise [336]. While all-active relaying is simple and does not require any CSI, selective relaying provides better performance but comes with increased system complexity. This complexity arises from the need to acquire complete CSI for all intermediate channels and to establish feedback links between the communicating nodes [241,323,339]. Furthermore, it has been observed that the strength of the two-hop source–relay–destination (S-R-D) link depends on its weakest hop. This protocol shows that both all-active and selective relaying methods can achieve full diversity gains, with the selective method offering an improved coding gain. Recently, inter-relay cooperation has been introduced to enhance the reliability of FSO networks, especially in scenarios where FSO links are established between the relays [340].

**Table 5:** Literature on cooperative hybrid FSO.

Link		Channel Model					
**1st** **Hop**	**2nd** **Hop**	**1st** **Hop**	**2nd** **Hop**	**Relaying Mode**	**Detection Technique/Modulation**	**Metrics**	**Reference**
FSO	RF	DGG	EGK	AF, DF	IM/DD, HD	ABER, OP, EC	[13]
RF	FSO	SR	GG	AF	QPSK	SER	[341]
FSO	FSO	NE	NE	AF	IM/DD OOK	ABER, OP	[88]
RF	FSO	Rayleigh	GG	DF	IM/DD, HD	ABER, OP	[342]
RF	FSO	N-*m*	GG	DF	IM/DD OOK	OP	[343]
RF	FSO	Rayleigh	GG	DF	SIM	IC	[344]
RF	FSO	SR	GG	DF, AF	IM/DD OOK	SOP, ASC	[345]
FSO	FSO	GG	α−μ	DF	IM/DD	EC	[346]
FSO	FSO	GG	GG	DF	SIM DBPSK	BER	[347]
RF	FSO	Rayleigh	GG	DF	IM/DD, HD	ABER, OP, EC	[348]
FSO	RF	GG	GN-*m*	AF	IM/DD, HD	ABER, OP, EC	[67]
FSO	RF	GG	GK	AF	SIM	OP	[349]
FSO	RF	EW	N-*m*	DF	IM/DD	OP, ABER	[350]
FSO	RF	M	κ−μ	AF	IM/DD	OP, EC	[351]
RF	FSO	Rayleigh	GG	AF	IM/DD	BER, EC	[352]
FSO	RF	GG	FTR	AF, DF	SIM, IM/DD, HD	OP, BER, EC	[353]
RF	FSO	N-*m*	GG	AF	IM/DD, HD	OP, BER, EC	[354]
RF	FSO	Rayleigh	GG	AF	SIM	OP, BER	[355]
RF	FSO	BX	M	DF	IM/DD, HD	OP, BER, EC	[356]
RF	FSO	Rayleigh	GG	AF	IM/DD	OP, BER, EC	[6]
RF	FSO	α−μ	GG	AF	IM/DD	OP, BER	[357]
RF	FSO	α−μ	DGG	AF	IM/DD	OP, BER, EC	[358]
FSO	RF	α−μ	GG	DF	IM/DD	OP, BER, EC	[359]

SER: Symbol error rate, AF: Amplify-and-forward, DF: Decode-and-forward, QPSK Quadrature Phase-Shift Keying, OOK: on–off keying, DBPSK: differential binary phase-shift keying, NE: negative exponential, GG: Gamma–Gamma Distribution, α−μ: Alpha–Mu Fading, SR: shadowed-Rician fading distribution, N-*m*: Nakagami-*m* fading, FTR: Fluctuating two-ray fading, GN-*m*: Generalized Nakagami-*m* fading, GK: Generalized-K fading, Málaga-M distribution, EW: Exponentiated Weibull fading, BX: Beaulieu–Xie fading, DGG: Double Generalized Gamma Distribution, EGK: Extended Generalized-K fading, OP: Outage probability, SOP: secrecy outage probability, ASC/EC/IC: Average Secrecy/Ergodic/Intercept capacity, BER: bit-error rate, ABER: average bit error rate, IM/DD: intensity-modulation direct detection, HD: heterodyne detection, SIM: subcarrier intensity modulation scheme.

In multi-node scenarios, cooperative communication acts as a unique variant of MIMO by utilizing relay nodes to improve the link quality between the source and destination nodes. This approach not only offers a novel method to boost transmission reliability in FSO systems but also provides a viable solution for FSO networking [332,338,360]. One benefit of this virtual multiple-aperture system, as opposed to MIMO FSO systems, is that the multiple paths are spatially separated, which guarantees the independence of the associated fading channels [336]. Also, compared to the conventional MIMO FSO system, the cooperative FSO system allows for the flexible selection of relay nodes, which helps to diversify transmission paths and effectively eliminate channel correlation. Thus, in cooperative FSO communication systems, data are transmitted through a direct link from a source node to a destination node, as well as through one or more relay nodes. These relay nodes aid in enhancing signal strength and reliability, particularly in adverse atmospheric conditions [284].

Additionally, several cooperative protocols have been suggested for use in relay-assisted FSO links. The traditional methods include amplify-and-forward (AF) [331,332,334,361,362,363,364,365] and DF [331,363] protocols. In AF relaying, optical-to-electrical (OE) conversion is performed at each relay, followed by electrical amplification and modulation of a laser source. The electrical amplifier gain at each relay is established based on the CSI of the previous hop, necessitating continuous estimation of the channel fading amplitude. As with all AF systems, the noise introduced at each relay accumulates along the transmission path, limiting the maximum transmission distance [331,361,363,364,366,367]. DF FSO systems, on the other hand, involve additional electrical processing steps after OE conversion. To mitigate the propagation of background noise, the received electrical signal at each relay is decoded and re-encoded before retransmission in DF systems. While DF significantly enhances performance, it introduces considerable encoding/decoding delays and requires more complex hardware [88,332,366,367,368,369]. The need for more processors results in higher component error rates and increased costs [370,371].

The modified versions of the DF strategy, such as Bit Detect and Forward, Adaptive Bit Detect and Forward, and Adaptive Decode and Forward, have been considered [372]. Additionally, FSO DF relaying techniques have progressed from buffer-free (BF) relaying to buffer-aided (BA) relaying [324,333]. These FSO BA relaying methods are inspired by their RF counterparts [373,374,375]. In mixed RF/FSO systems, buffers play a significant role by queuing data collected from mobile RF users before they are multiplexed along the backhaul FSO link [376]. Implementing BA relay selection strategies requires the acquisition of CSI and/or buffer state information (BSI). CSI knowledge can be either full or partial [373,375,377]. BSI involves acquiring the number of packets stored in the relays’ buffers, which enhances performance but increases system complexity. Recently, deep reinforcement learning (DRL) has been applied to maximize the throughput of RF BA networks, and a relay clustering approach has been suggested to improve performance in asymmetrical RF networks [373,374,375].

#### 4.8.2. Cooperative FSO Transmission Schemes

In this section, we explore the synchronous and asynchronous cooperative FSO transmission schemes. These schemes represent critical strategies for enhancing the performance and reliability of FSO systems. Synchronous cooperative transmission involves the precise coordination of multiple transmitting sources, ensuring that their signals reach the Rx in a well-aligned manner. This alignment can significantly improve signal quality and reduce error rates. Conversely, asynchronous cooperative transmission schemes do not require such strict timing coordination, offering greater flexibility and robustness in environments where perfect synchronization is challenging. By comparing these two approaches, we aim to highlight their respective advantages, limitations, and potential applications in various FSO communication scenarios.

##### Synchronous Transmission Scheme

Synchronous transmission, a fundamental technique, has been utilized since the introduction of cooperative communication in radio systems. In this method, the source node transmits identical data on both the relay links and the direct link, enabling the destination node to combine the received signals synchronously, resulting in a stronger SNR. In this regard, all nodes involved in the cooperative FSO system transmit their signals in a coordinated manner, ensuring that the signals arrive at the destination simultaneously. This coordination requires precise timing synchronization among the source and relay nodes. This approach aims to enhance the reliability of the communication by providing multiple copies of the same signal. The destination node combines these received signals to improve signal quality and mitigate errors caused by AT and path loss. While cooperative communication has been extensively investigated in FSO systems, the redundancy inherent in synchronous transmission persists. Consequently, the FSO system must address the challenge of channel underutilization to achieve spatial diversity through cooperative communication [284].

##### Asynchronous Transmission Scheme

An asynchronous transmission scheme is designed to minimize the substantial link redundancy in cooperative FSO systems (typically seen in synchronous transmission). In contrast to synchronous transmission, the asynchronous scheme enables the source node to send different data over both the direct and relay links, facilitating more flexible and independent data transmission. To leverage the diversity gain from these links, which carry distinct data, a squared signal combination method can be utilized to merge the signals received from multiple links. By jointly processing the signals before and after the squaring operation, the data on each link can be accurately recovered. As a result, asynchronous transmission provides diversity gain for cooperative FSO systems, improves channel utilization, and ultimately increases the effective data rate compared to synchronous transmission [284].

#### 4.8.3. Cooperative FSO System Model

**Figure 12:**
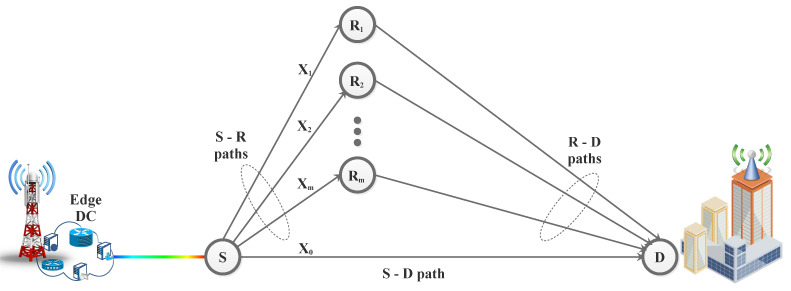
A typical model of a cooperative FSO communication system.

The system model for parallel cooperative FSO communication comprises a source node *S*, multiple relay nodes $R_{i}$, and a destination node *D*. This system features two primary communication links: a direct link from *S* to *D*, enabling *S* to send data directly to *D*, and a relay-assisted link where data are transmitted from *S* to *D* via relay nodes $R_{i}$. The relay node index *i* ranges from 1 to *M*. The relay node employs DF or AF protocols to assist in the transmission. As illustrated in Figure 12, $X_{0}$ denotes the modulated optical signal transmitted directly from *S* to *D*, while $X_{i}$ represents the signal transmitted from *S* to *D* via $R_{i}$.

##### Synchronous Transmission Scheme

In the conventional system model, the optical signal transmitted over each link remains identical, a concept referred to as synchronous transmission. This implies that $X_{0}=X_{1}=X_{2}=\ldots =X_{M}$. Consequently, the received signal after electrical combination in a synchronous transmission system is expressed as [284,360]

(50) $$Y_{sc}=\sum_{i=0}^{M}\left(Y_{i}+N_{i}\right),$$

where $Y_{i}=\bar{g}{\left|X_{i}\right|}^{2}H_{i}G_{i}$ represents the electrical signal received from either the direct link (for i=0) or the relay links (for i=1,…,M); $G_{i}$ represents the gain for optical-to-electrical conversion; $N_{i}$ denotes the AWGN, encompassing thermal noise and background light noise; $|X_{i}{|}^{2}$ is the average optical power of $X_{i}$; and $H_{i}$ denotes the combined channel effect, expressed as

(51) $$H_{i}\overset{\Delta }{=}h_{\ell }h_{a}.$$

The destination node combines the received signals using techniques such as MRC to enhance the signal quality.

##### Asynchronous Transmission Scheme

Unlike the FSO system that uses synchronous transmission, asynchronous scheme allows the source node to transmit different data to each relay node in an uncoordinated manner. In this transmission method, $X_{0}\neq X_{1}\neq \ldots \neq X_{M}$. To recover the data and attain diversity gain from the different links transmitting various data, an electronic signal combiner and a squared signal combination method can be employed at the Rx. This method involves squaring the received signals before combining them. During this process, the *j*-th combined and squared signal (for j=1,…,H) can be mathematically expressed as

(52) $$Z_{j}={\left[\sum_{i=0}^{M}w_{ji}\left(Y_{i}+N_{i}\right)\right]}^{2},$$

where the electrical signal $Y_{i}$ is anticipated to be bipolar with fewer amplitudes after applying the square operator. The coefficient $w_{j,i}$ functions as a relay selection indicator, taking the value of 1 if the relay node $R_{i}$ is chosen to produce $Z_{j}$, and 0 otherwise.

The data on each link can be recovered by jointly processing the signals before and after the square operation, allowing for improved data recovery and reduced redundancy. In this context, the complexity of relay selection can be simplified by employing the pairwise combination method, where each link is supported by another link in pairs for signal combination. The corresponding coefficient matrix can then be denoted as [284]

(53) $$\begin{array}{c} w=\left[\begin{array}{ccc} w_{00} & \cdots & w_{0M}\\ \vdots & \cdots & \vdots \\ w_{M0} & \cdots & w_{MM} \end{array}\right]=\left[\begin{array}{cccccc} 1 & 1 & 0 & 0 & \cdots & 0\\ 0 & 0 & 1 & 1 & 0 & \vdots \\ \vdots & \ddots & \ddots & \ddots & \ddots & 0\\ 0 & 0 & \cdots & 0 & 1 & 1 \end{array}\right]. \end{array}$$

Additionally, a Least Mean Squares algorithm can be used to eliminate the product term of signal and noise from $Z_{j}$. The resulting signal, $Y_{ac1}^{j}$, is given by

(54) $$Y_{ac1}^{j}={\left(\sum_{i=0}^{M}w_{j,i}\cdot Y_{i}\right)}^{2}+{\left(\sum_{i=0}^{M}w_{j,i}\cdot N_{i}\right)}^{2}$$

The square operator improves the SNR of the received signal without increasing its amplitudes. However, it does not allow for the direct determination of the bipolar amplitude (i.e., whether the amplitude is positive or negative) from the squared signal $Y_{ac1}^{j}$. It is essential to recognize that the combined signal prior to squaring retains the bipolarity, which aids in determining the amplitude. Therefore, the merged signal before the square operator can be defined as

(55) $$Y_{ac2}^{j}=\sum_{i=0}^{M}w_{j,i}\cdot \left(Y_{i}+N_{i}\right).$$

In principle, by jointly sampling the signals $Y_{ac1}^{j}$ and $Y_{ac2}^{j}$, the transmitted data can be recovered with a lower BER.

Both synchronous and asynchronous transmission schemes have their advantages and challenges. Synchronous transmission ensures coordinated signal arrival, which simplifies signal processing at the destination node. However, it requires precise timing synchronization, which can be challenging in practical implementations. On the other hand, asynchronous transmission offers more flexibility and reduces link redundancy by allowing independent data transmission on different links. The squared signal combination method in asynchronous transmission provides diversity gain, enhancing data recovery. Consequently, the choice between synchronous and asynchronous transmission schemes in cooperative FSO systems is contingent upon the specific requirements and limitations of the application. Asynchronous transmission, with its reduced redundancy and flexibility, presents a promising approach for enhancing the performance and reliability of FSO communication systems.

#### 4.8.4. Hybrid Satellite–Terrestrial FSO Cooperative System

In [378], it is demonstrated that portable cooperative relaying ground stations can be utilized to offer broadband access in disaster-stricken areas. These ground stations receive signals from a satellite and relay them to a hidden destination node via an FSO link. These hybrid satellite–terrestrial FSO cooperative schemes are not only cost effective but also can be deployed rapidly. In [341], an AF protocol-based hybrid satellite–terrestrial FSO cooperative system is analyzed. In this system, the satellite-to-relay link follows the shadowed-Rician land mobile satellite model, while the relay-to-destination FSO link follows the ΓΓ fading model. This hybrid system is distinct from a typical tandem RF/FSO link, where the RF link follows simpler fading statistics. The moment generating function (m.g.f.) for the hybrid link is derived, and this m.g.f. is used to approximate the average symbol error rate (SER) of the cooperative system. Additionally, the analytical diversity order of the AF-based hybrid cooperative system is determined.

### 4.9. Adaptive Optics

AO is a crucial technology in FSO communication systems, designed to counteract the negative impacts of AT [8,379]. Turbulence can cause phase distortions in the optical wavefront, leading to beam spreading and signal degradation. AO systems utilize real-time feedback to adjust the shape of the optical wavefront, compensating for these distortions and thereby enhancing signal quality [277]. Consequently, through advanced wavefront sensing, real-time correction, and continuous innovation, the reliability and quality of FSO communication links can be improved significantly by AO systems, enabling more widespread and efficient deployment of this promising communication technology. This section explores the principles of AO, its implementation in FSO systems, and the current advancements in AO technology, highlighting its significance in enhancing signal quality and reliability.

#### 4.9.1. Principles of Adaptive Optics

**Figure 13:**
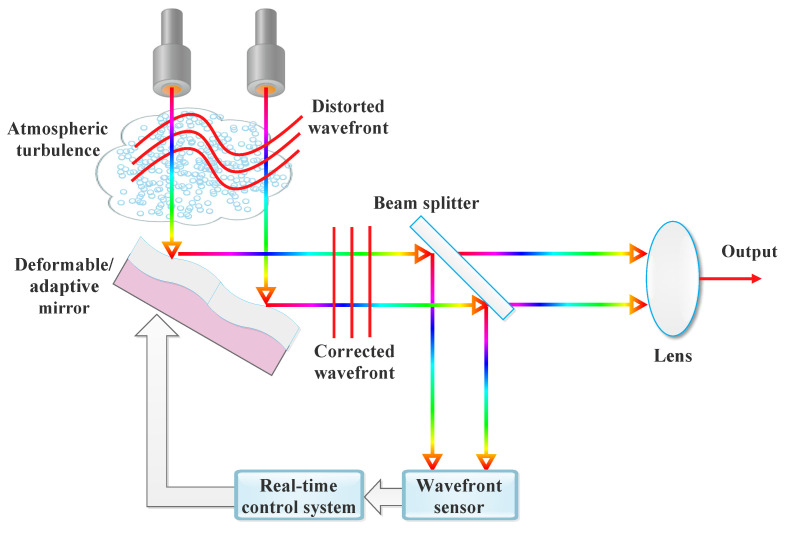
Depiction of an adaptive optics system.

AO systems are designed to correct phase distortions in the optical wavefront caused by AT. These distortions, resulting from variations in air temperature and pressure, can lead to beam spreading, scintillation, and other forms of signal degradation. The application of AO in turbulent conditions mitigates optical wavefront distortion, resulting in notable performance enhancements. Initially developed for astronomy and medical science, AO have gained prominence in imaging, FSO communications, and various military uses. In a turbulent medium, the AO correction process acts as a multiplicative spatial filter on the power spectrum. As defined in [380], AT causes specific aberrations and wavefront distortions, which are statistically characterized. The use of AO for correction is further analyzed through the Zernike polynomials representation in [8,381]. Studies on AO correction with a deformable mirror (DM) as a spatial filter and the associated fitting errors are documented in [382,383]. Research on the outer scale effect on wavefront distortion reveals significant impacts on AO systems [384]. Regarding communication systems, investigations have explored the decrease in the scintillation index for Gaussian beams in non-Kolmogorov turbulent atmospheres and the BER of IM systems with AO [385,386]. These findings collectively demonstrate the efficacy of AO in mitigating turbulence effects. Figure 13 illustrates a simplified AO system from the perspective of FSO. The key principles of AO include the following [277,387]:

(i) Wavefront Sensing:
  - Shack–Hartmann Wavefront Sensor: One of the most common devices used in AO systems is the Shack–Hartmann wavefront sensor. It comprises a series of lenslets that focus portions of the incoming wavefront onto a detector. The displacements of the focal spots on the detector are used to reconstruct the wavefront distortions [387,388,389].
  - Other Sensors: There are various other wavefront sensors, including curvature sensors and pyramid sensors, each with specific advantages depending on the application [390].
(ii) Wavefront Correction:
  - Deformable Mirrors: These mirrors have surfaces that can be precisely shaped using actuators to counteract the detected wavefront distortions. The most commonly used DMs are based on piezoelectric or micro-electro-mechanical systems (MEMS) technology [391,392,393].
  - Spatial Light Modulators (SLMs): These devices modulate the phase of the light passing through them and can also be used for wavefront correction [394,395,396,397,398,399].
(iii) Real-Time Feedback:
  - The AO system continuously monitors the wavefront distortions and adjusts the DM or SLM in real time to compensate for these distortions. This real-time feedback loop is crucial for maintaining high signal quality [277,395,397].

#### 4.9.2. Implementation in FSO Systems

FSO technology has been explored for applications involving both stationary and mobile nodes. In 6G mobile networks, it is anticipated that many antennas utilizing high-frequency bands (such as THz) will be installed on a variety of stationary nodes like traffic lights and signboards, as well as mobile nodes like trains and drones [400]. FSO presents a promising solution to deliver high-capacity mobile fronthaul connections for these antennas [9,12].

In scenarios where FSO is used with moving nodes, an FSO node must adjust its output beam to ensure the FSO signal power surpasses the minimum Rx sensitivity at the receiving node’s aperture [9]. In these FSO communication applications, a significant technical challenge is the PAT. Optical beam steering is a crucial function in PAT [401]. This involves tracking the position of the paired FSO node and adjusting the beam direction accordingly as the paired node moves. The position is monitored using a position detection method, such as a position-sensitive detector. The beam direction is then altered using a rotating mechanism [9]. Traditionally, this has been achieved through mechanical mechanisms such as fast steering mirrors (FSMs), two-axis gimbals, and MEMS mirrors. While effective, these methods are often heavy, bulky, slow to adjust, and prone to mechanical failure [402,403,404,405]. Alternative non-mechanical approaches, such as those using optical phased arrays or liquid crystals, have also been explored. However, optical phased arrays are complicated in terms of control and calibration, and liquid crystals offer a limited steering range. Furthermore, these non-mechanical methods are not yet sufficiently advanced for use in FSO communication systems [401].

Furthermore, to maintain allowable PE, one approach is to use a beam Rx with a larger radius. However, this is challenging to mount on a moving FSO node due to severe constraints on size, weight, and power consumption. Another approach is to utilize a beam with a larger divergence angle at the Tx. Nevertheless, fixing the divergence angle at a larger value reduces the maximum FSO link distance [9]. Recent proposals and demonstrations of adaptive beam divergence control have shown its effectiveness in improving PAT despite PE. By adjusting the beam divergence angle according to channel conditions, this method counteracts the negative impacts of PE. Studies have demonstrated that adaptive beam divergence control can enhance the range of an FSO link and reduce OP in comparison to systems utilizing fixed beam control [9,401].

#### 4.9.3. Pointing, Acquisition, and Tracking

In FSO communication systems, PAT mechanisms can reduce or minimize PE through the continuous monitoring of performance metrics like the Strehl ratio and received signal power. The Strehl ratio is defined as the ratio of the intensity along the optical axis of an aberrated or corrected point spread function (PSF) to the intensity of the diffraction-limited PSF on the optical axis. These metrics help in adjusting correction elements, including mirrors, gimbals, or AO [7].

The PAT mechanism has several key tasks: orienting the Tx towards the Rx, capturing the incoming light signal, and ensuring the FSO link remains stable by monitoring the position of the remote FSO terminal [81]. Pointing involves adjusting the Tx to align with the FoV of the Rx. Signal acquisition refers to aligning the Rx with the direction from which the beam arrives. Tracking entails continuously maintaining both pointing and signal acquisition during the optical communication between terminals [7].

Alongside PAT mechanisms, choosing the right beam divergence can help reduce PE. Specifically, as the beam footprint at the Rx increases, the PE decreases. However, increasing the divergence angle also leads to higher geometric path loss, which is the spread of transmitted power over the communication distance. This loss is influenced by the distance between stations, the divergence angle of the beam, and the size of the Rx aperture. Various optimization models have been proposed to determine the optimal beam width to minimize the BER and OP of an optical wireless link, taking into account PE and SNR [7,23,406,407].

Moreover, due to the increased geometric path loss associated with greater beam divergence, FSO communications typically use a narrow beam with a divergence angle no larger than 1 milliradian (mrad). This narrow beam enhances received power density and boosts link margin under various weather conditions. However, it also necessitates precise alignment between the transceivers due to the beam’s small footprint at the Rx. Achieving this alignment becomes more challenging when at least one of the parties involved in communication is mobile [7,408,409].

PAT mechanisms are typically utilized in stationary and mobile FSO communication systems when employing a narrow beam. The primary motivations for using such beams include the high data rate associated with concentrated light intensity and the extended reach they provide. However, utilizing a wide beam can potentially reduce or even eliminate the performance demands on the PAT mechanism. Certain FSO applications have been proposed to take advantage of a wide beam to ease the requirements of PAT mechanisms. For example, utilizing the divergence angle of a wide beam can reduce the required steering speed of the FSM, a crucial component of the PAT mechanism in an FSO transceiver. It has been shown that a wide beam’s divergence angle offers a greater link range and more effective coverage length compared to a narrow beam, making it advantageous for applications such as high-speed train communications [410].

**Figure 14:**
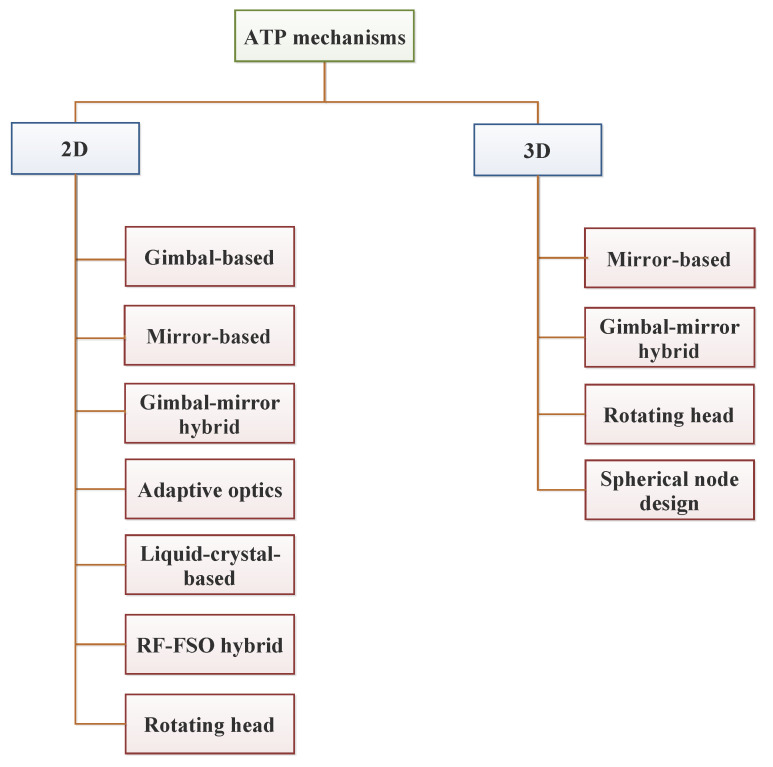
Categorization of PAT mechanisms in FSO communications based on their dimensionality.

PAT mechanisms in FSO communication systems can be categorized based on their principles of operation, including mirror-based, gimbal-based, gimbal–mirror hybrid, liquid crystal, AO, and RF-FSO hybrid methods [411,412]. Additionally, their operating principles and mechanics allow for classification based on dimensionality (i.e., 2D or 3D) as illustrated in Figure 14 [7].

### 4.10. Adaptive Detection Thresholds

This section introduces Adaptive Detection Threshold (ADT) schemes, which are essential for optimizing communication system performance in varying conditions. ADT schemes dynamically adjust detection thresholds based on real-time environmental and signal quality metrics, thereby enhancing reliability and efficiency. By continuously adapting to changing conditions, these schemes mitigate the impact of noise, interference, and signal fading, ensuring robust and efficient data transmission.

#### 4.10.1. Background

In the absence of scintillation, the BER of an FSO system can be improved significantly through the use of an optimal detection threshold based on the maximum likelihood principle, assuming the bit level means and variances are known. However, in an atmospheric channel, turbulence-induced scintillation causes considerable fluctuations in the bit means and variances, which in turn affects the optimal threshold. To address this, a new thresholding method has been developed that replaces the constant threshold with an adaptive one. This method utilizes a Kalman filter algorithm to continuously track and adjust the bit means and variances. In this context, conventional fixed detection thresholds (FDTs) are insufficient for the dynamic environments of FSO systems, as they fail to consider real-time fluctuations in signal quality. Conversely, ADTs improve system performance by dynamically adjusting threshold levels based on the existing channel conditions, thereby optimizing the detection process [413].

Typically, the standard approach for adaptive detection assumes perfect knowledge of the instantaneous CSI, utilizing the instantaneous SNR to detect each data symbol [414,415,416,417]. To ensure reliable communication, adaptive detection techniques can be used to dynamically adjust the Rx sensitivity in response to these fluctuations [418,419,420,421,422]. Consequently, ADT methods have garnered significant interest as effective strategies for alleviating the impacts of AT in FSO. ADT enables systems to attain improved average BER with low complexity [418]. For instance, it is well known that an OOK IM/DD system with a fixed decision techniques leads to an irreducible error floor and is power inefficient. Optimal OOK demodulation necessitates time-varying ADTs based on perfect CSI for detecting each data symbol [194,415,416,421]. However, this approach presents clear practical challenges for OOK IM/DD operation with nanosecond data symbol durations (i.e., Gb/s rates) and millisecond turbulence coherence times. Specifically, it necessitates rapid adjustments to the detection threshold on the millisecond timescale of turbulence coherence times [415,416,423]. In this context, practical FSO systems will not be able to accurately track changes in the means and variances at the bit level. Therefore, optimizing the threshold to a constant value based on slowly varying statistical quantities, such as average signal power and turbulence strength, can also be adopted as a suboptimal method [413,422].

Although the performance of the Rx heavily relies on the chosen threshold level [420], determining the appropriate ADT remains a challenge [418]. Previous studies often assume detection with instantaneous CSI [10,293]. To address these practical issues, an electrical-SNR-optimized detection system has been proposed [211,424]. This system requires changing the detection thresholds only over extended timescales, such as seconds or minutes, during which a stationary turbulence channel assumption is valid [425]. However, this method necessitates knowledge of the turbulence channel’s PDF, thereby increasing computational complexity [414]. For instance, research has indicated that estimating the fading intensity requires using redundant overhead, such as pilot symbols or training sequences [426]. In [424], pilot-symbol assisted modulation (PSAM) and the maximum likelihood method are introduced as methods to reduce the effects of turbulence and improve system performance. However, PSAM can cause delays at the Rx because the entire frame must be stored before decoding. In practice, to simplify commercial implementation and ensure infrastructure transparency, detection without CSI and the PDF of the turbulence channel is the favored approach [194,424].

In [189], the performance enhancements of OOK FSO communication systems under AT conditions are investigated, focusing on the dynamic decision threshold and coherent detection schemes. The findings indicate that the dynamic decision threshold scheme achieves a 5.7 dB reduction in the power needed to reach a BER of $10^{-3}$ in comparison to the conventional FDT approach. Similarly, in [427], it is shown that an OOK IM/DD system can utilize two laser wavelengths at the Tx and two photodetectors at the Rx to operate in differential mode, leading to outstanding BER performance with a detection threshold set to zero. This study is also extended to include PPM. However, this scheme is limited by low throughput, as it requires two lasers to transmit the same information during each symbol duration.

Furthermore, to enhance performance beyond what is achieved in [427], a source information transformation system has been introduced in [414]. This system utilizes multiple optical wavelengths to determine ADTs. It has been demonstrated that this approach can achieve a satisfactory BER performance without needing instantaneous CSI or the PDF of the turbulence model. Numerical simulations indicate that the proposed system delivers performance similar to a theoretical adaptive detection scheme, while significantly reducing implementation complexity. Additionally, it incurs only a 1.8 dB loss in SNR at a BER of $1\times 10^{-9}$ for a lognormal turbulence channel. Also, to enhance threshold accuracy and overall system performance, an FSO communication system using OOK with coherent detection-double ADT has been developed [418]. The Tx employs 3-bit encoding to relay information, while the Rx features two adaptive thresholds to identify the in-phase and quadrature components of the signal, operating without instantaneous CSI or the PDF of the channel model. Additionally, a decision-aided scheme is proposed to establish the second detection threshold, further refining precision. Numerical analyses indicate that this system achieves a BER performance comparable to that of an idealized adaptive detection system.

#### 4.10.2. Mechanism of ADTs

ADTs work by continuously monitoring the received signal’s parameters, such as BER, SNR, and scintillation index. Algorithms, often based on ML or statistical analysis, process these real-time data to determine the optimal threshold. The primary mechanisms include the following:

(i) Feedback Loops: Real-time feedback from the Rx to the Tx helps adjust the power levels and detection thresholds. This feedback loop ensures that the system adapts to varying conditions promptly [194,424].
(ii) Threshold Adjustment Algorithms: Algorithms such as the Kalman filter, maximum likelihood estimation, and adaptive filtering techniques dynamically adjust the detection thresholds. These algorithms analyze the statistical properties of the received signal and make predictive adjustments [413].
(iii) Diversity Techniques: Implementing spatial or temporal diversity techniques can also aid in adaptive threshold adjustment. By combining multiple received signals, the system can better estimate the optimal threshold [423].

#### 4.10.3. Benefits of ADTs

ADTs offer several advantages in FSO communication systems:

(i) Improved Signal Detection: By continuously adjusting to the optimal threshold, the Rx can better distinguish between the signal and noise, leading to lower BER and enhanced data integrity [418,427].
(ii) Enhanced System Reliability: Adaptive thresholds help maintain consistent communication quality even under adverse weather conditions and AT, thereby increasing the system’s reliability [413].
(iii) Efficient Use of Power: Adaptive thresholds can lead to the more efficient use of power by ensuring that the system only uses the necessary amount of power to maintain communication, which is particularly beneficial in power-constrained applications [189,413,422].

#### 4.10.4. Implementation Challenges

Despite their advantages, implementing ADTs in FSO systems comes with challenges:

(i) Complexity: The algorithms required for real-time threshold adjustment can be complex and computationally intensive, necessitating advanced processing capabilities.
(ii) Latency: Real-time adjustments must be swift to be effective. Any latency in the feedback loop or threshold adjustment process can degrade performance.
(iii) Calibration: Adaptive systems require initial calibration and ongoing tuning to ensure they respond appropriately to changing conditions.

### 4.11. Hybrid FSO Systems

The integration of FSO communication with RF, mmWave, and THz technologies has emerged as a promising approach to overcoming the limitations of standalone FSO systems. Hybrid FSO systems leverage the strengths of these technologies to provide enhanced reliability and coverage. This section examines the architecture of hybrid FSO systems, their operational principles, and the benefits they offer in terms of performance and reliability.

#### 4.11.1. Overview

FSO, mmWave, and THz communications have proven to be effective solutions for high-data-rate wireless transmission over short distances. They offer a promising response to the increasing demand for higher data rates amid the growing scarcity of RF spectrum, positioning them as strong candidates for joint deployment to ensure reliable, high-data-rate wireless backhaul [428]. Nevertheless, these technologies fall short of achieving the desired carrier-grade availability of 99.999% due to their related transmission issues. A possible solution to enhance the FSO link’s availability and reliability is to integrate it with the RF (or mmWave/THz) link (hereinafter referred to as an alternative link), which can reach data rates similar to those of an FSO link, to create a hybrid FSO/RF (or mmWave/THz) communication system (hereinafter referred to as a hybrid system) [2,362,365,429]. As a result, a hybrid system is better equipped to maintain connectivity compared to a purely FSO system. By integrating the advantages of various technologies and addressing the limitations of each, the hybrid system greatly improves network performance. It enhances network resilience and offers a backup solution in difficult atmospheric conditions, thereby contributing to overall network reliability [2,365].

#### 4.11.2. Architecture of Hybrid FSO Systems

**Figure 15:**
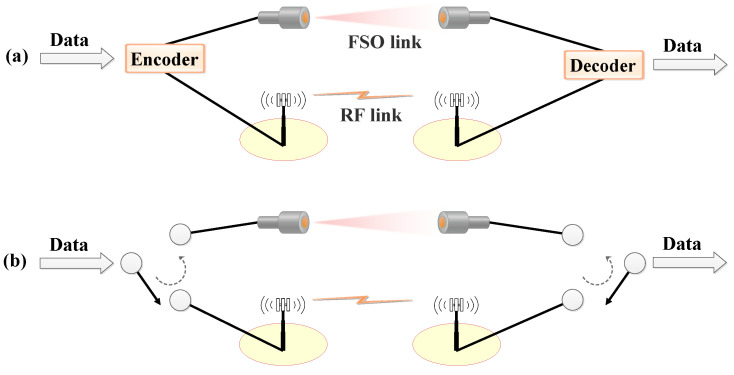
Hybrid FSO switching configurations: (**a**) soft-switching and (**b**) hard-switching.

Hybrid FSO systems typically combine FSO links with RF, mmWave, or THz links to create a robust communication network. In this system, the efficiency and reliability of signal transmission are significantly influenced by the switching configurations employed. This section delves into two primary switching architectures: soft-switching and hard-switching. Soft-switching aims to minimize losses and electromagnetic interference by ensuring smoother transitions between states, thereby enhancing system performance and longevity. Conversely, hard-switching, characterized by abrupt transitions, can lead to increased efficiency under certain conditions but may also introduce greater stress on system components. Understanding the nuances of these configurations is crucial for optimizing FSO systems for various operational scenarios. Figure 15 illustrates two potential setups for a hybrid FSO/RF link.

##### Hard-Switching Configurations

One switching approach is a switch-over hybrid technique that employs hard-switching between the FSO and alternative links to make use of their complementary natures [430,431]. In this approach, as shown in Figure 15a, the Tx and Rx collaboratively decide between the FSO and an alternative channel for data transmission. The FSO link is chosen only when the channel conditions allow for reliable communication; otherwise, all data are transmitted via the alternative channel [432,433]. In this regard, commercially available hybrid solutions simply use the RF link as a hot-standby backup for the FSO link, which is to be used only when the FSO channel is inoperative [434]. It is important to note that the Tx and Rx need to be coordinated through feedback to choose the appropriate channel for transmission. One major drawback of hard-switching is that one link remains idle at any given time. In reality, scintillation or loss in the sensitive FSO channel leads to infrequent selection of that link. Consequently, once the RF link is chosen, the channel capacity of the FSO link goes unused [432]. Also, this method results in frequent switching between the FSO and alternative links [429,430].

##### Soft-Switching Configurations

One solution to address the challenges of hard-switching is to synchronize data transmission across both links using channel coding, as illustrated in Figure 15b. In this method, both the FSO and alternative links are utilized simultaneously for data transmission. One approach involves sending identical data concurrently over both links and employing diversity combining techniques for the received signals from each link [435]. This method, however, limits the system’s data rate to that of the slower link. Alternatively, the data stream can be jointly coded and divided between the two links, potentially leading to a significant enhancement in overall system capacity [431]. In this context, data are encoded using a single LDPC code, with part of the codeword allocated to both FSO and alternative links, and the rate is modified through puncturing based on the current channel conditions [429,432]. While this approach offers improvements over hard-switching, it necessitates knowledge of channel conditions at both the Tx and Rx, as well as complex soft decoding, which poses challenges at FSO data rates. Recently, Raptor codes have also been explored for hybrid FSO/RF links [214,434,436,437]. These codes operate without the need for channel knowledge at the Tx and automatically adjust the rate between RF and FSO links based on channel conditions, utilizing just a single bit of feedback per message [432].

Generally, hard-switching schemes tend to result in some rate loss compared to soft-switching schemes. Both approaches require that the FSO and alternative links remain continuously active, even during periods of poor quality. Consequently, both links must be operational even when the FSO link has good quality and can independently meet the required BER. In such situations, these solutions are inefficient regarding bandwidth utilization and resource usage across both channels [429]. For instance, the power used for alternative link transmission is wasted, leading to unnecessary interference with the environment [428,430]. To tackle these issues of frequent switching, unnecessary RF interference, and RF power wastage, a new hybrid FSO/RF transmission scheme with adaptive combining is proposed in [430]. In this approach, the FSO link is utilized for data transmission as long as its quality remains acceptable, while the RF link is placed in standby mode. When the quality of the FSO link deteriorates, the system activates the RF link and employs MRC for signals received from both the FSO and RF links.

The complementary characteristics of FSO and RF channels have prompted several proposals for hybrid RF/FSO systems. A combination of high-rate FSO with low-rate RF systems operating in the 2.4 GHz frequency band is suggested in [438] and experimentally validated in [433,439]. However, the maximum data rate achievable in such hybrid systems is constrained by the limited RF bandwidth available in the 2.4 GHz band. This limitation can be addressed by utilizing RF systems in the mmWave frequency band, which supports data rates comparable to those of FSO [440,441,442]. When instantaneous CSI is available at the Tx, a straightforward CSI-dependent switching mechanism between the FSO and RF systems can be implemented [438].

Additionally, much of the prior research on hybrid FSO/RF systems has concentrated on the joint design of coding schemes for both FSO and RF links, facilitating soft-switching between the two links [429,434,443]. For instance, to enhance the performance of hybrid FSO/RF systems, ref. [443] employs hybrid channel codes that not only optimally achieve the combined capacity of the FSO and RF channels but also ensure carrier-grade reliability (99.999%) for the FSO link. The coding scheme for the hybrid channel is based on non-uniform punctured LDPC codes. This scheme relies on knowledge of the instantaneous channel conditions at the Tx and the appropriate adjustment of code rates for RF and FSO transmission [428].

An alternative approach to joint FSO/RF channel coding using a practical implementation of rateless Fountain codes is presented in [434]. The scheme belongs to the category of hybrid automatic repeat request (HARQ) that utilizes incremental redundancy coding, and as such does not require rate adjustment prior to transmission. This is a clear distinction relative to rate-adaptive coding techniques like the ones proposed in [429,443]. A bit-interleaved CM scheme for hybrid systems is introduced in [431]. Additionally, [432] proposes the use of short-length Raptor codes. The link availability of hybrid FSO/RF systems is explored from an information theory perspective in [444]. Meanwhile, ref. [435] presents diversity combining for parallel FSO and RF channels, assuming both links transmit identical information simultaneously. The performance of a similar hybrid FSO/RF system subject to non-Gaussian noise is analyzed in [445].

## 5. Emerging Trends in FSO Communication

FSO communication is evolving rapidly, integrating cutting-edge technologies to overcome conventional limitations and enhance performance. This section delves into the latest advancements shaping the future of FSO systems. Key trends include the incorporation of emerging technologies, which bolster system capabilities and adaptability. Enhanced FSO transceiver design aims to improve reliability and efficiency. The integration of quantum cryptography offers unprecedented security for data transmission. OAM-aided FSO systems introduce a novel method for multiplexing, significantly increasing data throughput. Additionally, the application of AI in FSO systems promises to optimize performance through intelligent decision-making and adaptive strategies. Together, these innovations are poised to revolutionize FSO communication, making it a more viable and robust solution for modern communication needs [2].

### 5.1. Incorporation of Emerging Technologies

The landscape of wireless communication is rapidly evolving, fueled by the increasing need for higher data rates, improved reliability, and seamless connectivity. As mentioned in the preceding section, FSO communication, known for its high bandwidth and security, is being integrated with other advanced technologies such as RF, mmWave, and THz systems [2,93]. This section discusses these emerging trends in detail, highlighting how these integrations can enhance communication systems’ overall performance and capabilities.

#### 5.1.1. Hybrid FSO/RF System

FSO and RF technologies have complementary strengths that can be harnessed to overcome the limitations of each. While FSO provides high bandwidth and secure communication, RF is less affected by weather conditions and can penetrate obstacles. This integration enhances reliability and resilience by providing alternative communication paths in adverse conditions. Also, in the hybrid system, RF links can serve as a backup for FSO links during adverse weather conditions (e.g., fog, rain) that degrade optical signals. This redundancy ensures continuous and reliable communication. Likewise, advanced algorithms can switch dynamically between RF and FSO links based on real-time environmental conditions and link quality, optimizing the communication system’s performance. Because of the offered reliability and redundancy, the hybrid FSO/RF system is ideal for providing last-mile connectivity in urban areas where physical obstructions and varying weather conditions pose challenges. Also, integration with RF enables the deployment of FSO links in mobile networks, supporting high-speed data transfer for moving platforms like vehicles and ships. Also, the scheme’s high-speed data transmission capabilities make it a suitable solution for backhaul and fronthaul applications, improving the overall performance of the 5G infrastructure [2,93,365].

#### 5.1.2. Hybrid FSO/mmWave System

mmWave frequencies (30–300 GHz) offer higher bandwidth compared to traditional RF frequencies, supporting gigabit per second (Gbps) data rates. Similar to FSO, mmWave communication utilizes highly directional beams, which can reduce interference and enhance security. So, combining FSO and mmWave technologies can significantly increase the available bandwidth, enabling ultra-high-speed communication. Also, both FSO and mmWave systems benefit from advanced beam steering and alignment techniques, often driven by AI and ML algorithms, to maintain optimal connectivity. Due to the high frequency, mmWave signals are best suited for short-range applications. Integrating them with FSO can extend the range and improve the reliability of these short-range links. Based on this, the scheme is crucial for the deployment of 5G networks and the development of 6G, providing the high data rates and low latency required for these advanced networks [12]. Also, the hybrid system can be used for high-speed data transfer within data centers and for backhaul connections between network nodes [2].

#### 5.1.3. Hybrid FSO/THz System

THz frequencies (0.1–10 THz) offer even higher data rates than mmWave, potentially supporting tens to hundreds of Gbps. The THz spectrum provides ample bandwidth, which is crucial for future high-capacity communication systems. FSO can complement THz communication by providing high-capacity links in scenarios where THz propagation is limited due to atmospheric absorption and scattering. Integrating FSO with THz technologies can leverage advanced modulation techniques to maximize data throughput and SE. AI-driven adaptive systems can dynamically allocate FSO and THz resources based on real-time performance metrics, optimizing the communication link’s quality and capacity. So, the scheme is a key enabler for wireless terabit networks, which are essential to enable future data-intensive applications, including holographic communications and immersive virtual reality. Hybrid FSO/THz systems can enhance space communication links, providing high-speed data transfer between satellites and ground stations or inter-satellite links [2].

In general, the integration of FSO communication with RF, mmWave, and THz technologies represents a notable trend in the evolution of wireless communication systems. These hybrid systems leverage the strengths of each technology to overcome their individual limitations, resulting in enhanced performance, reliability, and capacity. As research and development continue, these integrated systems are expected to be instrumental in the deployment of next-generation networks, supporting a broad spectrum of applications from urban connectivity to space communications. This convergence of technologies represents a significant advancement in realizing the vision of ubiquitous, high-speed wireless communication [2,365].

### 5.2. Enhanced FSO Transceiver Design

A roadmap for future wireless communications is expected to exploit all transmission-suitable spectrum bands, from the microwave to the optical frequencies, to support orders of magnitude faster data transfer with much lower latency than the deployed solutions nowadays. The currently under-exploited mid-infrared (mid-IR) spectrum is an essential building block for such an envisioned all-spectra wireless communication paradigm. FSO communications in the mid-IR region have recently attracted great interest due to their intrinsic merits of low propagation loss and high tolerance of atmospheric perturbations [446].

Meanwhile, FSO technologies have been proposed, studied, and even developed for decades by reusing the fiber-optic components in the near-IR band (1–2 μm). Unfortunately, these have not been considered reliable building blocks of the modern ICT infrastructure. The main concern of these near-IR FSO technologies is that they are susceptible to the dynamic atmospheric environment. For instance, with the development of passive and active fiber components and devices, the 1.55 μm band, also referred to as the telecom band, has been applied for commercial FSO communication systems. However, the 1.55 μm band is sensitive to atmospheric channel perturbations caused by fog, dust, and turbulence [447].

Recently, the under-exploited mid-IR spectral region, particularly the two atmospheric transmission windows at the mid-wavelength IR (MWIR, 3–5 μm) and the long-wavelength IR (LWIR, 9–12 μm) [448], are attracting increasing interest for FSO communications. The interest in a MWIR platform stems from the intrinsic PHY layer advantage of low absorption when propagating in the atmosphere even under adverse conditions (for instance, scattering by aerosols, rain, and snow, as well as beam broadening and scintillation by turbulence effects), robustness of the wavefront during long-distance propagation compared with the near-IR FSO, and the absence of regulations and restrictions for this range of wavelengths [446,448,449,450]

The future development of viable mid-IR FSO transceivers requires a semiconductor source to fulfill the high-bandwidth, low-energy-consumption, and small-footprint requirements. In this context, quantum cascade devices, including quantum cascade lasers (QCLs) and quantum cascade detectors (QCDs) appear as promising technological candidates to fulfill this role [446,447,451,452]. Quantum cascade devices are based on inter-sub-band transitions to cover a wide spectral range from the mid-IR to THz. Particularly, directly modulated-QCLs have ultra-short carrier relaxation lifetime, leading to a high intrinsic modulation bandwidth and making the laser response over-damped, thus repressing the resonance frequency [449,451,452]. It is a promising candidate for solid-state FSO Txs at the MWIR and LWIR bands. Combined with advanced modulation formats (e.g., PAM and DMT) and DSP techniques, capacity-approaching transmission performance in both optical and wireless communication systems can be achieved [447].

### 5.3. Quantum Cryptography

Quantum cryptography represents a groundbreaking advancement in secure communication, utilizing the concepts of quantum mechanics to ensure the confidentiality and integrity of data. When integrated with FSO communication systems, quantum cryptography, particularly Quantum Key Distribution (QKD), offers unparalleled security, providing ultra-secure communication channels, particularly in scenarios requiring high levels of data protection. Also, integrating QKD into FSO involves transmitting qubits via the same optical channel, requiring precise alignment and stable atmospheric conditions to minimize decoherence and loss. So, AT, scattering, and absorption can impact the fidelity of quantum states. Advanced error correction and AO schemes can be used to alleviate these effects. Also, maintaining accurate alignment between the Tx and Rx is crucial. Automated tracking systems and fine-tuning mechanisms help maintain the integrity of the quantum channel [2,453,454,455].

In general, the integration of quantum cryptography with FSO communication systems holds immense potential for revolutionizing secure communications, particularly within the framework of 5G and future networks. By leveraging the principles of quantum mechanics, QKD provides a level of security that is fundamentally unattainable with classical cryptographic methods. As we advance towards more connected and data-driven environments, the role of QKD in ensuring ultra-secure communication channels will become increasingly vital, offering robust solutions across various sectors, from finance and healthcare to government and smart cities [453,454,456].

### 5.4. Orbital Angular Momentum-Aided FSO Systems

Several multiplexing methods, such as WDM, space division multiplexing (SDM), and polarization division multiplexing (PDM), have been employed to enhance the transmission capacity of FSO communication systems. However, the numerous limitations and the complex architecture of FSO Rxs make it challenging to significantly boost the transmission capacity further. Recently, a new multiplexing technique known as OAM has gained prominence among researchers for its potential to further increase the capacity of FSO systems [457]. So, in future ultra-fast optical communication, besides the widely applied characteristics of light, including amplitude, phase, polarization, and wavelength, the OAM will offer a new degree of freedom of light beams. In principle, light beams carrying different OAM states are mutually orthogonal, and the number of OAM eigenstates is infinite. Therefore, the application of OAM provides alternative technologies to meet the ever-increasing bandwidth demands in wireless communication systems [2,458].

Moreover, to enhance the data transmission capacity of FSO communication, techniques utilizing OAM—a fundamental property of Laguerre–Gaussian (LG) beams—have been proposed and garnered significant attention [459]. Leveraging the theoretically unlimited range of available OAM states [460], OAM division multiplexing [461,462] and OAM-shift keying (OAM-SK) [50,463,464] can significantly improve the capacity of FSO communication systems. Specifically, in OAM division multiplexing, LG beams carrying various OAM states act as channels to multiplex information streams [458]. Traditionally, these beams are multiplexed at the Tx and then transmitted through the atmosphere to the Rx, where they are demultiplexed due to their orthogonal properties [465]. Meanwhile, in OAM-SK, information is modulated into LG beams with different OAM modes, representing a new modulation format. The OAM-SK-FSO communication system offers distinct advantages, including reduced equipment costs, increased transmission distances, enhanced photon efficiency, and improved information security. This makes it particularly suitable for cost-effective emergency communication and medium-distance FSO applications, such as high-dimensional QKD and deep-space or near-Earth communications. Additionally, advancements in SLMs can further enhance the capacity of OAM-SK communication systems.

### 5.5. Artificial Intelligence-Driven FSO Systems

The integration of AI and ML into FSO communication systems represents a significant advancement and is a rapidly growing trend, offering new possibilities for optimizing system performance and reliability. AI and ML technologies offer powerful tools to address the inherent challenges of FSO communication, such as atmospheric interference, alignment issues, and error correction. In this regard, AI-driven algorithms can be used to predict atmospheric conditions, estimate channels, modulate and demodulate signals, optimize beam alignment, monitor performance, and enhance error correction processes. For example, ML models can analyze vast amounts of meteorological data to predict weather patterns with high accuracy. By integrating these predictions into FSO systems, operators can anticipate adverse conditions and take preemptive measures [2,404,466,467,468,469,470,471,472,473,474].

Furthermore, AI-driven systems can continuously monitor atmospheric conditions using sensors and adjust system parameters like the transmission power, modulation schemes, and other parameters accordingly in real-time to maintain optimal performance. In this regard, recent advancements in deep learning (DL) have enabled the development of sophisticated models for processing FSO signals. These models can filter out noise, enhance signal clarity, and improve data rates [467]. For example, FSO communication systems utilizing DL for various roles—including channel estimation, detection, joint constellation shaping, and as a combined channel estimator and detector—are discussed in [475]. In [476], a design of an optical feedback network employing ML techniques is presented, demonstrating its effectiveness in correcting the effects of turbulent propagation on optical modes. This artificial neural network (ANN) approach relies solely on measuring the intensity profile of the distorted modes, making it both simple and robust. Additionally, a novel joint AT detection and adaptive demodulation technique utilizing convolutional neural networks (CNNs) is proposed to enhance the reliability and flexibility of OAM-FSO communication [469]. An *m*-ary adaptive demodulator based on the maximum likelihood for light beams carrying OAM over FSO turbulence channels has also been proposed and demonstrated [458]. Also, in [477], a new turbo-coded 16-ary OAM-SK-FSO communication system is introduced, which incorporates a CNN-based adaptive demodulator designed for operation under strong AT. In [465], a method is proposed for demultiplexing OAM-carrying beams by capturing an image of their unique multiplexing intensity pattern and training a CNN to serve as a classifier. This CNN-based approach simplifies the demultiplexing process, as it eliminates the need for alignment, relaxes orthogonality constraints, and reduces the reliance on expensive optical hardware.

AI is being used to manage and optimize entire FSO networks. This includes tasks such as traffic routing, load balancing, and resource allocation, ensuring efficient and reliable network performance. The research presented in [474,478] utilizes the actor–critic method from reinforcement learning for power adaptation in SM FSO systems. Additionally, the study in [466] addresses the broader issue of resource allocation aimed at reducing channel fading effects in FSO communications. This resource allocation problem is framed within a constrained stochastic optimization framework, encompassing a range of FSO scenarios that involve power adaptation, relay selection, and their combined allocation. In a similar vein, ref. [479] explores optimal resource allocation in FSO fronthaul networks. This optimal allocation aims to maximize the average weighted sum-capacity while adhering to constraints related to power limitations and data congestion. The study takes into account both adaptive power assignment and node selection, leveraging the instantaneous CSI of the links. By parameterizing the resource allocation policy, the problem is framed as an unsupervised statistical learning challenge. Furthermore, the use of graph neural networks (GNN) for policy parameterization is proposed to effectively utilize the FSO network structure with a reduced number of training parameters.

Maintaining precise alignment between the Tx and Rx is crucial for FSO communication. AI algorithms can process data from various sensors to automatically adjust the alignment of the optical beam [480]. This includes compensating for environmental factors such as wind-induced vibrations or the movement of mobile platforms. ML models can predict the best alignment strategies based on historical data and real-time feedback, ensuring the beam remains accurately directed towards the Rx even in dynamic conditions [467,481]. For instance, sensor-less AO is among the most promising techniques for compensating significant wavefront disturbances in FSO. A backpropagation ANN can be utilized in sensor-less AO systems to create a distortion correction scheme. This approach requires only one or a few online measurements to correct wavefront distortion, unlike other model-based methods, thereby enhancing the system’s real-time capabilities [473].

One of the mostly used DL algorithms in optical communication applications is a deep neural network (DNN). The DNN is an extension of the ANN [482]. It is flexible and could be used for various applications, such as modulation format identification [483,484], mitigating the fiber effects [485], optical amplifier control [486], optical performance monitoring [50,487], along with various optical network applications such as quality of transmission (lightpaths) prediction [488] and an AI-driven routing framework for multi-domain optical networks [489]. For instance, a novel block-wise detection technique utilizing support vector machines (SVMs) to address scintillation noise in FSO links is proposed in [471]. Given that turbulence fading occurs on a millisecond scale, while signaling rates typically reach hundreds of Mbps, the SVM approach offers an exceptionally large data block proportional to the bit rates. Additionally, the SVM calculation is independent of the channel’s turbulence conditions and the received signal models, requiring no prior information or heuristic assumptions. Furthermore, ref. [490] introduces a new detection method for OOK-modulated signals using various DL models across different strengths of FSO turbulent channels, without needing prior knowledge of the channel parameters. The DL decoders enhance the performance of the FSO turbulent channel and reduce power consumption. Notably, these DL models also operate faster than maximum likelihood methods with perfect channel estimation, achieving slightly better performance under turbulent conditions, thus facilitating FSO communications over turbulent atmospheric channels.

In general, AI-driven FSO systems could enable autonomous vehicles, drones, and other robotic platforms to communicate seamlessly, even in challenging environments. This includes applications in disaster response, remote sensing, and smart cities. AI and ML could play a critical impact in the development of quantum FSO systems, optimizing the transmission and reception of quantum signals for ultra-secure communication. Also, AI-aided FSO systems can lead to the development of smart infrastructure, where buildings, streetlights, and other urban elements are equipped with FSO transceivers managed by AI, providing ubiquitous connectivity [2,458,469,491]. In this context, AI-driven FSO communication systems are transforming the field, offering innovative solutions to long-standing challenges and paving the way for new applications. By leveraging AI-driven algorithms for predicting atmospheric conditions, optimizing beam alignment, and enhancing error correction, FSO systems can achieve higher performance and reliability. As research and development in this area continue to advance, the potential applications of AI and ML in FSO communication are vast, promising a future where seamless, high-speed optical communication is a reality [466,479,481,492].

## 6. Open Challenges, Future Prospects and Research Directions

FSO communication systems have developed into an innovative technology for high-speed wireless communication. Despite their potential, various challenges need to be overcome to fully harness their capabilities. This section explores the key open challenges, discusses future prospects, and outlines potential research directions in FSO communication. By identifying critical areas, such as AT mitigation, aperture averaging effects, and advanced modulation techniques, we aim to offer an in-depth overview of the current landscape, fostering a deeper understanding of where focused efforts and innovative solutions are needed. This discussion is crucial for guiding ongoing and future research, ensuring continued advancement and addressing the critical needs of the field.

### 6.1. FSO Scalability and Reliability for 5G and Beyond Networks

The scalability and reliability of FSO systems are critical factors for their adoption in next-generation networks. As the world transitions to 5G and beyond, the demand for high-capacity, low-latency, and resilient communication networks is skyrocketing. This section assesses the potential of FSO to scale with the increasing demands of 5G and beyond, addressing key issues such as network architecture, deployment strategies, resource management, and system integration.

#### 6.1.1. Network Architecture

The architecture of a network determines its ability to scale and adapt to new demands. In the realm of FSO systems, several architectural considerations are crucial. FSO systems can complement existing RF networks by providing high-capacity backhaul links. Hybrid architectures that seamlessly integrate FSO with RF can offer improved redundancy, resilience, and bandwidth. Also, implementing mesh network topologies with multiple FSO nodes can enhance coverage and provide alternative pathways for data transmission, improving reliability and scalability.

Distributed control mechanisms can enhance the scalability of FSO networks by enabling more efficient resource management and reducing the load on central controllers. Also, integrating edge computing capabilities with FSO nodes can reduce latency and improve the performance of latency-sensitive applications, which is critical for 5G and beyond [478,493].

#### 6.1.2. Deployment Strategies

Effective deployment strategies are essential for the successful scaling of FSO systems. In this regard, site selection and planning are very important. For example, thorough LoS analysis is required to identify optimal locations for FSO transceivers, minimizing the impact of physical obstructions. Also, installing FSO equipment on rooftops, towers, and other elevated structures can enhance coverage and reduce the likelihood of obstructions.

Additionally, FSO can be effectively used for backhauling small cell networks in dense urban areas, where laying fiber may be impractical or cost prohibitive. High-density deployment of FSO nodes can support the massive device connectivity required for 5G and beyond, providing high-capacity links in congested areas. Also, deploying mobile FSO units on vehicles or drones can provide temporary high-capacity links in emergency situations or during large events, offering flexibility and rapid deployment capabilities.

#### 6.1.3. Resource Management

Effective resource management is essential for the scalability and reliability of FSO systems. Dynamic resource allocation is an important aspect of resource management. In this regard, implementing AMC schemes can optimize the use of available bandwidth and maintain link quality under varying atmospheric conditions. Likewise, dynamic load balancing across multiple FSO links can enhance network performance and prevent congestion.

Utilizing WDM technology can significantly increase the capacity of FSO links by transmitting multiple data streams on different wavelengths over a single optical path. Efficient channel allocation strategies can minimize interference and maximize the utilization of available optical spectrum.

Furthermore, priority-based traffic management can facilitate network performance. For example, implementing QoS mechanisms that prioritize critical traffic can ensure reliable performance for latency-sensitive applications, such as real-time video and Internet of Things (IoT) services. Similarly, advanced error correction techniques can alleviate the impact of signal deterioration caused by atmospheric conditions, enhancing the reliability of FSO links.

#### 6.1.4. System Integration

Integrating FSO systems with existing network infrastructure and emerging technologies is vital for scalability. Key integration consideration include compatibility with existing networks. So, ensuring interoperability between FSO systems and existing network equipment, such as routers and switches, can facilitate seamless integration and operation. Also, adopting standardized protocols and interfaces can simplify the integration process and enhance compatibility across different vendors and technologies.

In addition, for effective integration with 5G and beyond technologies, FSO systems must be designed to support the requirements of 5G and future networks, including high data rates, ultra-low latency, and massive connectivity. Also, integrating FSO with IoT infrastructure and smart city applications can provide high-capacity, low-latency links for different use cases, such as smart lighting, traffic management, and environmental monitoring. Implementing centralized network management systems can provide real-time monitoring and control of FSO links, facilitating proactive maintenance and optimization. Also, leveraging AI and ML techniques can enhance network management by predicting and mitigating issues related to atmospheric conditions, traffic patterns, and potential obstructions.

In general, the potential of FSO communication systems to scale with the increasing demands of 5G and beyond is promising, provided that key challenges related to network architecture, deployment strategies, resource management, and system integration are addressed. By leveraging hybrid network architectures, adopting efficient deployment strategies, implementing dynamic resource management techniques, and ensuring seamless integration with existing and emerging technologies, FSO can be crucial in the next generation of high-capacity, reliable communication networks.

### 6.2. Standardization and Regulatory Challenges

For FSO communication to achieve widespread adoption, it is essential to establish standardized protocols and address regulatory challenges. This section discusses the current state of FSO standardization efforts and the regulatory issues that need to be resolved to facilitate the deployment of FSO systems on a global scale. By analyzing the current state of FSO standardization, this section highlights the progress made, the challenges remaining, and the significance of these efforts in facilitating the growth and integration of FSO systems into global communication networks.

#### 6.2.1. Current State of FSO Standardization Efforts

The standardization of FSO systems is crucial for ensuring interoperability, reliability, and widespread adoption. This section explores the ongoing efforts to develop and implement standards for FSO technology. It examines the roles of various international bodies and industry groups in establishing guidelines and protocols that address key aspects such as performance metrics, safety regulations, and compatibility with the existing communication infrastructure.

##### IEEE Standards

The Institute of Electrical and Electronics Engineers (IEEE) has been instrumental in developing standards for various communication technologies. For FSO, the IEEE 802.15.7-2018 standard focuses on OWCs, including FSO. This standard outlines the PHY layer and medium access control (MAC) layer specifications for short-range OWC, which can be adapted for FSO applications [494,495,496,497]. IEEE is also working on extending these standards to accommodate long-range FSO communication, which is crucial for bridging the gap between current capabilities and future needs.

##### ITU Recommendations

It is important to note that the International Telecommunication Union (ITU) has recognized the potential of FSO systems, and through the ITU—Telecommunications Standardization Sector (ITU-T), it has approved Recommendation G.640 [498], providing a procedure for enabling the interference-free coexistence of more than one p2p FSO communication system at a location. The Radio Communication Sector of ITU (ITU-R) has also issued a report on fixed service use cases using FSO links, defining device characteristics, possible applications, as well as technical and operational aspects of FSO systems [499]. Also, ITU has developed recommendations that cover aspects of optical communication, including FSO. ITU-T G.9991, for instance, deals with high-speed indoor OWC transceivers, which can inform FSO developments [500,501,502]. ITU-R P.1817 provides guidelines for the performance and availability of optical free-space links, addressing atmospheric effects and link budgets. These recommendations are critical for ensuring reliable FSO communication across different environments [503,504]. The recommendation in [505] provides propagation prediction methods for planning terrestrial FSO systems. It includes methods to estimate attenuation in clear air, fog, and rain and snow precipitation. It also covers scintillation and impairments by sunlight.

#### 6.2.2. Regulatory Issues

The deployment of FSO communication systems faces several regulatory barriers that must be resolved to guarantee widespread adoption. Key issues include spectrum allocation, compliance with international standards, and the establishment of safety guidelines to prevent interference with other communication systems and ensure eye safety. This section explores these regulatory hurdles, discusses current policies and their implications, and outlines the necessary steps for creating a supportive regulatory framework that can facilitate the efficient and safe deployment of FSO systems.

##### Spectrum Allocation

Unlike RF communication, which operates within regulated frequency bands, FSO communication utilizes unlicensed optical spectrum. While this provides a significant advantage in terms of deployment flexibility, it also raises concerns about potential interference and the need for global harmonization. Also, regulatory bodies need to establish guidelines for the use of optical spectrum, ensuring that FSO systems can operate without interference from other optical devices and across national borders.

##### Safety Regulations

FSO communication involves high-powered lasers, which can pose safety risks to humans and animals if not properly regulated. The International Electrotechnical Commission (IEC) provides standards such as IEC 60825-1, refs. [3,506] which outlines safety requirements for laser products. Compliance with laser safety standards is critical for the safe deployment of FSO systems. Regulatory bodies must enforce these standards and conduct regular inspections to prevent accidents and ensure public safety.

##### Deployment Permits and Zoning Laws

Installing FSO equipment, especially in urban areas, requires navigating complex zoning laws and obtaining necessary permits. Local regulations can vary significantly, impacting the speed and cost of FSO deployment. Streamlining the permitting process and creating uniform regulations can facilitate faster and more efficient deployment of FSO systems, particularly in densely populated regions.

##### Cross-Border Coordination

For international FSO links, especially those connecting different countries, coordination between national regulatory bodies is essential. Issues such as signal interference, spectrum management, and compliance with safety standards must be harmonized to ensure seamless cross-border communication. International cooperation and agreements are necessary to address these challenges, fostering a regulatory environment conducive to global FSO deployment.

##### Environmental Regulations

The deployment of FSO systems must also consider environmental regulations, particularly in sensitive areas such as wildlife reserves or historical sites. Ensuring that FSO infrastructure does not negatively impact the environment is a key regulatory concern. Regulatory frameworks should include provisions for environmental impact assessments and the implementation of mitigation measures to protect ecological and cultural heritage.

Moreover, to realize the full potential of FSO communication, stakeholders must collaborate to establish comprehensive standards and regulatory frameworks. By addressing these standardization and regulatory challenges, the FSO communication industry can pave the way for widespread adoption, enabling high-speed, secure, and reliable wireless communication on a global scale.

### 6.3. Open Challenges

Despite the significant advancements in FSO technology, several open challenges remain that require further research. These include the development of more robust mitigation techniques for AT, the integration of FSO with emerging technologies like Light Fidelity (Li-Fi), and the exploration of new materials and devices for FSO systems. This section identifies the key areas where further future research is needed to fully realize the potential of FSO communication in next-generation networks.

#### 6.3.1. Mitigation Techniques for Atmospheric Turbulence

AT remains one of the primary obstacles in FSO communication, causing signal degradation and fluctuations in signal strength. Future research should focus on developing advanced AO systems that can dynamically adjust the optical beam in real-time to compensate for turbulence-induced distortions. Also, enhancing error correction techniques to effectively mitigate the impact of turbulence on data integrity is important. Likewise, extensive research on the utilization of ML algorithms to predict atmospheric conditions and adjust FSO parameters proactively to maintain optimal performance is required. Also, investigating hybrid systems that combine FSO with other technologies, such as RF, to provide redundancy and maintain connectivity in turbulent conditions is important to realize the full potential of FSO communication.

#### 6.3.2. Integration with Emerging Technologies

The integration of FSO communication with emerging technologies like Li-Fi and 5G/6G networks presents exciting opportunities and challenges. This involves exploring the seamless integration of FSO with Li-Fi to enable high-speed, high-capacity indoor and outdoor communication networks. This includes addressing handover issues between indoor Li-Fi and outdoor FSO links [201]. Similarly, it entails investigating the role of FSO in supporting ultra-high-speed data rates and low-latency communication in 5G and future 6G networks. This involves the development of protocols and standards for FSO integration with existing and future wireless networks. This will facilitate the development of FSO-based solutions to support the growing demand for high-capacity, low-latency communication in IoT applications and smart city infrastructures [378,493].

#### 6.3.3. New Materials and Devices

Advancements in materials science and photonic devices are crucial for the evolution of FSO systems. Researching new materials and designs for high-performance photodetectors that offer higher sensitivity, faster response times, and better performance in diverse environmental conditions is essential. Also, developing low-loss optical components, such as lenses, mirrors, and modulators, to improve the efficiency and extend the range of FSO systems is highly imperative, as is investigating the use of quantum dots and other nanomaterials to create novel light sources and detectors that can operate efficiently under varying atmospheric conditions. Another important aspect is creating flexible and wearable FSO devices to expand the application scope of FSO communication in areas like personal area networks and medical devices [446,448,449,450,493,507].

#### 6.3.4. Security Enhancements

Ensuring the security of FSO communication is vital, especially for sensitive applications. Developing advanced encryption techniques tailored for the high data rates and unique characteristics of FSO links is vital to ensure robust FSO systems. Also, researching methods to enhance PLS, such as beamforming and spatial diversity, to protect against eavesdropping and jamming is important, especially in applications for finance and healthcare. In this regard, exploring the use of QKD over FSO links to provide unbreakable encryption keys for secure communication require further research [46,86,453,454,455,493,508,509].

#### 6.3.5. Network Architecture and Protocols

The development of new network architectures and protocols is essential to support the deployment and scalability of FSO systems. In this regard, certain areas such as designing network topologies that can adjust dynamically to varying conditions and optimize FSO link performance require further research: creating efficient routing and handover protocols to manage the transition between FSO and other communication technologies, ensuring seamless connectivity, and leveraging software-defined networking to enable flexible and programmable FSO networks that can be easily managed and optimized for different applications and environments [75,340,478,508,509].

Addressing the previously mentioned key areas of research is essential for realizing the complete capabilities of FSO communication in next-generation networks. By developing robust mitigation techniques for AT, integrating FSO with emerging technologies, exploring new materials and devices, enhancing security, and innovating network architectures and protocols, researchers can pave the way for more reliable, efficient, and versatile FSO communication systems. These advancements will enable FSO to play a pivotal role in meeting the growing need for high-speed, high-capacity wireless communication in the future.

### 6.4. Future Directions

**Figure 16:**
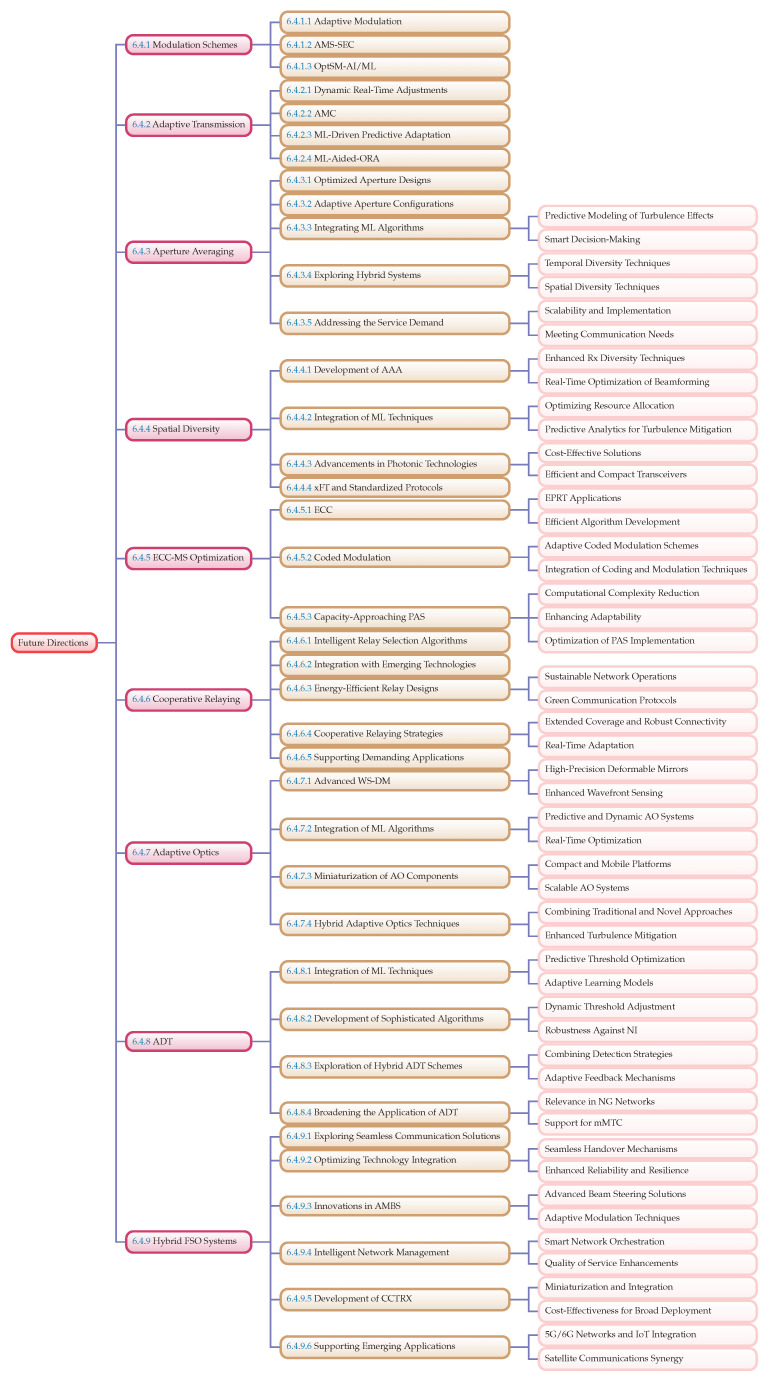
Schematic of the tutorial organization with related content. AMS-SEC: Advanced Modulation Schemes in conjunction with Sophisticated Error Correction, OptSM-AI/ML: Optimized Spatial Modulation with AI and ML, ML-Aided-ORA: ML-Aided Optimization and Resource Allocation, AMC: Adaptive modulation and coding, AAA: Advanced Adaptive Algorithms, ECC-MS: Error Correction Codes and Modulated Signal, EPRT: Enhanced Performance in Real-Time, PAS: Probabilistic Amplitude Shaping, xFT: Extensive Field Trials, WS-DM: Wavefront Sensors and Deformable Mirrors, NI: Noise and Interference, NG: Next-Generation, mMTC: Massive Machine-Type Communications, ADT: Adaptive Detection Thresholds, AMBS: Adaptive Modulation and Beam Steering, CCTRX: Compact and Cost-Effective Transceivers.

This section explores future directions in optical communication technologies, focusing on several key advancements. It delves into novel modulation schemes, adaptive transmission techniques, and aperture averaging methods to enhance signal quality. The discussion extends to spatial diversity and cooperative relaying strategies aimed at improving communication reliability. Additionally, the integration of AO and ADTs is examined for their potential to mitigate atmospheric distortions. The potential of hybrid FSO systems, along with advanced ECC and modulated signal optimization, is also highlighted, underscoring the continuous evolution of this field. The future directions are highlighted in Figure 16 and explored in detail in the following subsections.

#### 6.4.1. Modulation Schemes

The future of FSO communications is poised for considerable advancements through the continuous enhancement and integration of various modulation schemes. These advancements will enable FSO systems to achieve higher capacities, enhanced reliability, and greater resilience against atmospheric challenges. Several key directions in the enhancement and integration of traditional, advanced, and SM techniques promise to significantly improve the performance of FSO communication networks.

##### Adaptive Modulation

Traditional modulation techniques, while foundational, are being complemented and surpassed by more advanced methods. Among these, adaptive modulation stands out. Consequently, adaptive modulation techniques will be essential in future FSO systems. These techniques adjust the modulation parameters in real-time based on the current channel conditions. For instance, during periods of favorable atmospheric conditions, higher-order modulation schemes such as 64-QAM can be used to enhance data rates. Conversely, during periods of high turbulence or fog, the system can switch to more robust modulation schemes like BPSK to maintain link reliability. The dynamic nature of adaptive modulation ensures that FSO systems can optimize their performance under a diverse array of environmental conditions [93].

##### Advanced Modulation Schemes in Conjunction with Sophisticated Error Correction

Advanced error correction techniques are also playing a crucial role. By correcting errors that occur during data transmission, these methods enhance the reliability and efficiency of the systems, even in the presence of atmospheric disturbances like fog, rain, and turbulence. Thus, future modulation schemes will also incorporate advanced error correction methods to alleviate the impact of channel impairments. Techniques such as LDPC codes and Turbo codes will be integrated into the modulation process to detect and correct errors in the transmitted data. These error correction methods will enhance the reliability of FSO communications, ensuring that data are accurately received even amid interference and noise [93].

##### Optimizing Spatial Modulation with AI and ML

SM techniques, which exploit multiple spatial channels, are proving essential in increasing data rates and system capacity without requiring additional bandwidth. Also, AI and ML are emerging as transformative technologies in this domain. The application of AI and ML algorithms can further optimize SM schemes. AI and ML can analyze large volumes of data to predict channel conditions and dynamically adjust modulation parameters in real-time. For instance, ML models can predict periods of high turbulence and preemptively switch to more robust modulation schemes, reducing the risk of data loss. These predictive capabilities will ensure that FSO systems maintain optimal performance in real-time, enhancing their overall efficiency and reliability.

#### 6.4.2. Adaptive Transmission

Future research on adaptive transmission schemes for FSO communication systems promises to bring substantial advancements to the field. In this context, adaptive transmission schemes in FSO communication systems are crucial for overcoming the inherent challenges posed by atmospheric conditions and ensuring robust, high-quality data transmission. So, as FSO technology advances, several promising directions for future research and development can significantly advance adaptive transmission techniques, enhancing their reliability, efficiency, and overall performance.

##### Dynamic Real-Time Adjustments

One primary focus is the development of more sophisticated algorithms capable of dynamically adjusting to real-time channel conditions. These algorithms will leverage advanced signal processing techniques to continuously monitor and respond to changes in the atmospheric environment, such as fog, rain, or turbulence. By doing so, they will ensure that the communication link remains robust and efficient, regardless of external conditions. In this regard, future research will likely focus on the creation of more sophisticated algorithms capable of making dynamic adjustments to transmission parameters in real-time. These algorithms will analyze the present condition of the communication channel, including factors such as AT, alignment errors, and environmental changes, and adjust modulation schemes, power levels, beam steering, and/or other transmission parameters accordingly, all performed in real-time. By doing so, they will maintain optimal communication performance and minimize errors and data loss.

##### Adaptive Modulation and Coding

Advances in AMC techniques will be pivotal. Algorithms that can switch between different modulation formats and ECC based on real-time channel assessments will enhance the resilience of FSO systems. For instance, during periods of high turbulence, the system could switch to a more robust modulation scheme to ensure data integrity, while reverting to higher data rate schemes when conditions are favorable.

##### ML-Driven Predictive Adaptation

The integration of ML and AI techniques represents another promising direction. These technologies can be used to develop predictive adaptation methods that preemptively mitigate the effects of adverse conditions. In this context, ML techniques hold significant promise for enhancing adaptive transmission in FSO systems. By leveraging vast amounts of historical and real-time data, ML models can predict atmospheric conditions and channel states, allowing FSO systems to preemptively adjust their transmission parameters. Predictive adaptation allows the system to prepare for potential disruptions before they occur and can mitigate adverse effects such as signal fading and scattering, ensuring a more stable and reliable communication link.

##### ML-Aided Optimization and Resource Allocation

ML algorithms can also optimize resource allocation within FSO networks. By analyzing usage patterns and channel conditions, these algorithms can allocate bandwidth and power resources more efficiently, enhancing overall network performance. For example, during periods of peak demand, the system could dynamically allocate more resources to critical communication links, ensuring consistent service quality.

#### 6.4.3. Aperture Averaging

As FSO communication systems advance, exploring new developments in aperture averaging offers several promising directions that can significantly enhance system performance. Aperture averaging, which mitigates the impact of AT by averaging the received optical signal over a larger aperture, is critical for improving signal quality and reliability in FSO systems. Future research in this area can explore several innovative approaches to optimize and advance aperture averaging techniques.

##### Optimized Aperture Designs

One promising direction is the optimization of aperture designs. By refining the size, shape, and configuration of optical apertures, researchers can further reduce the impacts of turbulence and enhance the quality of the received signal. In this regard, they can identify designs that minimize the scattering and diffraction of optical signals as they travel through the atmosphere. Optimized aperture designs can effectively average out the scintillations caused by atmospheric variations, leading to more stable and reliable communication links. This can involve the use of novel materials and engineering approaches to create apertures that are more efficient and effective in mitigating turbulence.

##### Adaptive Aperture Configurations

Innovations in AO and dynamic aperture configurations also hold significant potential for real-time performance enhancement. AO systems, which use DMs and other technologies to adjust the optical path in real-time, can be integrated with aperture averaging to dynamically counteract turbulence effects. This real-time adjustment capability allows the FSO system to maintain high performance under varying environmental conditions. Thus, dynamic aperture configurations, where the aperture size, shape and/or arrangement can be altered on-the-fly, can also adapt to changing turbulence conditions. This adaptability will help maintain signal integrity and reduce errors in data transmission.

##### Integrating Machine Learning Algorithms

The integration of ML algorithms into aperture averaging techniques is another exciting avenue for future research. ML can be employed to develop predictive models that anticipate the effects of AT. Also, ML can also facilitate more intelligent decision-making within FSO systems [400].

Predictive Modeling of Turbulence Effects: The integration of ML algorithms into aperture averaging techniques can revolutionize how FSO systems handle atmospheric disturbances. ML models can analyze historical and real-time data to predict turbulence patterns and their effects on optical signals. By employing predictive modeling, FSO systems can proactively adjust their aperture configurations or signal processing parameters, enhancing resilience against turbulence-related challenges.

Smart Decision-Making: ML can also enable smarter decision-making within FSO systems. For example, algorithms could assess the current atmospheric conditions and determine the optimal aperture configuration or modulation technique to employ, ensuring that the system adapts to real-time changes. This capability will improve link reliability and enhance overall system performance, particularly in dynamic environments where conditions fluctuate rapidly.

##### Exploring Hybrid Systems

Investigating hybrid systems that integrate aperture averaging with other diversity techniques—such as spatial or temporal diversity—holds promise for further improving FSO communication. By integrating these diversity techniques with aperture averaging, researchers can create hybrid systems that offer enhanced resilience against turbulence and other atmospheric disturbances.

Spatial Diversity Techniques: By leveraging the strengths of various approaches, hybrid systems can provide enhanced link reliability and greater overall communication efficiency. For example, spatial diversity techniques can be combined with aperture averaging to exploit multiple paths for signal transmission, mitigating the effect of turbulence and improving the robustness of the communication link.

Temporal Diversity Techniques: Temporal diversity, which involves transmitting signals over different time slots, can also be effectively integrated with aperture averaging. By employing adaptive transmission schemes that adjust the timing of signal transmission based on atmospheric conditions, FSO systems can minimize the risk of signal degradation. This combination could lead to a more resilient system designed to sustain high data rates even under challenging weather conditions.

##### Addressing the Service Demand

Advancements in aperture averaging will be pivotal in meeting the high-capacity demands of 5G and future networks.

Meeting Communication Needs: As the demand for high-capacity optical communication solutions grows—driven by applications such as 5G, remote sensing, and satellite communications—the advancements in aperture averaging will play a crucial role. Enhanced signal quality and link reliability achieved through optimized aperture designs and innovative adaptive techniques will ensure that FSO systems can support the increasing data traffic and diverse application requirements in both urban and remote settings.

Scalability and Implementation: Future research should also focus on ensuring that these advancements in aperture averaging are scalable and practical for deployment in various environments. Developing cost-effective solutions and robust implementation strategies will be vital for the widespread adoption of FSO systems. This includes creating standardized protocols for the design and deployment of aperture averaging techniques, ensuring interoperability across different platforms and applications.

#### 6.4.4. Spatial Diversity

Spatial diversity techniques in FSO communication, specifically MISO, SIMO, and MIMO systems, are becoming increasingly essential in addressing the difficulties presented by AT and signal degradation. Future research in these areas is set to explore several key avenues aimed at enhancing performance, reliability, and applicability of FSO systems.

##### Development of Advanced Adaptive Algorithms

Adaptive approaches will greatly improve link reliability and data throughput, especially in dynamic environments, where the atmospheric conditions can vary rapidly.

Real-Time Optimization of Beamforming: One of the promising research directions is the development of advanced adaptive algorithms that optimize beamforming and Rx diversity in real-time. By flexibly adjusting the transmission and reception parameters depending on current environmental conditions, these algorithms can significantly improve link reliability and quality. For instance, algorithms that can assess atmospheric conditions—such as turbulence and scatter—will allow for intelligent adjustments in beam alignment and power levels, ensuring that the optical signal remains robust even in challenging situations. Also, adaptive beamforming techniques can direct the optical beams in real-time to the most favorable paths.

Enhanced Rx Diversity Techniques: In addition to beamforming, enhancing Rx diversity techniques is critical for mitigating the effects of multipath propagation caused by atmospheric disturbances. Rx diversity algorithms can combine signals from multiple Rxs to maximize signal quality. Future algorithms may incorporate feedback mechanisms that evaluate signal quality across multiple Rxs, allowing systems to select the optimal Rx in real-time. Such adaptive diversity strategies will ensure a more stable communication link and minimize the potential for signal dropouts.

##### Integration of Machine Learning Techniques

Integrating ML techniques into FSO communication offers another exciting research avenue for spatial diversity systems.

Predictive Analytics for Turbulence Mitigation: Integrating ML techniques into spatial diversity systems presents a significant opportunity for improved performance. ML models can analyze vast amounts of historical and real-time data to predict turbulence patterns and their potential impact on signal quality. By leveraging predictive analytics, FSO systems can preemptively adjust transmission parameters, such as modulation schemes and power levels, thereby enhancing turbulence mitigation strategies and resource allocation.

Optimizing Resource Allocation: ML can assist in resource allocation by determining the most efficient use of available communication channels and transceivers based on real-time and historical data. This predictive capability can greatly improve the resilience and efficiency of FSO systems. For instance, ML can play a pivotal role in optimizing resource allocation in MIMO systems. By intelligently managing the distribution of available bandwidth and power across multiple transmission paths, these algorithms can enhance throughput and efficiency, especially in dense urban environments where interference is prevalent. The ability to dynamically allocate resources based on real-time conditions will be crucial for maintaining high-quality communication links.

##### Advancements in Photonic Technologies

Advancements in photonic technologies and materials will be essential for the development of more efficient, compact, and cost-effective transceivers in spatial diversity systems. This can lead to the development of FSO systems that are easier to deploy and maintain.

Efficient and Compact Transceivers: Research should focus on miniaturizing components, integrating multiple functionalities into single devices, and improving energy efficiency. Innovations in photonic integration, such as the use of photonic integrated circuits (PICs), have the potential to decrease the size and power consumption of FSO devices while enhancing their performance. These innovative designs will facilitate the creation of lightweight transceivers that can be deployed in a diverse array of applications, from small-scale IoT devices to large-scale urban infrastructure [510].

Cost-Effective Solutions: Reducing the cost of these technologies will be crucial for enabling the widespread adoption of advanced spatial diversity systems. By streamlining manufacturing processes and exploring new materials that offer improved performance at lower costs, researchers can ensure that these technologies are accessible for various applications, including broadband access in underserved areas. Additionally, new materials with superior optical properties can improve the efficiency and reliability of FSO transceivers.

##### Extensive Field Trials and Standardized Protocols

Extensive field trials are essential not only for the widespread adoption of advanced FSO systems but also for validating the performance of advanced spatial diversity systems and their associated new technologies and algorithms in real-world environments. This process will provide essential data for further refinement to enhance system performance. In this regard, the trials will provide valuable insights into system behavior under various atmospheric conditions and help refine algorithms and protocols for optimal performance. By conducting tests in diverse settings—rural, urban, and coastal—researchers can assess the adaptability and reliability of FSO systems across different scenarios.

#### 6.4.5. Error Correction Codes and Modulated Signal Optimization

The future directions for ECC and modulated signal optimization in FSO communication are promising and diverse, encompassing advancements in algorithm development, integrated coding and modulation techniques, and innovative approaches like capacity-approaching PAS.

##### Error Correction Codes

Research in ECC will likely focus on creating more efficient algorithms capable of handling higher data rates and more complex error patterns, especially in real-time use cases.

Efficient Algorithm Development: The development of these algorithms will be driven by the need to correct errors introduced by AT, scattering, and absorption, which can significantly degrade the quality of FSO links. Future ECC will need to be adaptive, adjusting to the varying error characteristics of the FSO channel in real-time. This could involve ML techniques to predict and correct errors dynamically, thereby improving the efficiency and reliability of data transmission.

Enhanced Performance in Real-Time Applications: As real-time applications like video streaming and online gaming become more prevalent, ECC must evolve to provide low-latency error correction. Research will focus on reducing the computational complexity and latency of these codes while maintaining high error correction performance. This could involve the use of parallel processing techniques and hardware accelerators to achieve faster error correction without compromising the integrity of the data.

##### Coded Modulation

Future work in CM will aim to further integrate coding and modulation techniques. Additionally, efforts will focus on developing adaptive CM schemes.

Integration of Coding and Modulation Techniques: Coding and modulation techniques will be combined to optimize SE and enhance resilience against interference. This integration is crucial for FSO systems, where SE is paramount due to the limited available bandwidth. By combining coding and modulation, these systems can transmit more information over the same bandwidth while enhancing resistance to errors caused by atmospheric disturbances.

Adaptive Coded Modulation Schemes: Future research will likely focus on developing adaptive CM schemes that can adjust to changing channel conditions in real-time. These schemes will use feedback from the Rx to adapt the modulation order and coding rate, optimizing the trade-off between the error resilience and data rate based on current channel conditions. This adaptability will be essential for maintaining high performance in the variable and unpredictable FSO environment.

##### Capacity-Approaching Probabilistic Amplitude Shaping

Future research in capacity-approaching PAS will aim to further optimize PAS implementation, enhance adaptability, and reduce computational complexity. The work will focus on ensuring efficient use of the communication channel by aligning the signal’s amplitude distribution with the channel’s capacity.

Optimization of PAS Implementation: Future research will focus on optimizing PAS implementation in practical systems, ensuring that it can be deployed effectively in real-world FSO networks. This will involve developing efficient algorithms for shaping and decoding that minimize computational complexity while maximizing performance.

Enhancing Adaptability: Efforts will also be directed at enhancing the adaptability of PAS to different channel conditions. This adaptability is crucial for FSO systems, where channel conditions can change rapidly due to weather and other environmental factors. Researchers will work on creating PAS schemes that can dynamically adjust the signal shaping based on real-time feedback from the channel, ensuring optimal performance under all conditions.

Reducing Computational Complexity: Reducing the computational complexity of PAS is another critical area of focus. While PAS offers significant benefits in terms of SE and robustness, its implementation can be computationally intensive. Future research will aim to streamline the algorithms used for PAS, making them more efficient and practical for deployment in FSO systems. This could involve leveraging advanced hardware, such as Field-Programmable Gate Arrays and Graphics Processing Units, to accelerate the necessary computations.

#### 6.4.6. Cooperative Relaying

The future directions of cooperative relaying in FSO communication are centered on enhancing network performance, reliability, and efficiency to address the requirements of increasingly demanding use cases. This involves advancements in relay selection algorithms, integration with emerging technologies, energy-efficient designs, and innovative cooperative relaying strategies.

##### Intelligent Relay Selection Algorithms

One of the key areas of future research is the development of intelligent relay selection algorithms that utilize ML to dynamically optimize the selection of relay nodes based on real-time network conditions. These algorithms can analyze data such as signal quality, node locations, and atmospheric conditions to make informed decisions on the best relay nodes to use at any given time. This dynamic optimization helps to maintain robust communication links, reduce latency, and improve overall network performance. ML techniques like reinforcement learning and neural networks can be particularly effective in predicting and adapting to changing conditions in real-time, ensuring optimal relay performance.

##### Integration with Emerging Technologies

Future research also focuses on integrating relay-assisted transmission with innovative technologies like 5G and future advancements. This integration will enable ultra-reliable low-latency communication (uRLLC) and massive machine-type communications (mMTC), which are crucial for applications like autonomous vehicles, smart cities, and industrial automation. By combining the low-latency and high data rates of 5G with the robust, high-capacity links provided by FSO, these hybrid systems can deliver seamless and reliable connectivity. This approach also supports the dense deployment of IoT devices and ensures that critical applications maintain connectivity even in challenging environments.

##### Energy-Efficient Relay Designs

Energy efficiency is gaining increasing importance in communication networks. Consequently, future research is expected to concentrate on exploring sustainable network operations and the development of green communication protocols.

Sustainable Network Operations: Future research will explore energy-efficient relay designs to extend network coverage and improve energy sustainability. This includes the development of low-power relays and the utilization of renewable energy sources like solar power for relay nodes. Energy-efficient algorithms that minimize power consumption while maintaining high performance will be critical for ensuring the sustainability of large-scale FSO networks. These advancements are crucial for lowering the carbon footprint of communication networks and for deploying FSO systems in remote or off-grid locations [511,512].

Green Communication Protocols: The development of green communication protocols that prioritize energy efficiency without compromising performance will be another focus area. These protocols can manage the power usage of relay nodes and optimize data transmission to reduce energy consumption, contributing to the overall sustainability of the network [511,512].

##### Cooperative Relaying Strategies

Innovative cooperative relaying strategies will be investigated to extend network coverage and enhance connectivity. This will encompass the real-time adaptation of relay nodes.

Extended Coverage and Robust Connectivity: Cooperative relaying involves multiple relay nodes working together to transmit data, which can significantly improve the robustness and reliability of communication links. By leveraging spatial diversity, these strategies can alleviate the impacts of AT and ensure consistent performance across the network. Future research will investigate new cooperative techniques, such as network coding and relay selection diversity, to maximize the benefits of cooperative relaying.

Real-Time Adaptation: The ability of relay nodes to adapt in real-time to changing network conditions will be a key focus. This includes developing protocols that allow relay nodes to switch roles dynamically, share resources, and balance the load efficiently. Real-time adaptation will ensure that the network can handle varying traffic demands and maintain high performance even under challenging conditions.

##### Supporting Demanding Applications

The advancements in cooperative relaying are aimed at supporting increasingly demanding applications such as autonomous vehicles and IoT networks. Autonomous vehicles require ultra-reliable, low-latency communication to ensure safety and efficient operation. Similarly, IoT networks involve a vast number of connected devices that need robust and seamless connectivity. The future research directions in cooperative relaying will ensure that FSO systems can meet these demands, providing high-capacity, reliable, and efficient communication links.

#### 6.4.7. Adaptive Optics

Future research in AO for FSO communication is poised to significantly enhance system reliability and performance by addressing the challenges posed by AT and environmental variability. Key areas of focus include the development of advanced wavefront sensors, DMs, the integration of ML algorithms, miniaturization of AO components, and the exploration of hybrid AO techniques.

##### Advanced Wavefront Sensors and Deformable Mirrors

The development of more advanced wavefront sensors is critical for improving the ability of AO systems to detect and correct for atmospheric distortions in real-time.

Enhanced Wavefront Sensing: Future research will likely explore new sensor technologies that offer higher sensitivity, faster response times, and greater accuracy. Innovations in wavefront sensing could include the use of novel materials and designs that improve performance while reducing power consumption and cost. These sensors will be essential for detecting subtle changes in the optical wavefront caused by turbulence, allowing for more precise corrections.

High-Precision Deformable Mirrors: DMs are central to the operation of AO systems, as they physically adjust to correct distortions in the optical wavefront. Future advancements will focus on developing high-precision DMs that can respond more quickly and accurately to detected distortions. This includes the exploration of new actuator technologies and mirror materials that offer improved performance characteristics. Additionally, research will aim to increase the number of actuators on DMs, providing finer control over the mirror surface and enabling better correction of complex wavefront distortions.

##### Integration of Machine Learning Algorithms

ML algorithms are anticipated to be pivotal in developing predictive and dynamic AO systems with real-time optimization.

Predictive and Dynamic AO Systems: ML algorithms are anticipated to play a significant role in optimizing AO systems by predicting and compensating for dynamic environmental changes. ML can be used to analyze historical and real-time data to predict atmospheric conditions and preemptively adjust the AO system. For example, neural networks can be trained to recognize patterns in AT and optimize the response of wavefront sensors and DMs accordingly. This predictive capability will enhance the robustness and efficiency of FSO communication by reducing latency and improving correction accuracy [389].

Real-Time Optimization: ML algorithms can also be employed for the real-time optimization of AO systems. By continuously learning from the incoming data and adjusting system parameters on-the-fly, these algorithms can ensure optimal performance under varying conditions. This dynamic adaptation will be particularly valuable for mobile FSO platforms, such as UAVs, where environmental conditions can change rapidly.

##### Miniaturization of AO Components

The miniaturization of AO components is essential for integrating these systems into a variety of use cases.

Compact and Mobile Platforms: Miniaturizing AO components is crucial for integrating these systems into compact and mobile platforms, such as UAVs, satellites, and portable ground stations. Future research will focus on developing smaller, lighter, and more energy-efficient wavefront sensors and DMs without compromising performance. This involves leveraging progress in MEMS technology and nanotechnology to create miniaturized components that can be easily integrated into various platforms.

Scalable AO Systems: The miniaturization of AO components will also enable the development of scalable AO systems that can be deployed across a broad spectrum of applications, from small handheld devices to large-scale ground stations. Scalable systems will offer greater flexibility and adaptability, making AO technology accessible for diverse FSO communication scenarios.

##### Hybrid Adaptive Optics Techniques

Investigating hybrid AO techniques can offer more comprehensive solutions for FSO communication systems.

Combining Traditional and Novel Approaches: Exploring hybrid AO techniques that combine traditional methods with novel approaches, such as digital holography, can further enhance the robustness and efficiency of FSO communication. Digital holography involves capturing and reconstructing the wavefront information using digital sensors and processing algorithms. Integrating this technique with conventional AO methods can enhance the system’s ability to correct for complex distortions and improve overall performance.

Enhanced Turbulence Mitigation: Hybrid AO techniques can provide more comprehensive solutions for mitigating turbulence effects by leveraging the strengths of different approaches. For instance, digital holography can offer high-resolution wavefront reconstruction, while traditional AO methods can provide real-time correction. Combining these techniques can result in more effective turbulence mitigation and better communication link stability.

#### 6.4.8. Adaptive Detection Thresholds

The future of ADT schemes in FSO communication presents a wealth of opportunities aimed at improving system performance, reliability, and adaptability. As the demand for high-capacity and resilient communication networks continues to grow, the development of advanced ADT strategies will be critical. The following sections elaborate on the key avenues for future research and development in ADT schemes.

##### Integration of Machine Learning Techniques

One of the most promising directions for ADT schemes is the integration of ML techniques to enhance the adaptability and precision of detection thresholds.

Predictive Threshold Optimization: By leveraging real-time data, ML algorithms can predict the optimal thresholds required for different conditions, including varying noise levels and interference patterns. This predictive capability allows for proactive adjustments to the detection thresholds, ensuring that the system can maintain optimal performance even in dynamic environments.

Adaptive Learning Models: Implementing adaptive learning models that continuously refine their predictions based on incoming data will be essential. These models can be trained using historical data to recognize patterns in environmental changes and system performance, allowing them to adjust thresholds dynamically. Over time, the algorithms will improve their accuracy and responsiveness, making the communication link more reliable and efficient.

##### Development of Sophisticated Algorithms

Future research should concentrate on creating more sophisticated algorithms that can dynamically adjust detection thresholds in response to environmental changes.

Dynamic Threshold Adjustment: This involves creating algorithms that monitor various parameters, such as ambient light levels, atmospheric conditions, and interference sources, in real-time. By correlating these parameters with the performance metrics of the communication system, the algorithms can adapt the thresholds to optimize detection sensitivity and minimize false positives or negatives.

Robustness Against Noise and Interference: The ability to adjust detection thresholds based on the specific characteristics of noise and interference is crucial for improving system robustness. Advanced statistical methods, including Bayesian inference and stochastic modeling, can be employed to develop thresholds that are not only reactive but also proactive, enabling the system to anticipate potential performance degradation before it occurs. Such algorithms would be particularly beneficial in urban environments where interference from multiple sources is common.

##### Exploration of Hybrid ADT Schemes

Exploring hybrid ADT schemes that combine multiple detection strategies will be another key area for future research.

Combining Detection Strategies: By integrating different detection approaches, such as energy detection, matched filtering, and decision feedback techniques, hybrid schemes can achieve improved performance across a broader range of operating conditions. This approach can leverage the strengths of each method while mitigating their weaknesses, leading to a more robust overall detection mechanism.

Adaptive Feedback Mechanisms: Hybrid ADT schemes can also include adaptive feedback mechanisms that allow for the real-time adjustment of detection strategies based on performance outcomes. For example, if one detection method is underperforming due to a sudden increase in noise, the system can switch to another method that may be more suitable under the current conditions. This flexibility will enhance the system’s overall reliability and efficiency.

##### Broadening the Application of ADT Schemes

Expanding the application of ADT schemes to emerging communication technologies, such as 5G and beyond, will ensure their relevance and effectiveness in next-generation networks.

Relevance in Next-Generation Networks: As FSO communication is increasingly integrated into 5G architectures for high-speed data transmission, ADTs will play a crucial role in managing the complexities of multi-user environments, varying signal quality, and high data rates.

Support for Massive Machine-Type Communications: The rise in mMTC and the IoT presents unique challenges for detection schemes. ADT strategies will need to adapt to the high density of devices and the potential for increased interference. The research will focus on developing ADT mechanisms that can efficiently manage these challenges, ensuring that each device can maintain reliable communication despite the presence of numerous concurrent transmissions.

#### 6.4.9. Hybrid FSO Systems

Hybrid FSO systems are poised to revolutionize optical communication by leveraging the unique strengths of FSO alongside other complementary technologies. These systems address individual limitations, offering enhanced performance in various environmental conditions. The future of hybrid FSO systems will revolve around several key research areas aimed at optimizing integration, enhancing reliability, and supporting the next generation of communication technologies.

##### Exploring Seamless Communication Solutions

The exploration of hybrid FSO-RF systems offers a promising avenue for ensuring seamless communication across diverse environments. By combining the high-capacity and low-latency characteristics of FSO with the robustness and reliability of RF technologies, these systems can maintain connectivity even in the face of adverse atmospheric conditions. Future research should prioritize the development of intelligent algorithms that can determine the most suitable transmission medium based on environmental assessments, user demands, and application requirements.

##### Optimizing Technology Integration

The synergy between FSO and alternative technologies will allow for redundancy and resilience in communication networks. This flexibility will be vital in applications requiring uninterrupted service, such as critical communications for emergency services and real-time data transmission for autonomous vehicles. One way to achieve this is by implementing advanced handover protocols and algorithms to enhance the reliability of hybrid systems.

Seamless Handover Mechanisms: Future research will focus on developing seamless handover mechanisms between hybrid FSO systems and complementary communication technologies, such as RF and microwave links. Achieving smooth transitions between different transmission mediums is critical for maintaining continuous connectivity, particularly in mobile scenarios. By implementing advanced handover protocols and algorithms, hybrid systems can automatically switch between FSO and RF links based on real-time assessments of signal quality, distance, and atmospheric conditions. This ensures uninterrupted service, even in challenging environments.

Enhanced Reliability and Resilience: Improving the reliability of hybrid systems is essential, especially in the face of AT and link blockages. Future studies will explore the integration of robust error correction methods and adaptive modulation techniques that can respond dynamically to environmental fluctuations. The use of intelligent algorithms to assess real-time conditions and adjust transmission parameters accordingly will be crucial. For instance, adaptive modulation schemes can vary the modulation format and coding rate based on current channel conditions, enhancing data throughput while minimizing errors.

##### Innovations in Adaptive Modulation and Beam Steering

Innovations in adaptive modulation are crucial for maximizing the performance of hybrid FSO systems. Beam steering technology is another critical area of development.

Adaptive Modulation Techniques: By employing techniques that can dynamically adjust modulation schemes depending on the quality of the communication link, these systems can optimize data rates and reliability. Future research may focus on integrating ML algorithms to predict optimal modulation schemes based on historical and real-time data, enabling systems to adapt more effectively to varying conditions.

Advanced Beam Steering Solutions: Future hybrid FSO systems will benefit from advanced beam steering mechanisms that enhance the alignment of the optical beam, compensating for the effects of turbulence and movement. Techniques such as phased array systems and smart optics can be employed to achieve high-precision steering, ensuring that the optical signal remains focused on the intended Rx. The integration of intelligent beam steering algorithms will allow these systems to respond to environmental changes dynamically, further improving performance.

##### Intelligent Network Management

As hybrid FSO systems become more prevalent, intelligent network management will be essential to optimize resource allocation and enhance overall network performance. Also, ensuring QoS is paramount in hybrid FSO systems, especially as user demands for high-speed, low-latency communication increase.

Smart Network Orchestration: Future research will likely focus on developing advanced orchestration algorithms that can analyze network traffic, device connectivity, and environmental conditions to optimize the distribution of data across different transmission mediums. This will involve the employment of AI and ML techniques to create predictive models that facilitate efficient network management and minimize latency.

Quality-of-Service Enhancements: Future research will explore QoS management techniques that prioritize traffic based on application requirements, ensuring that critical applications such as real-time video conferencing and remote surgery receive the necessary bandwidth and reliability. These enhancements will be vital for supporting a diverse range of applications across hybrid communication networks.

##### Development of Compact and Cost-Effective Transceivers

The development of compact, low-power, and cost-effective transceivers will be pivotal in making hybrid FSO systems more accessible and practical for widespread deployment. Also, addressing the cost barriers associated with deploying hybrid FSO systems will also be a significant area of focus.

Miniaturization and Integration: Future research will focus on miniaturizing optical components and integrating them into single packages, reducing the overall footprint and power requirements. Advances in photonics and microfabrication technologies will facilitate the creation of lightweight and energy-efficient transceivers that can be easily installed in various environments, from urban infrastructures to remote rural areas.

Cost Effectiveness for Broad Deployment: Research will aim to streamline manufacturing processes and explore innovative funding models to make these technologies affordable for various applications, including municipal networks, broadband access, and IoT infrastructures. The availability of cost-effective solutions will accelerate the adoption of hybrid FSO systems across different market segments.

##### Supporting Emerging Applications

With the advancement of hybrid FSO systems, they will possess significant potential to support emerging applications.

5G/6G Networks and IoT Integration: As hybrid FSO systems evolve, their potential to support emerging applications like 5G and 6G networks, as well as the IoT, will be significant. The integration of hybrid systems into these next-generation networks will enable uRLLC, and facilitate advanced applications such as autonomous vehicles, smart cities, and augmented reality. By providing high-capacity links that can dynamically adapt to varying conditions, hybrid FSO systems will be integral in meeting the demands of an increasingly connected world.

Satellite Communications Synergy: Furthermore, hybrid FSO systems will play a crucial role in enhancing satellite communications by offering high-speed, low-latency links between satellites and ground stations. By combining FSO with traditional RF communication, satellite systems can achieve greater resilience and capacity, addressing the challenges of high-speed data transfer in diverse atmospheric conditions.

Moreover, hybrid free-space optical communication systems hold significant promise for the future of Space–Air–Ground–Sea Integrated Networks (SAGSINs). These systems offer high bandwidth, low latency, and secure data transmission, making them ideal for enhancing connectivity in SAGSINs. Future advancements are expected to focus on improving link reliability, atmospheric compensation techniques, and seamless integration with existing RF systems to ensure robust, high-speed communication across diverse environments.

## 7. Conclusions

In conclusion, FSO communication represents a transformative technology poised to address the escalating demands for high-speed, low-latency communication in the 5G and beyond era. This survey has highlighted the critical enabling technologies, such as adaptive optics, advanced modulation schemes, and robust error correction codes, that are essential for overcoming the inherent challenges associated with FSO systems, including atmospheric disturbances and alignment issues. The exploration of hybrid solutions integrating FSO with radio frequency, millimeter-wave, and Terahertz technologies demonstrates the potential for enhancing network reliability and coverage. Moreover, the incorporation of artificial intelligence and machine learning techniques marks a significant trend toward optimizing FSO performance in dynamic environments, paving the way for smarter and more resilient communication systems. By systematically analyzing current trends and challenges, this paper underscores the capacity of FSO to not only meet but exceed the communication requirements of future networks. Looking ahead, several open challenges remain, particularly concerning scalability, deployment strategies, and real-time adaptive capabilities in diverse atmospheric conditions. Addressing these challenges will be pivotal in realizing the full potential of FSO technology. Future research should focus on developing innovative solutions to enhance the robustness and efficiency of FSO systems, ensuring their successful integration into next-generation communication infrastructures. Ultimately, the insights presented in this paper contribute to a deeper understanding of the role of FSO in revolutionizing wireless communication and set the stage for ongoing advancements in this promising field.

## Figures and Tables

**Table 1:** Fundamental differences between non-coherent (IM/DD), coherent, and self-coherent FSO systems.

Feature	Non-Coherent	Coherent	Self-Coherent
Modulation Techniques	Intensity modulation	Amplitude, frequency, or phase modulation	Phase modulation
DSP Complexity	Low	High	Moderate
Receiver Complexity	Low	High	Moderate
Detection Mechanism	Direct detection of light intensity	Optical mixing with a locally generated optical field	Optical mixing with a delayed version of the received signal
Need for polarization control or diversity	No	Yes	Yes
Need for Local Oscillator	No	Yes	No
Noise Rejection Performance	Moderate	Superior	High
Sensitivity to Atmospheric Turbulence	Higher	Lower	Lower
Receiver Sensitivity	Moderate	High	High
Spectral efficiency	Lower	Higher	Moderate
Reach	Lower	Higher	Moderate
Power consumption	Lower	Higher	Moderate
Footprint	Lower	Higher	Moderate
Cost	Lower	Higher	Moderate
Common Usage	Terrestrial FSO links	Advanced applications requiring high performance	Applications needing a balance of performance and complexity

**Table 2:** Unified M-distribution framework for various FSO models (adapted from [99]).

Conditions										
**Distribution Model**	ρ	$\mathrm{Var}\left[|U_{L}|\right]$	$\mathrm{Var}\left[G\right]$	X	$\Omega^{\prime }$	$\mathrm{Var}\left[X\right]$	γ	Ω	α	β
Rice-Nakagami	0	0								
Gamma	0						0			
Homodyned K	0		0	γ						
Γ Γ	1				1		0			
Shadowed-Rician						0				
Log-normal	0	0					→0			
K	0							0		
Exponential	0	0						0	→∞	
Gamma-Rician										→∞

## Abbreviations

The following abbreviations are used in this manuscript:

2D	Two-Dimensional
3D	Three-Dimensional
5G	Fifth Generation
6G	Sixth Generation
ABER	Average BER
ADC	Analog-to-Digital Converter
ADT	Adaptive Detection Threshold
AF	Amplify-and-Forward
AGC	Automatic Gain Control
AI	Artificial Intelligence
AMC	Adaptive Modulation and Coding
ANN	Artificial Neural Network
AO	Adaptive Optics
APDs	Avalanche PDs
APSK	Amplitude PSK
ASC	Average Secrecy Capacity
ASK	Amplitude Shift Keying
AT	Atmospheric Turbulence
AWGN	Additive White Gaussian Noise
BA	Buffer-Aided
BER	Bit-Error Rate
BF	Buffer-Free
BICM	Bit-Interleaved CM
BPOLSK	Binary POLSK
BPSK	Binary PSK
BSI	Buffer State Information
CNNs	Convolutional Neural Networks
CPAT	Coarse PAT
CSI	Channel State Information
CSPR	Carrier-to-Signal Power Ratio
DAC	Digital-to-Analog Converter
DD	Direct Detection
DF	Decode-and-Forward
DFB	Distributed Feedback
DL	Deep Learning
DM	Deformable Mirror
DMT	Discrete Multi-Tone
DNN	Deep Neural Network
DoF	Degrees of Freedom
DPSK	Differential PSK
DQPSK	Differential Quadrature PSK
DRE	Digital Resolution Enhancer
DRL	Deep Reinforcement Learning
DSP	Digital Signal Processing
e2e	End-to-End
ECC	Error Correction Code
EGC	Equal Gain Combining
EW	Exponentiated Weibull
FDT	Fixed Detection Threshold
FEC	Forward Error Correction
FER	Frame Error Rate
FFT	Fast Fourier Transform
FoV	Field-of-View
FP	Fabry–Pérot
FSK	Frequency Shift Keying
FSM	Fast Steering Mirror
FSO	Free-Space Optical
Ge	Germanium
GNN	Graph Neural Network
GRU	Gated Recurrent Unit
GS	Geometric Shaping
HARQ	Hybrid Automatic Repeat Request
HCS	Hybrid Constellation Shaping
HQAM	Hexagonal QAM
i.i.d.	Independent and Identically Distributed
IAS	Inter-Antenna Synchronization
ICI	Inter-Channel Interference
IEC	International Electrotechnical Commission
IEEE	Institute of Electrical and Electronics Engineers
IFFT	Inverse Fast Fourier Transform
IM	Intensity Modulation
IM/DD	Intensity Modulation with Direct Detection
InGaAs	Indium Gallium Arsenide
IR	Infrared
ITU	International Telecommunication Union
KK	Kramers–Kronig
LD	Laser Diode
LDPC	Low-Density Parity Check
LED	Light-Emitting Diode
LG	Laguerre–Gaussian
Li-Fi	Light Fidelity
LN	Log-Normal
LO	Local Oscillator
LoS	Line-of-Sight
m.g.f.	Moment Generating Function
MAC	Medium Access Control
MDPSK	Multilevel DPSK
MEMS	Micro-Electro-Mechanical Systems
MIMO	Multiple-Input Multiple-Output
MISO	Multiple-Input Single-Output
ML	Machine Learning
MLSD	Maximum-Likelihood Sequence Detection
mMTC	Massive Machine-Type Communications
mmWave	Millimeter-Wave
M-PAM	M-ary PAM
MPOLSK	Multilevel POLSK
MPSK	Multilevel PSK
M-QAM	*M*-ary QAM
MRC	Maximum Ratio Combining
MSM	Multiple-Subcarrier Modulation
MTBF	Mean Time Between Failures
NBA	Non-Buffer-Aided
NE	Negative Exponential
NGMI	Normalized Generalized Mutual Information
NLoS	Non-LoS
OAM	Orbital Angular Momentum
OBFSK	Orthogonal Binary FSK
OE	Optical-to-Electrical
OESM	Optically Enhanced SM
OFDM	Orthogonal Frequency-Division Multiplexing
OGSM	Optical Generalized SM
OIQSM	Optically Improved Quadrature SM
OOK	On–Off Keying
OP	Outage Probability
Op-Amp	Operational Amplifier
OSM	Optical Spatial Modulation
OSSK	Optical Space Shift Keying
OSTBCs	Orthogonal Space–Time Block Codes
OWC	Optical Wireless Communication
p2p	Point-to-Point
PAM	Pulse-Amplitude Modulation
PAPR	Peak-to-Average-Power Ratio
PAS	Probabilistic Amplitude Shaping
PAT	Pointing, Acquisition, and Tracking
PD	Photodiode
PDF	Probability Density Function
PDM	Polarization Division Multiplexing
PE	Pointing Errors
PHY	Physical
PICs	Photonic Integrated Circuits
PIN	Positive–Intrinsic–Negative
PLL	Phase-Locked Loop
PLS	PHY Layer Security
PolM	Polarization Modulation
POLSK	Polarization Shift Keying
PPM	Pulse-Position Modulation
PS	Probabilistic Shaping
PSAM	Pilot-Symbol Assisted Modulation
PSF	Point Spread Function
PSK	Phase-Shift Keying
PSP	Per-Survivor Processing
QAM	Quadrature Amplitude Modulation
QCDs	Quantum Cascade Detectors
QCLs	Quantum Cascade Lasers
QKD	Quantum Key Distribution
QoS	Quality of Service
RC	Repetition Coding
RF	Radio Frequency
RoFSO	Radio over FSO
RQAM	Rectangular QAM
RS	Reed–Solomon
Rx	Receiver
SC	Selection Combining
SDM	Space Division Multiplexing
SDT	Sparse–Dense Transmission
SE	Spectral Efficiency
SER	Symbol Error Rate
Si	Silicon
SIM	Subcarrier Intensity Modulation
SIMO	Single-Input Multiple-Output
SINR	Signal-To-Interference-Plus-Noise Ratio
SISO	Single-Input Single-Output
SLMs	Spatial Light Modulators
SM	Spatial Modulation
SNR	Signal-to-Noise Ratio
SOP	Secrecy Outage Probability
SPSC	Strictly Positive Secrecy Capacity
S-PSK	Subcarrier PSK
SQAM	Squared QAM
SSB	Single-Sideband
SSBI	Signal–Signal Beat Interference
SSBN	Signal-to-Signal Beating Noise
STC	Space–Time Coding
STD	Selection Transmit Diversity
STTC	Space–Time Trellis Coding
SVM	Support Vector Machines
SWIPT	Simultaneous Wireless Information and Power Transfer
TCM	Trellis-Coded Modulation
THz	Terahertz
TIA	Trans-Impedance Amplifier
TLS	Transmit Laser Selection
Tx	Transmitter
UAVs	Unmanned Aerial Vehicles
UOWC	Underwater OWC
uRLLC	Ultra-Reliable Low-Latency Communication
UV	Ultraviolet
VCSELs	Vertical-Cavity Surface-Emitting Lasers
VLC	Visible-Light Communication
WDM	Wavelength Division Multiplexing
XQAM	Cross QAM
